# High-Pressure and High-Temperature Chemistry of Phosphorus and Nitrogen: Synthesis and Characterization of α- and γ-P_3_N_5_

**DOI:** 10.1021/acs.inorgchem.2c01190

**Published:** 2022-07-26

**Authors:** Matteo Ceppatelli, Demetrio Scelta, Manuel Serrano-Ruiz, Kamil Dziubek, Fernando Izquierdo-Ruiz, J. Manuel Recio, Gaston Garbarino, Volodymyr Svitlyk, Mohamed Mezouar, Maurizio Peruzzini, Roberto Bini

**Affiliations:** † LENS, European Laboratory for Non-linear Spectroscopy, Via N. Carrara 1, I-50019 Sesto Fiorentino, Firenze, Italy; ‡ ICCOM-CNR, Institute of Chemistry of OrganoMetallic Compounds, National Research Council of Italy, Via Madonna del Piano 10, I-50019 Sesto Fiorentino, Firenze, Italy; § Malta-Consolider Team and Departamento de Química Física y Analítica, Universidad de Oviedo, Avda. Julián Clavería, 8, 33006 Oviedo, España; ∥ Department of Chemistry and Chemical Engineering, Chalmers University of Technology, Gothenburg 412 96, Sweden; ⊥ ESRF, European Synchrotron Radiation Facility, 71 Avenue des Martyrs, CS40220, 38043 Grenoble Cedex 9, France; # Dipartimento di Chimica “Ugo Schiff” dell’Università degli Studi di Firenze, Via della Lastruccia 3, I-50019 Sesto Fiorentino, Firenze, Italy

## Abstract

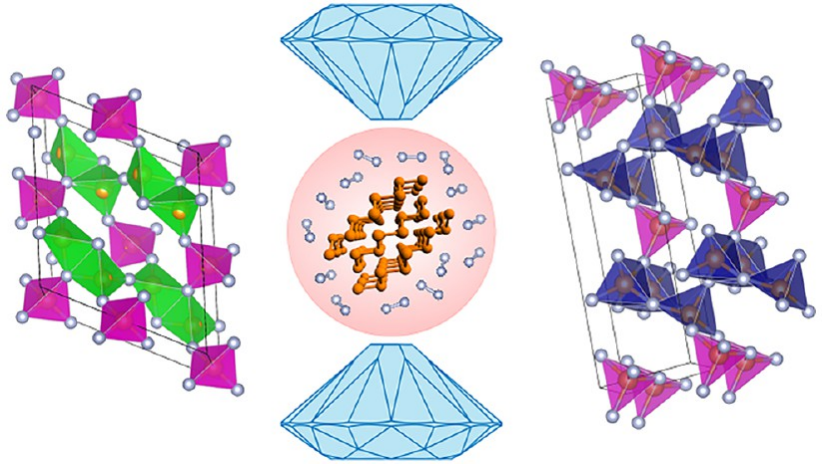

The direct chemical reactivity between phosphorus and nitrogen was induced under high-pressure and high-temperature conditions (9.1 GPa and 2000–2500 K), generated by a laser-heated diamond anvil cell and studied by synchrotron X-ray diffraction, Raman spectroscopy, and DFT calculations. α-P_3_N_5_ and γ-P_3_N_5_ were identified as reaction products. The structural parameters and vibrational frequencies of γ-P_3_N_5_ were characterized as a function of pressure during room-temperature compression and decompression to ambient conditions, determining the equation of state of the material up to 32.6 GPa and providing insight about the lattice dynamics of the unit cell during compression, which essentially proceeds through the rotation of the PN_5_ square pyramids and the distortion of the PN_4_ tetrahedra. Although the identification of α-P_3_N_5_ demonstrates for the first time the direct synthesis of this compound from the elements, its detection in the outer regions of the laser-heated area suggests α-P_3_N_5_ as an intermediate step in the progressive nitridation of phosphorus toward the formation of γ-P_3_N_5_ with increasing coordination number of P by N from 4 to 5. No evidence of a higher-pressure phase transition was observed, excluding the existence of predicted structures containing octahedrally hexacoordinated P atoms in the investigated pressure range.

## Abstract 2

The α- and γ-P_3_N_5_ phosphorus nitride polymorphs have been obtained by direct chemical reactivity between phosphorus and molecular nitrogen under high pressure (9.1 GPa) and high-temperature (2000−2500 K) conditions, generated using a laser heated diamond anvil cell. Insights on the reaction mechanism, involving the preliminary formation of α-P_3_N_5_, and on the structural properties of γ-P_3_N_5_ have been evinced by synchrotron X-ray diffraction, Raman spectroscopy and DFT calculations.

## Introduction

Nitrogen and phosphorus respectively occupy the second and third periods of group 15 in the periodic table of the elements. While exhibiting the characteristic *n*s^2^*n*p^3^ outer-shell electronic configuration, compared to heavier elements of the following periods, the two lowest-*Z* pnictogens do not have electrons in the d orbitals. P and N elements are widely present respectively in the earth’s crust and atmosphere, playing a key role in the existence of life on our planet, and are well known to chemists for their ability to combine with other atoms, forming a large variety of binary compounds (i.e., nitrides and phosphides). Nevertheless, whereas the chemistry literature and textbooks report a large variety of systems containing P–N bonds in the presence of other elements, studies on compounds containing only P and N are, in comparison, quite limited. In the Corbridge reference textbook on phosphorus, which is more than 1300 pages long, the binary compounds of phosphorus and nitrogen barely occupy 2 pages, in which just a few studies, essentially focused on phosphorus nitrides (PN and P_3_N_5_) and phosphorus triazide P(N_3_)_3_, are mentioned.^1,2^ Furthermore, whereas chemists have become quite expert at synthesizing compounds containing P–N bonds starting from suitable precursors,^3^ the elusive direct chemical reactivity between the two elements has been essentially reported only in the gas phase at high temperature and under electric discharge, leading to poorly characterized amorphous PN and P_3_N_5_ materials.^4,5^ The two elements do not react spontaneously under ambient conditions, and even if structural consistency has been recently found for group 15 elements at high pressure,^6−9^ there is a large interval of pressure and temperature conditions in which N and P behave quite differently. The thermodynamically stable allotrope of phosphorus at ambient conditions is the black form of the element (P_black_), which was synthesized for the first time at high pressure by Bridgman back in 1914^10^ and is currently attracting strong and growing attention from the scientific community because of its characteristic crystalline structure made by the stacking of phosphorene layers.^11,12^

The phase diagram of phosphorus at room temperature is known up to 340 GPa.^13^ P_black_, with the A17 layered orthorhombic structure (*Cmce*, *Z* = 8), is observed up to about 5 GPa, where it transforms to rhombohedral A7 (*R*3̅*m*, *Z* = 2), another phase with layered structure, which is stable up to 10.5 GPa. Above this pressure, a pseudosimple cubic structure (p-sc), extending from 10.5 up to about 30 GPa, has been recently demonstrated to exist using synchrotron X-ray diffraction (XRD) and He as the pressure-transmitting medium (PTM).^6^ The observation of this structure has significantly raised the pressure limit at which the layered structures of P can be observed, with remarkable implications.^7^ Upon further compression, a simple cubic (sc) structure, rarely observed in nature, is reported to be stable up to 107 GPa, where it transforms to an incommensurately modulated structure *Cmmm*(00γ)s00. At 137 GPa, this structure converts to a simple hexagonal one (P-V, sh, *P*6/*mmm*, *Z* = 1), observed up to 282 GPa, whereas a body-centered cubic structure (bcc), later identified as a superlattice structure (P-VI, cI16(*I*4̅3*d*)), has been observed from 262 GPa up to 340 GPa.^13^ Experimental high-pressure and high-temperature data are limited to ∼11 GPa and ∼1250 K.^14^

Nitrogen, on the other side, remains stable as a diatomic N_2_ molecule over a wide range of pressure along room-temperature compression from the fluid under ambient conditions through different molecular crystalline structures β, δ, δ_loc_, ϵ, and ζ up to ∼100 GPa.^15^ Above 100 GPa, the higher-density conditions further reduce the intermolecular distances, determining the instability of the N_2_ molecular units and leading to N amorphization.^16^ Laser heating (LH) at these pressures leads to the synthesis of different polymeric single-bonded structures of N: cubic-gauche N (*cg*-N; 110 GPa and 2000 K),^17^ layered polymeric N (LP-N; *Pba*2, above 125 GPa, and 2000 K),^18^ and hexagonal layered polymeric N (HLP-N; *P*4_2_*bc*, 240 GPa and 3300 K).^19^ Recently, two research groups independently and almost simultaneously reported N to adopt a layered crystalline structure analogous to the orthorhombic layered structure of black phosphorus (bp-N) upon laser heating above 140 GPa (140 GPa, 4000 K^8^ and 146 GPa, 2200 K^9^), thus extending to N the structural consistency within group 15, as recently occurred in the case of P with respect to heavier pnictogens.^7^ However, whereas the high-pressure behaviors of P and N have been constantly investigated by high-pressure scientists for years, the chemical reactivity between the two elements in the condensed phase has remained mostly unexplored until recently.

Investigating the chemical reactivity between P and N under extreme pressure and temperature conditions is not only important for providing insights about the fundamental chemistry of these two elements and shedding light on specific issues in bond theory in pnictogens under high-density conditions but is also relevant to current hot topics regarding the possibility of using pressure to synthesize new advanced materials that are potentially recoverable under ambient conditions, such as N-doped phosphorene-based materials,^20^ N-based high-energy-density materials, and new crystalline phosphorus nitrides featuring high P coordination by N atoms, which have been predicted by calculations^21−23^ and recently synthesized at high pressure in ternary systems.^24^

In this study, we successfully induced chemical reactivity between P and N using pressure, statically generated by means of a membrane diamond anvil cell (DAC), to increase the density and reduce the intermolecular distances and temperature, generated by laser heating, to overcome kinetic barriers. Within this picture, P was used as a reactant and laser absorber, whereas N_2_ was used as a reactant and PTM, thus avoiding any unnecessary contamination source. The same approach was successfully adopted to induce chemical reactivity in P and H_2_, leading to the synthesis of PH_3_ and to the discovery of the crystalline van der Waals compound (PH_3_)_2_H_2_^25^ and, more recently, to the synthesis of crystalline arsenic nitride (AsN) from As and N_2_.^26^

While this manuscript was in preparation, a paper on the same topic was published by a Japanese group led by Hasegawa.^27^ Whereas some findings of the recent paper are consistent with our previous results,^28,29^ relevant differences apply, leading to contrasting conclusions, particularly concerning the comprehension of the reactive process and the characterization of the products.

Our XRD and Raman data indicate that, under the applied high-pressure and high-temperature conditions, the formation of chemical bonds between the two lowest-Z pnictogens leads to the synthesis of P_3_N_5_. Although the process ultimately leads to the formation of γ-P_3_N_5_, α-P_3_N_5_ also seems to be involved, likely as a preliminary step. This observation, in agreement with the pressure-coordination rule, is consistent with the original high-pressure and high-temperature synthesis of γ-P_3_N_5_ reported by Landkskron et al.^30^ The formation of α-P_3_N_5_ from the elements, without any precursor, has not been reported so far.

γ-P_3_N_5_ is here further characterized by synchrotron X-ray diffraction (XRD), Raman spectroscopy, and density functional theory (DFT) calculations at both high and ambient pressure to gain insight into its mechanical properties and to identify potential reactive paths toward the predicted formation of higher-pressure polymorphs with octahedral P coordination by N atoms.^21,22,31^

## Experimental Section

Pure crystalline P_black_ used for the experiments was synthesized from red phosphorus according to ref (32). The reagents for the synthesis of P_black_ were purchased from Sigma-Aldrich with the following purities: red phosphorus (>99.99%), tin (>99.999%), gold (>99.99%), and SnI_4_ (99.999%). The purity of the synthesized P_black_ crystals used in this study was checked by X-ray powder diffraction, Raman spectroscopy, EDX analysis, and ICP-MS measurements, with the latter giving a purity of 99.999+%. The P_black_ crystals were then fragmented and cut by a metallic tip to obtain smaller 20–40 μm chips to be loaded into the DAC.

Pressure was generated by means of membrane diamond anvil cells (DAC) equipped with Ia/IIa type standard cut 16-sided beveled anvils having 350/400 μm culet tips. Re gaskets (200 μm) indented to about 50 μm and laser-drilled to obtain a 150-μm-diameter hole were used for the sideways containment of the samples. A small crystal of P_black_ (∼30 μm) was placed in the sample chamber by means of a metallic tip, and the remaining volume was filled with liquid N_2_ using a standard gas-loading technique. Au and a ruby chip were used to measure the pressure, whereas the temperature was estimated by the fit of thermal radiation during laser heating. High temperature was generated by means of a Nd:YAG laser source (λ = 1054 nm) focused on the P_black_ crystal (∼30 μm beam spot size diameter).

The samples were studied using synchrotron XRD at ESRF-ID27 with monochromatic λ = 0.3738 Å and a beam spot size diameter of 5 μm during and after LH to select different areas of the sample, spot differently oriented P_black_ crystallites, and find the reflections necessary for the calculation of the lattice parameters and the unit cell volume. The diffracted radiation was revealed by a MAR CCD165 detector except for the pattern of the reaction product under ambient conditions, where a Dectris PILATUS 300 K-W detector was used. A typical acquisition time was 20–30 s with ±15° maximum oscillation. The detector tilt and sample-to-detector distance were calibrated with a CeO_2_ standard. The raw images were processed using DIOPTAS software.^33^

Except for one pressure point at 9.1 GPa, where the structure of the reaction product could be determined by Rietveld refinement, for the other high-pressure points the lattice parameters and unit cell volume were determined using the UnitCell software.^34^ The peak positions were obtained by fitting the integrated XRD patterns with Voigt line shapes after baseline subtraction. Fityk software was used for this purpose.^35^ A multiphase full profile Le Bail fit was performed to obtain the structure of the reaction product recovered under ambient conditions (Figure SI-1).

The Raman spectra were acquired at LENS with 1.5 cm^–1^ spectral resolution using the 647.1 nm emission wavelength of a Kr ion laser. The details of the Raman setup are described elsewhere.^36^ No photochemical effect was observed at the employed laser power.

## Computational Methods

Electronic structure calculations were carried out at the GGA level using the exchange-correlation PBE functional^37^ with the periodic boundary conditions simulation suite Quantum Espresso, version 6.3.^38^ Projector-augmented wave^39^ pseudopotentials from the standard library^40^ were used for 1s^2^ and 1s^2^2s^2^2p^6^ core electrons of N and P, respectively. The kinetic energy cutoff for the plane waves was fixed to 80 Ry, and a 12 × 12 × 12 mesh was selected for integration in the reciprocal space. The self-consistent field convergence threshold was fixed to 10^–8^ Rydberg. Full cell geometrical optimizations were stopped using a force threshold of 10^–3^ Rydberg/Bohr. The equation-of-state parameters were obtained with the code GIBBS2^41^ by fitting a series of computed volume–energy pairs spanning a pressure range of up to more than 100 GPa. The vibrational frequencies of all P_3_N_5_ polymorphs were also obtained with Quantum Espresso. We performed calculations under the density functional perturbation theory approximation^42^ on a 2 × 2 × 2 supercell to describe the forces. We employed a 70 Ry energy cutoff for the plane waves and a *k*-point mesh of 6 × 6 × 6. Frequencies were associated with pressures thanks to the equations of states derived following the procedure described above.

## Results and Discussion

### High-Pressure and High-Temperature Reactivity between P and N_2_

**Figure 1:**
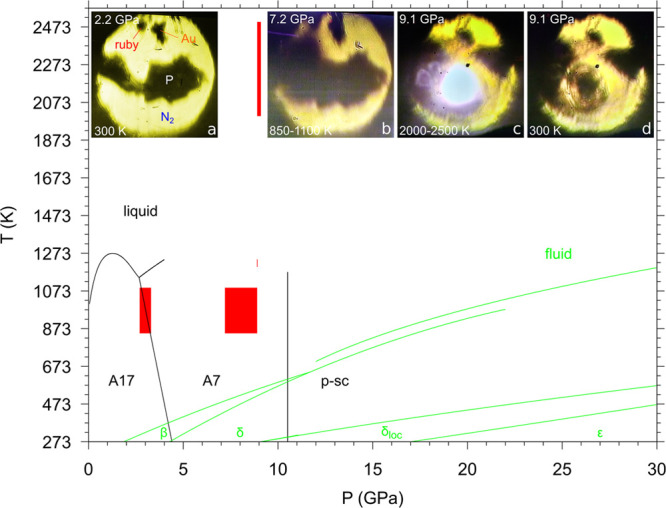
Phase diagram of phosphorus (black lines),^6,43^ and nitrogen (green lines)^16,44−46^ under laser heating conditions (red). The two melting lines of N_2_ at low and high pressure have been drawn according to Zinn et al.^44^ and Weck et al.,^45^ respectively. The images represent (a) a loaded sample before LH, (b) a sample at 7.2 GPa and ambient temperature after LH2 at 7.2–8.9 GPa and 850–1100 K, (c) a sample at 9.1 GPa and 2000–2500 K during LH4, and (d) a sample at 9.1 GPa and ambient T after LH4.

The phase diagrams of P and N from ambient conditions up to 30 GPa and ∼2500 K are reported in Figure 1. Pressure and temperature regions can be identified in which P is stable in one of its layered structures (A17, A7, and p-sc) and, simultaneously, molecular nitrogen is fluid. Laser heating in these regions was initially selected to preserve the layered structure of P while taking advantage of the higher mobility of fluid N_2_ toward N functionalization.

P_black_ was loaded with liquid N_2_ in a membrane DAC and compressed to the desired laser heating pressure (Experimental Section). Figure 1a shows a microscope image of a sample at 2.2 GPa and 300 K before laser heating, where P_black_ can be observed in the dark central area and N_2_ is in the surrounding transparent regions. The Au and ruby pressure gauges are also visible at the top of the picture.

**Figure 2:**
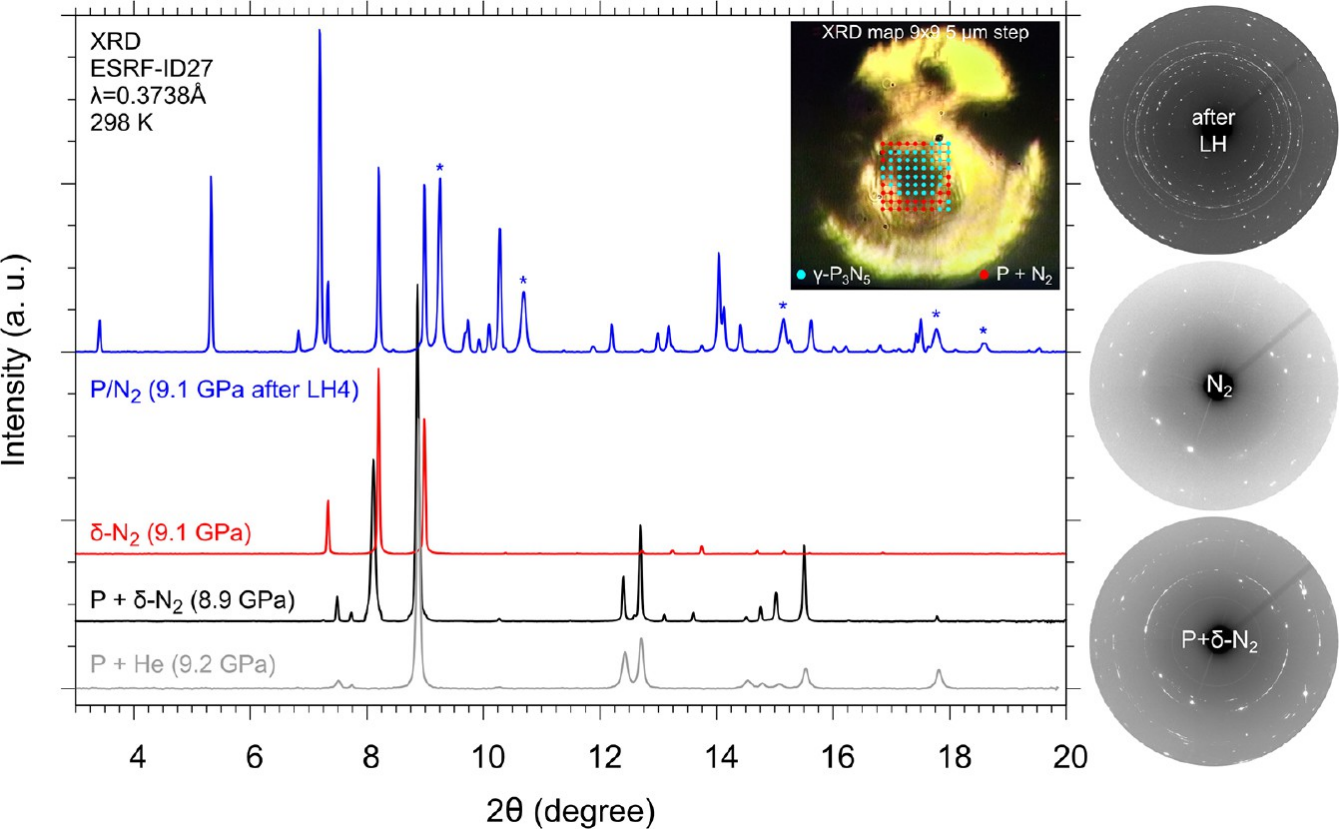
Integrated XRD diffraction patterns and corresponding detector images (right) acquired before LH2 in the dark (A7-P and δ-N_2_, black trace, bottom detector image) and in the transparent (δ-N_2_, red trace, middle detector image) areas of the sample (Figure 1b) and after LH4 at 9.1 GPa in the center of the laser-heated area (blue trace, top right image). The pattern of the reaction product (blue trace) was acquired in one of the blue points on the red XRD mapping grid superimposed on the image of the sample after LH4 at 8.9–9.1 GPa and 2000–2500 K. Each * indicates a peak from the Au pressure sensor. The integrated patterns have been normalized to their most intense peak, and the background has been subtracted. A diffraction pattern of a sample containing P in He at comparable pressure is also displayed (gray trace) to help identify the peaks from P (A7).

Several laser heating irradiations (Nd:YAG 1064 nm) were performed under different pressure conditions, corresponding to the A17 and A7 layered structures of P. No reactivity was observed at 3 GPa and *T* < 1100 K (LH1, 10–15 W) during the first laser heating in the A17 structure of P. Pressure was then increased up to 7.2 GPa in the A7 structure of P, where another laser irradiation was performed between 7.2 and 8.9 GPa and 850–1100 K (LH2, 10–19.5 W). After LH2, the areas corresponding to P appeared larger and more diffuse (Figure 1b). Nevertheless, no changes could be appreciated in the diffraction patterns. Another irradiation (LH3, 10–24.8 W), at about the same pressure (8.9 GPa) and slightly higher temperature (1200–1240 K), was responsible for the appearance of extremely weak new peaks in a few spots of the laser-heated area. Finally, irradiation (LH4, 10–35 W, ∼1 h) under comparable pressure conditions (8.9–9.1 GPa) but considerably higher temperature (2000–2500 K) produced a sudden lightning in the laser-heated area of the sample (Figure 1c). Correspondingly, the XRD diffraction patterns acquired at high temperature during laser heating clearly showed the appearance of new peaks, thus suggesting the occurrence of a chemical transformation. The effects of the irradiation were also evident at room temperature, after switching off the laser, both in the sample image (Figure 1d) and in the diffraction patterns (Figure 2).

To gain insight about the occurrence of chemical reactivity, we performed a 9 × 9 XRD mapping of the laser-heated area (5 μm spatial resolution), indicated by the red grid in the sample image shown in Figure 2. We found that whereas the outer part of the grid mainly contains unreacted P and N_2_ (red points on the grid), the inner part of the LH area essentially contains a reaction product (blue points on the grid) and unreacted excess N_2_ but not P, which was completely consumed here during LH. The spotty intensity distribution observed in the diffraction images acquired in the LH area indicates the formation of a powder-like polycrystalline reaction product (top right image in Figure 2), as typically occurring upon LH at high pressure, and the corresponding integrated pattern clearly shows the appearance of new peaks, none of which can be associated with any known structure of P (integrated patterns in Figure 2).

**Figure 3:**
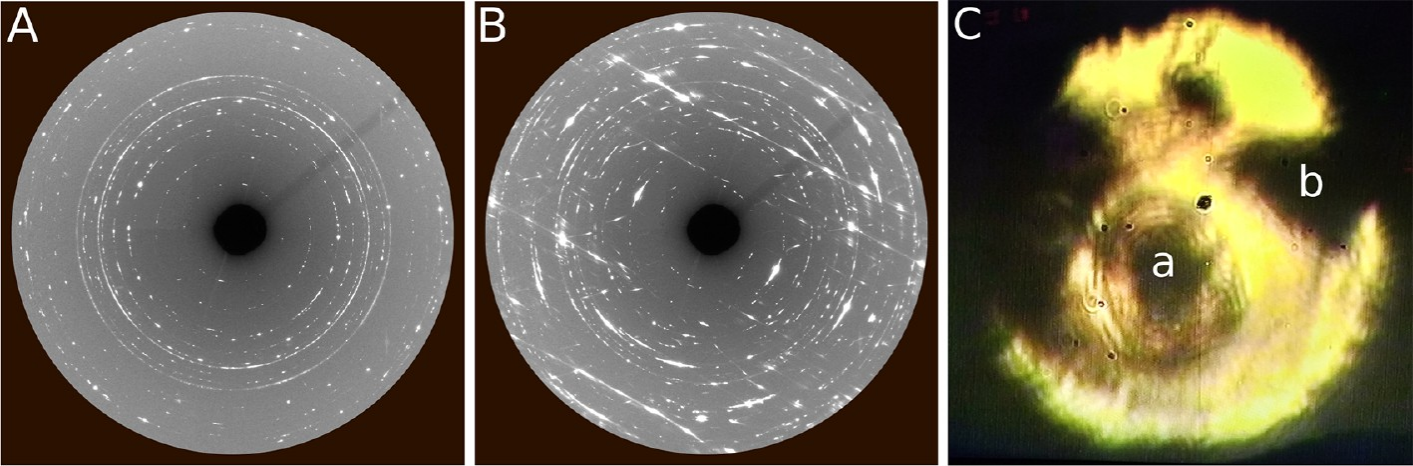
(A and B) Detector diffraction images acquired in the sample positions respectively corresponding to (C) labels a and b in the sample image.

The diffraction images acquired on the outer portions of the laser-heated area reveal instead the presence of a diffuse scattering pattern, which is characterized by the appearance of parallel stripes with periodic intensity oscillations, superimposed on the diffraction of P, N_2_, and the reaction product also observed in the center of the laser-heated area. This is clearly shown in Figure 3, displaying a comparison between one of the diffraction images acquired in the center of the laser-heated area (Figure 3A, the same as in the upper right panel of Figure 2) and one of those acquired on its borders, where a portion of P was partially heated by the laser spot (Figure 3B). Both signals remain present in the corresponding sample areas (sample positions a and b in Figure 3C) during compression up to the highest investigated pressure (32.6 GPa for XRD and 45.5 GPa for Raman) and decompression down to ambient pressure.

### Identification of the Reaction Products

Two forms of phosphorus(V) nitride, α-P_3_N_5_^47^ and γ-P_3_N_5_,^30^ have been experimentally synthesized and structurally characterized for the first time by Schnick et al. A third form, β-P_3_N_5_,^48^ was identified as a polymorph of α-P_3_N_5_ featuring a stacking disorder, whereas the existence of other P_3_N_5_ polymorphs, predicted by calculations, has never been experimentally observed so far.^21,22,31^

**Figure 4:**
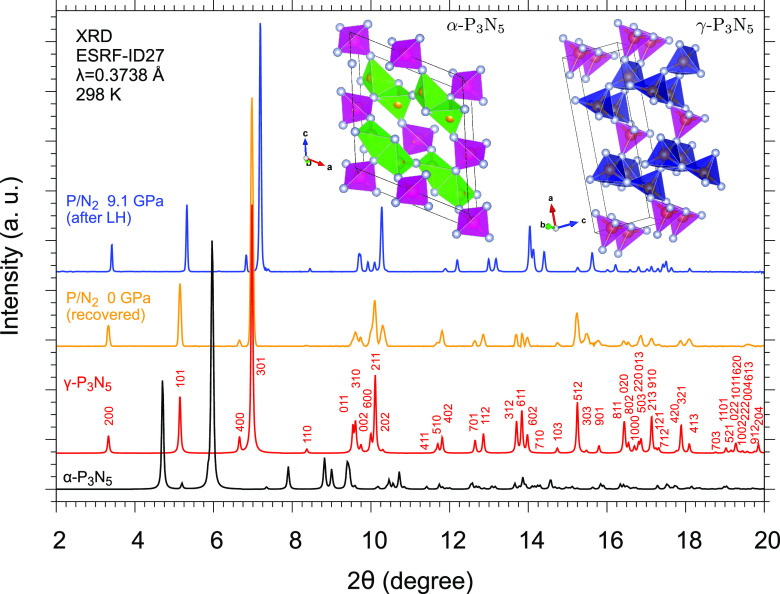
Room-temperature integrated XRD patterns of the reaction product at 9.1 GPa after LH4 (blue) and once recovered at ambient pressure (orange) and of α-P_3_N_5_ (black)^47^ and γ-P_3_N_5_ (red)^30^ under ambient conditions drawn according to the literature.^49,50^ The crystalline structures of α-P_3_N_5_ and γ-P_3_N_5_ are also displayed. To make the comparison more clear, the peaks of unreacted excess δ-N_2_ and Au (pressure sensor) were mathematically fitted and filtered out from the integrated pattern of the product acquired at 9.1 GPa after LH4 (blue trace in Figure 2).

α-P_3_N_5_ belongs to the *Cc* (*C*_*s*_^4^, no. 9) monoclinic space group (*Z* = 4) and has a density of 2.77 g cm^–3^ and a 3D structure made by corner- and edge-sharing PN_4_ tetrahedra (Figure 4). It was obtained as a mixture of α-P_3_N_5_ and β-P_3_N_5_ from the high-temperature reaction of suitable precursors^48^
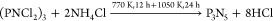
and as pure α-P_3_N_5_ from the thermal decomposition of tetradiaminophosphonium iodide according to the following reaction:^47^
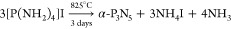


γ-P_3_N_5_ belongs to the *Imm*2 (*C*_2*v*_^20^, no. 44) orthorhombic space group (*Z* = 2) and has a density of 3.65 g cm^–3^, which is 32% higher than that of α-P_3_N_5_, and a 3D structure made by corner-sharing PN_4_ tetrahedra and by corner- and edge-sharing PN_5_ square pyramids (Figure 4). It was synthesized for the first time by Landskron and coauthors from partially crystalline α-P_3_N_5_ at 11 GPa and 1500 °C.^30^

Both the α and the γ phases of P_3_N_5_ can display ferroelectric behavior because *Cc* and *Imm*2 are polar space groups.

Denser P_3_N_5_ polymorphs, indicated as kyanite-type δ-P_3_N_5_ (triclinic *P*1̅) by Kroll et al.^21^ and as V_3_O_5_-like P_3_N_5_ by Dong et al.,^22^ have been predicted by calculations^21,22^ but have never been experimentally observed. These polymorphs feature PN_6_ units, where P is octahedrally coordinated by six N atoms. According to theoretical calculations, kyanite-type δ-P_3_N_5_ is expected to become stable with respect to γ-P_3_N_5_ above 28 and 43 GPa, respectively, depending on the adopted local density (LDA) or general gradient (GGA) approximation,^21^ whereas V_3_O_5_-like P_3_N_5_ is expected to be stabilized versus γ-P_3_N_5_ above 35.5 GPa.^22^ Whereas the δ-P_3_N_5_ structure features edge-sharing PN_6_ octahedra and isolated PN_4_ tetrahedra sharing their vertices with the octahedra, the V_3_O_5_-like one is entirely made by PN_6_ octahedra. In both cases, a higher coordination of P by the N atoms is induced by pressure with respect to the α-P_3_N_5_ and γ-P_3_N_5_ polymorphs. A 35% higher density has been estimated for the V_3_O_5_-like structure with respect to α-P_3_N_5_.^22^ Octahedral PN_6_ units have also been predicted to exist by Raza et al.^23^ in two high-pressure phosphorus nitride polymorphs having different stoichiometry: skutterudite-type PN_3_ (orthorhombic *Immm*), metastable between 10 and 100 GPa, and PN_2_ (*P*2/*m*), stable above 200 GPa. Also, these two polymorphs have never been experimentally reported. Experimentally, PN_6_ units have been recently synthesized in ternary systems including B besides P and N.^51^

The reaction products observed in the center of the laser-heated area and in its outer portion (Figure 3) were identified as γ-P_3_N_5_ and α-P_3_N_5_, respectively.

In Figure 4, the room-temperature XRD patterns of α-P_3_N_5_ (black) and γ-P_3_N_5_ (red) under ambient conditions, generated from literature cif files,^49,50^ are compared with those of our reaction product acquired in the center of the laser-heated area at 9.1 GPa (blue) and at ambient pressure (orange), once recovered. Despite a slightly larger peak width in our recovered sample, due to the use of a different detector with respect to the high-pressure data (Experimental Section), and despite intensity differences in some peaks, due to the polycrystalline nature of the product synthesized by HP-HT, perfect matching in terms of lattice parameters and the unit cell volume can be appreciated between the pattern of our recovered reaction product synthesized from P and N_2_ at high pressure and high temperature (*a* = 12.8757(5) Å, *b* = 2.61745(15) Å, *c* = 4.39920(15) Å, and *V*_0_ = 148.259(8) Å^3^, Figure SI-1) and the pattern of γ-P_3_N_5_ reported in the literature synthesized from α-P_3_N_5_ at ambient pressure and high temperature (*a* = 12.8721(4) Å, *b* = 2.61312(6) Å, *c* = 4.4003(2) Å, and *V*_0_ = 148.00(2) Å^3^).^50^

In most areas of the experimental chamber, the crystal growth during laser heating led to a spotty diffraction pattern unsuitable for structural refinement. However, by focusing the beam on one selected portion of the sample, data of sufficiently high quality could be identified to perform a full Rietveld refinement using JANA2006 software.^52^

**Figure 5:**
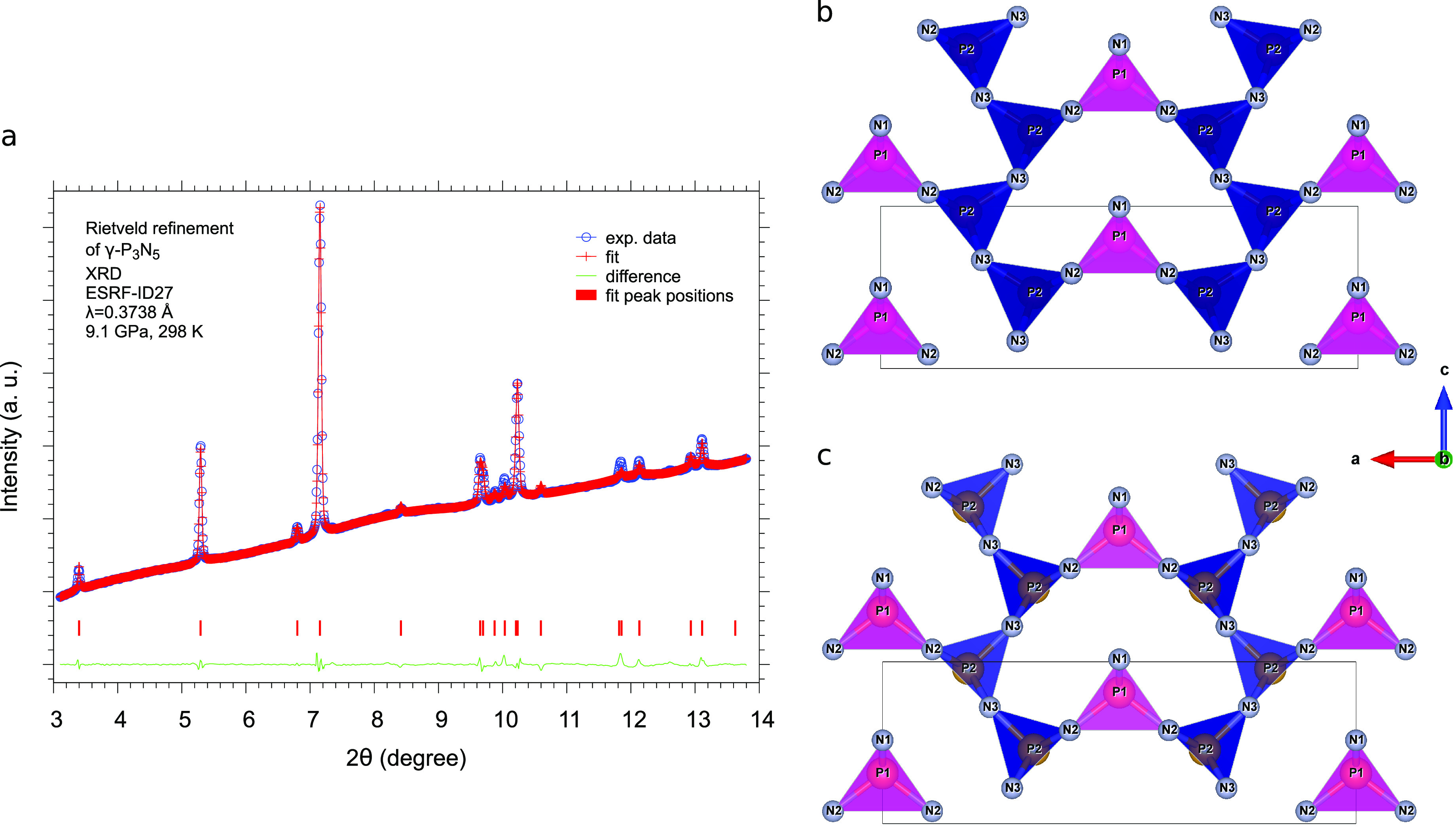
(a) Rietveld refinement of one of the XRD patterns assigned to γ-P_3_N_5_ acquired in the center of the laser-heated area at 9.1 GPa and room temperature after LH4. Views of the γ-P_3_N_5_ unit cell in the *ac* at plane at room temperature derived from XRD (b) at 9.1 GPa and (c) at ambient pressure.^50^ The PN_5_ square pyramids are highlighted in blue, and the PN_4_ tetrahedra, in magenta. The distortion due to pressure is highlighted in the *ac* plane by the position of the P2 atoms, slightly moving from the basis of the PN_5_ pyramids toward their internal volume, and of the P1 atoms, slightly moving away from the center of the PN_4_ tetrahedra.

The Rietveld refinement of the XRD pattern acquired in the center of the laser-heated area at 9.1 GPa and room temperature after LH4 is reported in Figure 5. All of the parameters relative to data collection and crystallographic data are reported in Table 1, and the refined atomic positions are provided in the cif file deposited at the Cambridge Crystallographic Data Centre with deposition number CSD 2164795.

**Table 1:** Crystallographic Data, Details of Data Collection, and Agreement Indices for the Final Least-Square Cycles of the Rietveld Refinement of γ-P_3_N_5_ at 9.1 GPa and Room Temperature

crystal system	orthorhombic
space group	*Imm*2
*a* (Å)	12.6041(7)
*b* (Å)	2.60103(15)
*c* (Å)	4.2740(2)
*V* (Å^3^)	140.12(1)
*Z*	2
wavelength (Å)	0.3738
2θ range (deg)	3.1–13.8
no. of observations	1300
parameters	46
*R*(*I*)	0.083
*R*_p_	0.004
*R*_wp_	0.007
*R*_exp_	0.056
goodness of fit	0.11

Our lattice parameters are in agreement with those recently published by Niwa et al.^27^ for γ-P_3_N_5_ recovered under ambient conditions after laser heating P and N_2_ at a slightly higher pressure of 12 GPa (*a* = 12.8905(7) Å, *b* = 2.6153(3) Å, and *c* = 4.4056(3)), even though the laser-heating temperature is not reported in their paper, thus making unclear whether, according to their laser-heating pressure, reactivity in their case occurred in liquid or in crystalline p-sc P.^6,7^

**Figure 6:**
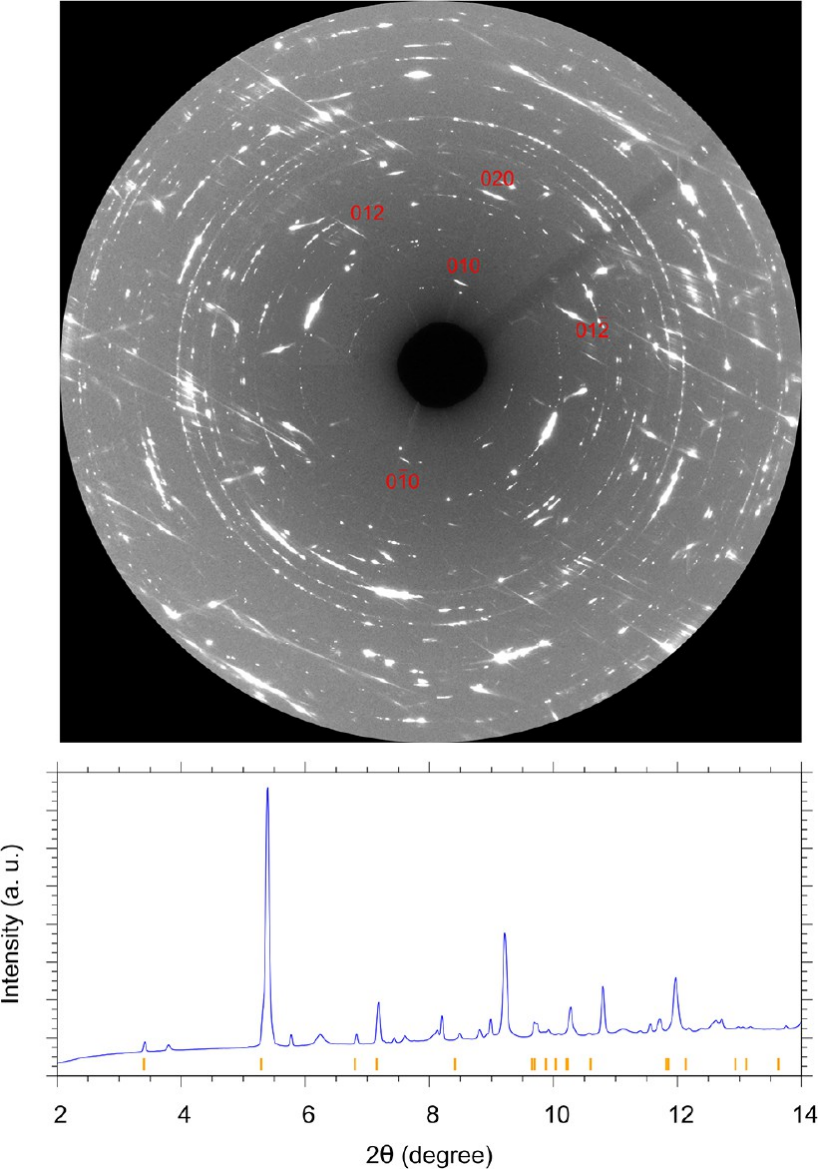
(Upper panel) Detector image showing a diffraction pattern acquired in the outer part of the laser-heated area (sample position b in Figure 3C) at 9.5 GPa and room temperature. Periodic intensity oscillations due to the corresponding lattice planes are labeled in red. The linear stripe aspect of the signal indicates a layer stacking disorder. (Lower panel) Azimuthally integrated diffraction pattern of the detector image shown in the upper panel (blue trace). The orange ticks represent the 2θ positions of the diffraction peaks of γ-P_3_N_5_ obtained by Rietveld refinement of a pattern acquired in the center of the laser-heated area (sample position a in Figure 3C) at 9.1 GPa. Besides the reflections assigned to α-P_3_N_5_ (*hkl* labels in the image) and γ-P_3_N_5_ (orange ticks in the integrated pattern), the other diffraction signals are due to unreacted P and N_2_, Au and ruby pressure markers, and Re from the gasket, in agreement with the sample position where the pattern was acquired and consistently with the Raman mapping shown in Figure 10.

The different diffraction signal detected in the outer portions of the laser-heated area revealed instead, among diffuse scattering, reflections compatible with a 0*kl* layer of the α-P_3_N_5_ phase (Figure 6). On the basis of the five most prominent reflections (010, 01̅0, 020, 012, and 012̅), two lattice parameters of α-P_3_N_5_ (*b* and *c*) out of four (*a*, *b*, *c*, and β) were determined as a function of pressure by fitting the individual reflections. Considering the pressure effect, the two interlayer distances of 5.65 and 8.85 Å at 9.5 GPa and room temperature are indeed compatible with the *b* and *c* lattice parameters reported by Horstmann et al.^47^ for the monoclinic (*Cc*) unit cell of α-P_3_N_5_ synthesized at ambient pressure by the high-temperature decomposition of [P(NH_2_)_4_]I at 825 °C (*a* = 8.12077(4) Å, *b* = 5.83433(4) Å, *c* = 9.16005(5) Å, β = 115.809(1)°, and *V*_0_ = 390.705 Å^3^). The characteristic aspect of this diffraction signal as linear stripes suggests the presence of a layer stacking disorder, as reported for β-P_3_N_5_.^48^ It is worth mentioning that the systematic absences in the indexed diffraction pattern do not match the *Cc* space group of bulk α-P_3_N_5_ likely because of the random stacking faults or different sheets stacking order.^48^ Although the spatial periodicities calculated for the two-dimensional layer actually correspond to the *b* and *c* lattice parameters of α-P_3_N_5_, this assumption has been adopted to explain the complex pattern observed in Figure 6.

### Characterization of the Reaction Products

Until very recently, only ambient-pressure structural XRD data and IR spectra were experimentally reported in the literature for α-P_3_N_5_ and γ-P_3_N_5_,^21,30,47^ with no characterization at high pressure. Together, the data presented here and those recently published by Niwa et al.^27^ provide a complementary XRD and Raman characterization of the structural and spectroscopic behavior of γ-P_3_N_5_, covering the entire pressure range of up to 50 GPa at room temperature.

#### X-ray Diffraction

**Figure 7:**
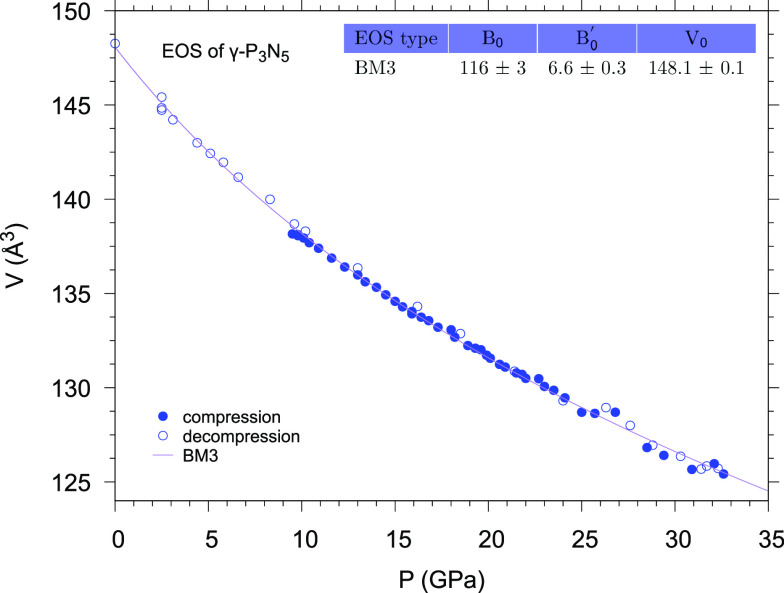
Pressure evolution of the unit cell volume of γ-P_3_N_5_ (*Imm*2) at room temperature. The solid circles refer to data acquired during the compression, and the empty circles refer to data acquired during the decompression. The data have been fitted according to the third-order Birch–Murnaghan equation of state (BM3).

After γ-P_3_N_5_ was synthesized, XRD patterns were acquired during room-temperature compression from 9.1 up to 32.6 GPa and further decompression down to ambient pressure (Figures SI-2 and SI-3), with the purpose of determining the equation of state of the material and exploring the existence of a phase transition to the predicted higher-pressure polymorphs.^21,22^ The peaks were fitted through the explored pressure range, and the lattice parameters and unit cell volume were derived from the orthorhombic geometrical relations. The values of the unit cell volumes as a function of pressure are plotted in Figure 7, where the full circles refer to data acquired during compression and empty circles refer to data acquired during decompression. The data were fitted using third-order Birch–Murnaghan- and Vinet-type equations of state (EOS). In all cases, the fits are very good and the obtained values of the bulk modulus (*B*_0_), its first derivative (*B*_0_′), and the unit cell volume at ambient pressure (*V*_0_) are consistent within the uncertainty (Figure SI-4). In particular, the *V*_0_ values obtained from the fit and measured at ambient pressure are in excellent agreement with the experimental value reported by Landkskron et al.^30^ Furthermore, our experimental *B*_0_ value (116 ± 3 GPa, see also Figure SI-4) perfectly matches the bulk modulus value obtained by DFT calculations using the LDA approximation (*B*_0_ = 116 GPa).^21^ The *B*_0_′ value (6.6 ± 0.3, see also Figure SI-4), while still being in good agreement, is instead slightly higher than the values predicted by computational studies.^21,22^ The discrepancy with the experimental *B*_0_ and *B*_0_′ values reported by Niwa et al.^27^ (*B*_0_ = 130.27 GPa, *B*_0_′ = 4 fixed value) is due to the fact that, whereas the XRD data by Niwa et al. contain only seven experimental data points in the 0–18 GPa pressure interval and a single additional calculated point at 50 GPa, our experimental data continuously cover the pressure range between 0 and 32.6 GPa, providing a significantly higher data sampling in terms of pressure resolution and consequently allowing the determination of the first derivative of the bulk modulus from the fitting procedure using a third-order Birch–Murnaghan equation. No hints of an additional phase transition is observed by XRD in this pressure range, in contrast to the predictions of DFT calculations based on the LDA approximation, which suggest the occurrence of the γ-P_3_N_5_ to δ-P_3_N_5_ phase transition above 25 GPa.^21^

**Figure 8:**
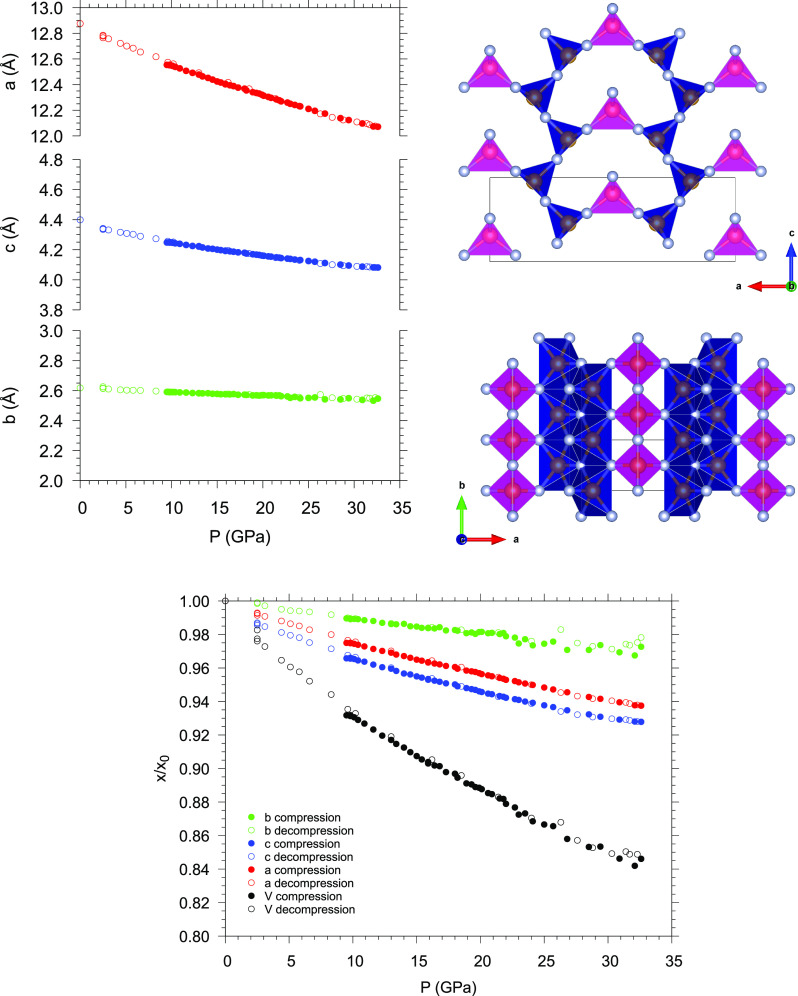
(Left panel) Pressure evolution of *a*, *b*, and *c* lattice parameters of the unit cell of γ-P_3_N_5_. (Right panels) Views of the γ-P_3_N_5_ structure along the *b* (top) and *c* (bottom) crystalline directions. (Lower panel) Room-temperature pressure evolution of the relative values of the *a*, *b*, and *c* lattice parameters and volume *V* of the γ-P_3_N_5_ unit cell with respect to their ambient pressure values (*a*_0_, *b*_0_, *c*_0_, and *V*_0_). The *x*/*x*_0_ label of the *y* axis indicates *a*/*a*_0_, *b*/*b*_0_, *c*/*c*_0_, and *V*/*V*_0_.

Another interesting piece of information emerging from our data concerns the anisotropic compressibility of γ-P_3_N_5_. As a matter of fact, the pressure evolution of the lattice parameters indicates that the compressibility of γ-P_3_N_5_ is significantly larger along the *a* and *c* directions than along the *b* direction, along which γ-P_3_N_5_ appears to be almost incompressible (Figure 8). This occurrence is consistent with the chemical bonding network of the material, which features channel-like cavities along the *b* direction, having a transverse bean-shaped section in the *ac* plane of relatively large diameter (Figure 8, right top panel).

Indeed, along the *a* and *c* directions the PN_4_ tetrahedra and the PN_5_ square pyramids are connected through one corner by one bridging N atom. The increase in density is thus accordingly expected to compress and distort the bean-shaped cavity in the *ac* plane.

Along the *b* direction, instead, the PN_5_ square pyramids are connected by one edge through two shared N atoms. The compression is thus prevented by strong directional chemical bonds, which make the material stiffer along this direction. The observed anisotropic compressibility of γ-P_3_N_5_ is in agreement with previous calculations^21^ and clearly emerges from the pressure evolution of the relative values of *a*, *b*, and *c* with respect to their ambient pressure values (Figure 8 lower panel). Also in this case the comparison with recent experimental data^27^ highlights the higher experimentally investigated pressure range and the higher number of pressure points in our data.

**Figure 9:**
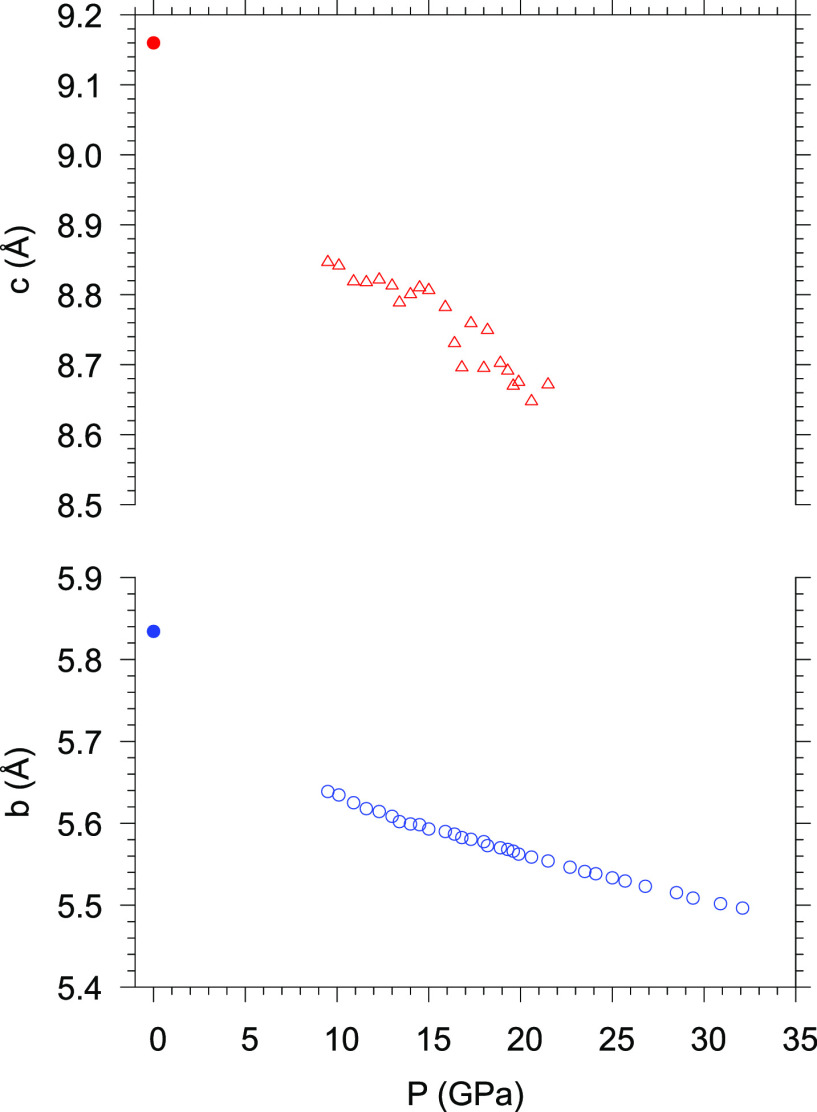
Room-temperature pressure evolution of the experimental *c* (red up triangles) and *b* (blue open circles) lattice parameters of α-P_3_N_5_. The filled symbols at ambient pressure are from ref (47).

Concerning α-P_3_N_5_, the pressure variation of the *c* parameter, despite some data scattering, exhibits a clear monotonous decrease with pressure on compression at up to ∼20 GPa. No determination of the *c* parameter was possible above this pressure. On the other hand, the pressure dependence of the *b* parameter is monotonous up to the highest investigated pressure of 32.6 GPa (Figure 9). Whereas for γ-P_3_N_5_ the *a*, *b*, and *c* lattice parameters could be determined as a function of pressure and the equation of state could be derived, for α-P_3_N_5_ only the pressure evolution of the *b* parameters could be determined up to the highest investigated pressure (Section SI-1, Figure SI-5).

The identification of the *b* lattice parameter at up to 32.6 GPa indicates that α-P_3_N_5_, once formed at 9.1 GPa and high temperature, remains metastable at room temperature up to this pressure, well beyond the predicted phase transition to γ-P_3_N_5_, located by different static calculations (0 K, zero-point contributions neglected) at ∼2.2 GPa (LDA) and 6.5 GPa (GGA).^21,22^ To the best of our knowledge, the pressure of 32.6 GPa observed here actually represents the highest experimental pressure at which α-P_3_N_5_ was reported to exist.^47^ Our own calculated pressure and temperature conditions for the α-P_3_N_5_/γ-P_3_N_5_ phase equilibria can be expressed in terms of the Clapeyron equation as follows: *P*_t_ = 5.1 + 0.00162*T*, where *P*_t_ is the transition pressure in GPa and *T* is the temperature in K. We obtain a positive slope for the α-P_3_N_5_ to γ-P_3_N_5_ transformation, as expected for a transition toward a denser and more ordered structure with lower entropy upon compression (Figure SI-6). In fact, the transition evolves from a monoclinic (*Z* = 4) to a higher-symmetric orthorhombic (*Z* = 2) unit cell with fewer degrees of freedom. The existence of a kinetic barrier, which is inherent to reconstructive transitions that retain the high-pressure phase under room conditions after decompression, hinders this transformation from being observed below 9.1 GPa if the temperature is not high enough.^53^ At this pressure, because of the positive Clapeyron slope, the calculated equation predicts the transformation at a temperature of as high as 2500 K, which is in agreement with our observations. Both factors explain the metastability of α-P_3_N_5_ emerging from our experiments and support the conclusion that α-P_3_N_5_, forming in the outer region of the laser-heated areas at 9.1 GPa likely experiences insufficiently high temperature for the transition to the stable γ-P_3_N_5_ structure to occur at this pressure.

#### Raman

**Figure 10:**
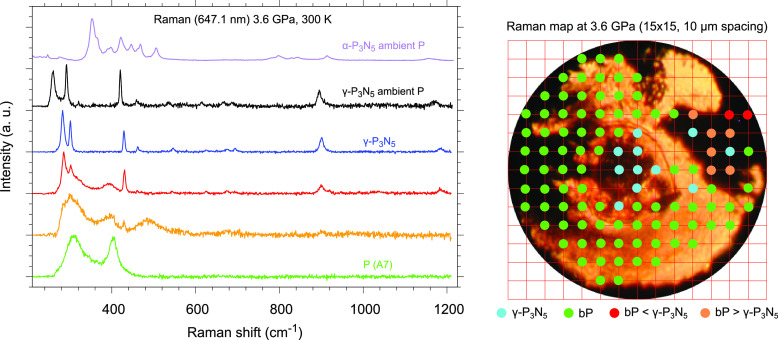
Raman spectra (left) acquired in the 200–1200 cm^–1^ frequency region during the mapping indicated by the red grid superimposed on the sample image (right). The colors of the spectra correspond to the colors of the points in the grid map: blue , γ-P_3_N_5_; green, P(A7); red, mixture of γ-P_3_N_5_ and P(A7); orange, P(A7) and α-P_3_N_5_. The purple spectrum refers to partially crystalline α-P_3_N_5_ at ambient pressure taken from ref (55). The XRD pattern related to the detector image in Figure 3B, acquired at the sample position b of Figure 3C, corresponds to one of the orange spectra in the Raman grid, where α-P_3_N_5_ was detected by XRD.

The synthesized sample was also studied with Raman spectroscopy at LENS. As in the case of XRD, after laser heating we performed a Raman mapping (15 × 15 grid, 10 μm spacing) of the reaction product in the 200–1200 cm^–1^ frequency region at 3.6 GPa and ambient pressure. The Raman mapping of the sample at 3.6 GPa is shown in Figure 10, where the colors of the spectra correspond to the colors of the points in the map. No contributions from the low-frequency phonon modes of N_2_ are expected in this frequency range at this pressure (Figure SI-9).^54^

The outcome of the Raman mapping is consistent with the X-ray data and indicates that, besides the ubiquitous presence of unreacted excess N_2_, revealed by the characteristic stretching band at 2341.5 cm^–1^ (N≡N), the spectra acquired outside the laser-heated area (green trace and green points in the grid of Figure 10) contain only rhombohedral P (A7), which is metastable at this pressure because of the hysteresis of the phase transition to the orthorhombic A17 structure.^14,56^ The spectra acquired inside the LH area (blue trace and blue points in the grid of Figure 10) instead contain new different sharp vibrational bands corresponding to γ-P_3_N_5_. These bands can also be observed on the recovered product under ambient conditions (black trace in Figure 10), and their frequencies correspond to those calculated by Kroll et al. for γ-P_3_N_5_.^21^

In the outer regions of the laser-heated area, an intermediate scenario occurs where both rhombohedral A7 P and γ-P_3_N_5_ are present. No clear spectroscopic evidence of α-P_3_N_5_ is instead observed in the sample regions where XRD suggests its presence, as indicated by the comparison between the Raman spectrum of the reaction product acquired in the sample position corresponding to the XRD pattern in Figure 3B (sample position b in Figure 3C) and the Raman spectrum of partially crystalline and amorphous α-P_3_N_5_ (Figure 10). This apparent inconsistency is, however, not surprising because the efficiency of Raman spectroscopy is drastically reduced when probing the inner parts of opaque samples, such as P or P_3_N_5_, compared to their surfaces, whereas XRD provides information on their bulk structure. In the outer regions of the laser-heated area, the laser intensity is lower because of the energy distribution of the beam profile. Accordingly, the temperature generated in these regions is expected to be lower than in the center of the laser-heated area. Furthermore, because the laser was focused on the sample based on the optical image of the sample surface, the surface of the sample may have experienced a higher temperature with respect to its interior. As a consequence, considering that α-P_3_N_5_ is known to convert to γ-P_3_N_5_ under sufficiently high pressure and temperature conditions,^30^ it is not unlikely that α-P_3_N_5_ may have formed only in the inner part of the sample in the peripheral regions of the laser-heated area, hence not being detectable in the Raman spectrum acquired in the sample positions where XRD revealed its presence. Nevertheless, comparing the Raman spectrum of partially crystalline α-P_3_N_5_ at ambient pressure with the Raman spectrum acquired in the grid positions corresponding to Figure 3B, it emerges that, whereas in the first one an intense band is present at 353 cm^–1^ and a structure consisting of four resolved peaks extends between 421 and 506 cm^–1^, in the second one a weak shoulder at 375 cm^–1^ (on the low-frequency side of the broad band belonging to rhombohedral A7 P at 403 cm^–1^) and a broad feature centered at 490 cm^–1^ can be appreciated. The band at 375 cm^–1^ and the broad feature centered at 490 cm^–1^ appear to be high-frequency shifted by 22 and 26.5 cm^–1^, respectively, compared to the Raman band at 353 cm^–1^ and to the center of the four-peak structure (463.5 cm^–1^) observed in the spectrum of partially crystalline α-P_3_N_5_ at ambient pressure. Considering the structural disorder observed in the XRD pattern shown in Figure 3B (acquired at sample position b in Figure 3C) and the pressure-broadening effect, a merging of the four bands observed between 421 and 506 cm^–1^ in the Raman spectrum of α-P_3_N_5_ (orange trace in Figure 10) could result in the observed Raman spectrum.

**Figure 11:**
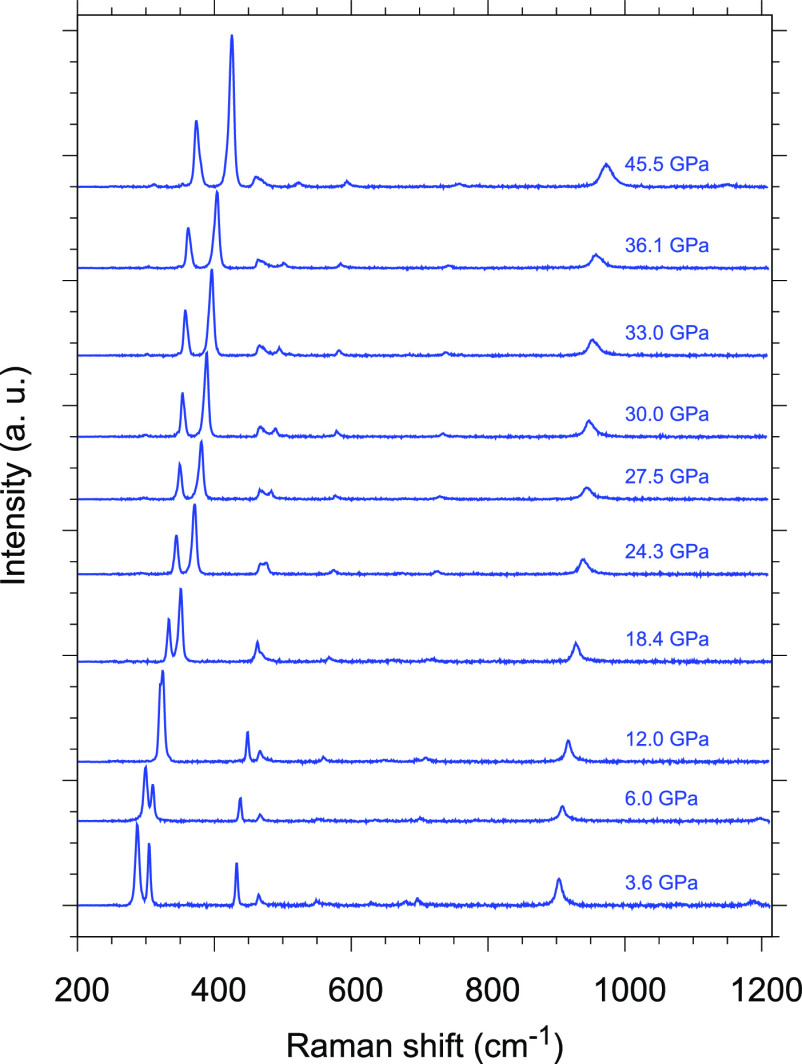
Raman spectra of γ-P_3_N_5_ acquired in the 200–1200 cm^–1^ frequency region during room-temperature compression from 3.6 up to 45.5 GPa.

**Figure 12:**
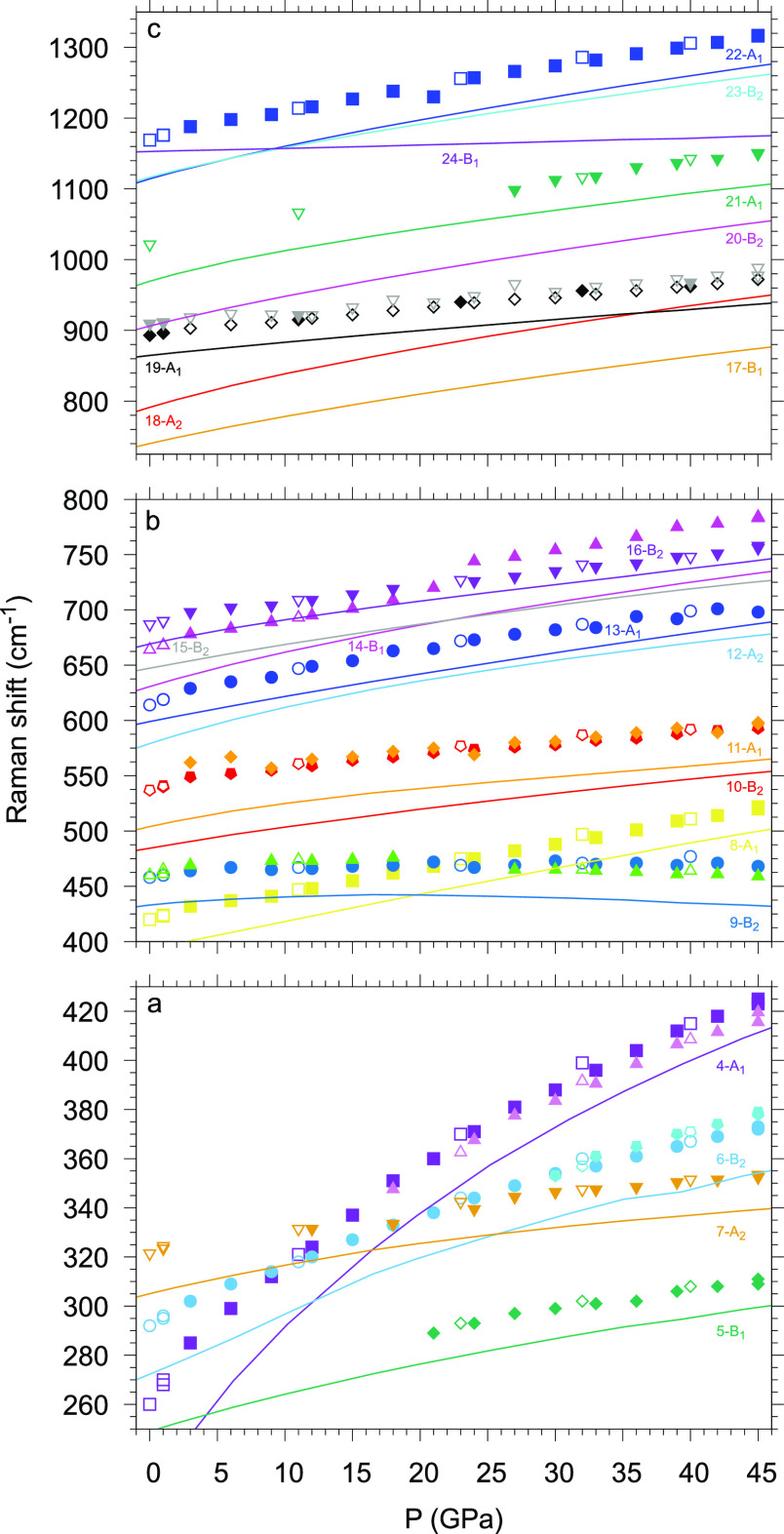
Experimental and calculated vibrational Raman frequencies of γ-P_3_N_5_. The experimental data are plotted as points, whereas the calculated frequencies of the 21 optical normal modes are plotted as lines and identified by the corresponding number of the mode-symmetry label. Among the experimental points, the solid symbols were acquired on compression and the empty symbols were acquired on decompression. The experimental and calculated data corresponding to the same normal mode are plotted with the same color when the assignment is clear. To facilitate the presentation, the data are divided into three frequency regions: (a) 250–430, (b) 400–800, and (c) 680–1380 cm^–1^.

Raman spectra were acquired as a function of pressure to gain insight into the vibrational properties of γ-P_3_N_5_ during room-temperature compression from 3.6 up to 45.5 GPa and further decompression under ambient conditions (Figures 11 and SI-7). The observed Raman frequencies were determined by fitting the spectrum with Voigt peak profiles and assigned to the corresponding vibrational modes by performing DFT calculations on γ-P_3_N_5_ at up to 102.6 GPa and by comparing the experimental frequency values with the calculated ones (Figures SI-8 and SI-9), considering the similar frequency evolution with pressure. A systematically slightly lower value is observed for the calculated frequencies with respect to the experimentally observed ones (Figure SI-9). Details of the frequency evolution with pressure are provided in Figures 12, where three different spectral windows (250–430, 400–800, and 680–1380 cm^–1^) spanning the entire investigated frequency range are shown (Figure SI-9). Any contribution of the N_2_ lattice modes to the fit of the experimental frequencies in the 220–430 frequency range has been checked and confidently excluded because the slope of the phonon frequencies across the crystalline structures of molecular N_2_ is markedly higher than the slope of the γ-P_3_N_5_ vibrational frequencies (Figure SI-9).^54^

**Figure 13:**
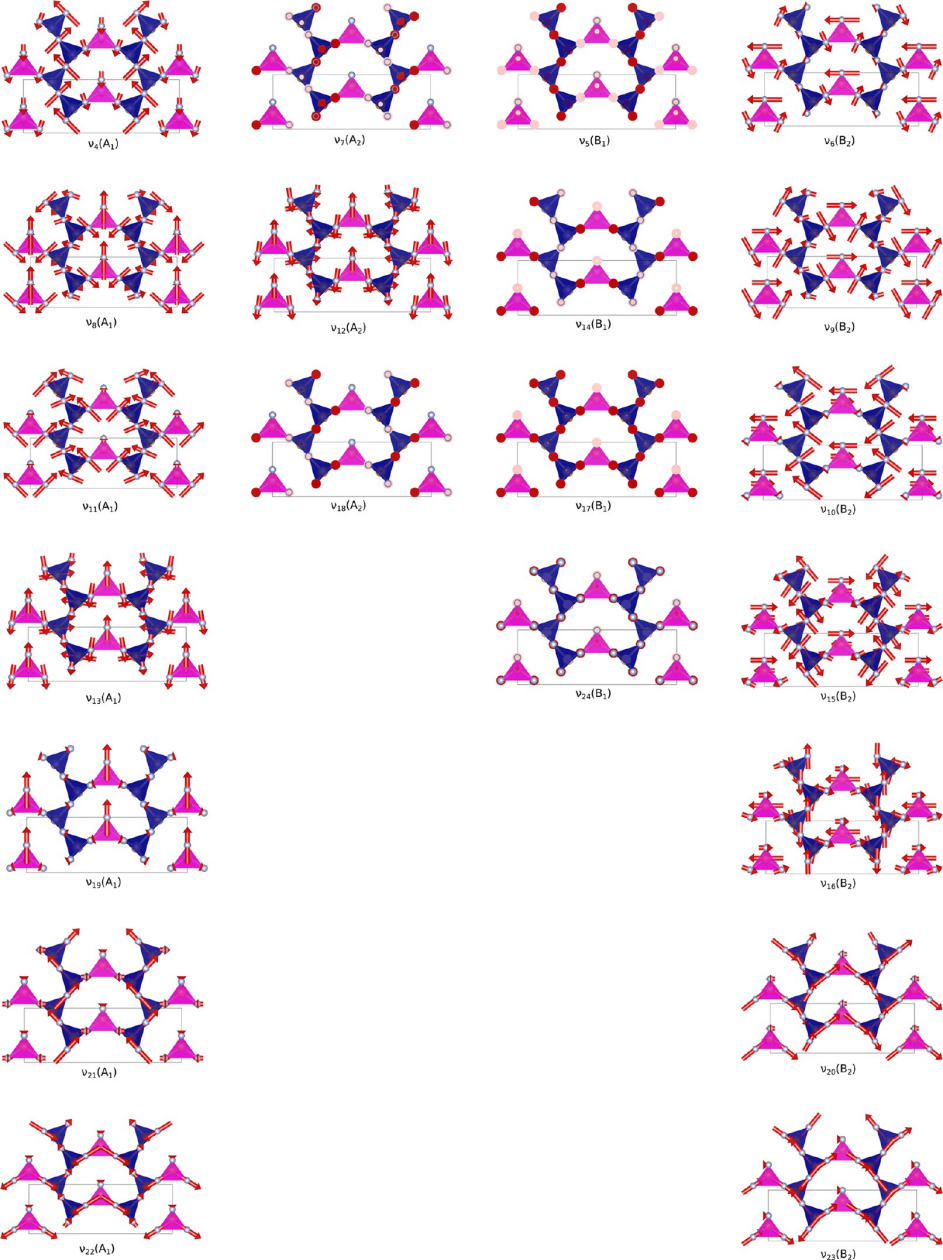
Calculated optical vibrational modes of γ-P_3_N_5_ (*i* = 4–24) identified by the corresponding vibrational frequency (ν_*i*_) at 0 GPa and static conditions (0 K and zero-point contributions neglected). The PN_5_ square pyramids are highlighted in blue, and the PN_4_ tetrahedra are highlighted in magenta. The direction and intensity of the atomic displacements are indicated by the red arrows. Each column lists the modes belonging to the same symmetry (left to right: A_1_, A_2_, B_1_, and B_2_).

According to the factor group analysis, because of the presence of one P_3_N_5_ formula unit in the primitive cell, no Davydov crystal field splitting is expected in the *Imm*2 unit cell and the irreducible representations of the 21 optical normal vibrational modes associated with the P_3_N_5_ unit are 7A_1_, 3A_2_, 4B_1_, and 7B_2_. Within the numbering adopted in this article, modes 1–3 correspond to acoustic modes and modes 4–24 correspond to optical modes. Optical modes 4–7 are associated with polyhedral rotations, modes 8–19 correspond to bending modes, and modes 20–23 correspond to P–N stretching modes, whereas mode 24 is a bending mode involving free tetrahedral N atoms (Figure 13).

Except for a slight underestimation, the calculated Raman frequencies are in substantial agreement with the experimental ones.

Within the set of 21 expected frequencies associated with the optically active Raman modes, ν_12_(A_2_), ν_15_(B_2_), ν_17_(B_1_), ν_18_(A_2_), ν_20_(B_2_), ν_23_(B_2_), and ν_24_(B_1_) could not be appreciated in the experimental spectra, likely because of their low intensity, pressure broadening, and frequency overlap with other bands. The experimental frequencies assigned to the calculated ν_4_(A_1_) and ν_6_(B_2_) vibrational modes exhibit a second component appearing as weak shoulders during compression at 18 and 30 GPa and disappearing on decompression at 23 and 32 GPa, respectively. A weaker component also accompanies the experimental frequency assigned to the calculated ν_9_(B_2_) mode across the whole pressure range during compression and decompression. The experimental frequencies corresponding to the calculated ν_5_(B_1_) and ν_21_(A_1_) modes become observable during compression at 23 and 27 GPa and are detected on decompression until 21 GPa and ambient pressure, respectively. Considering the small frequency difference with respect to the main component and the absence of Davydov crystal field splitting, the weak shoulders observed for the ν_4_(A_1_), ν_6_(B_2_), and ν_9_(B_2_) experimental frequencies are likely related to the occurrence of LO–TO splitting.

**Figure 14:**
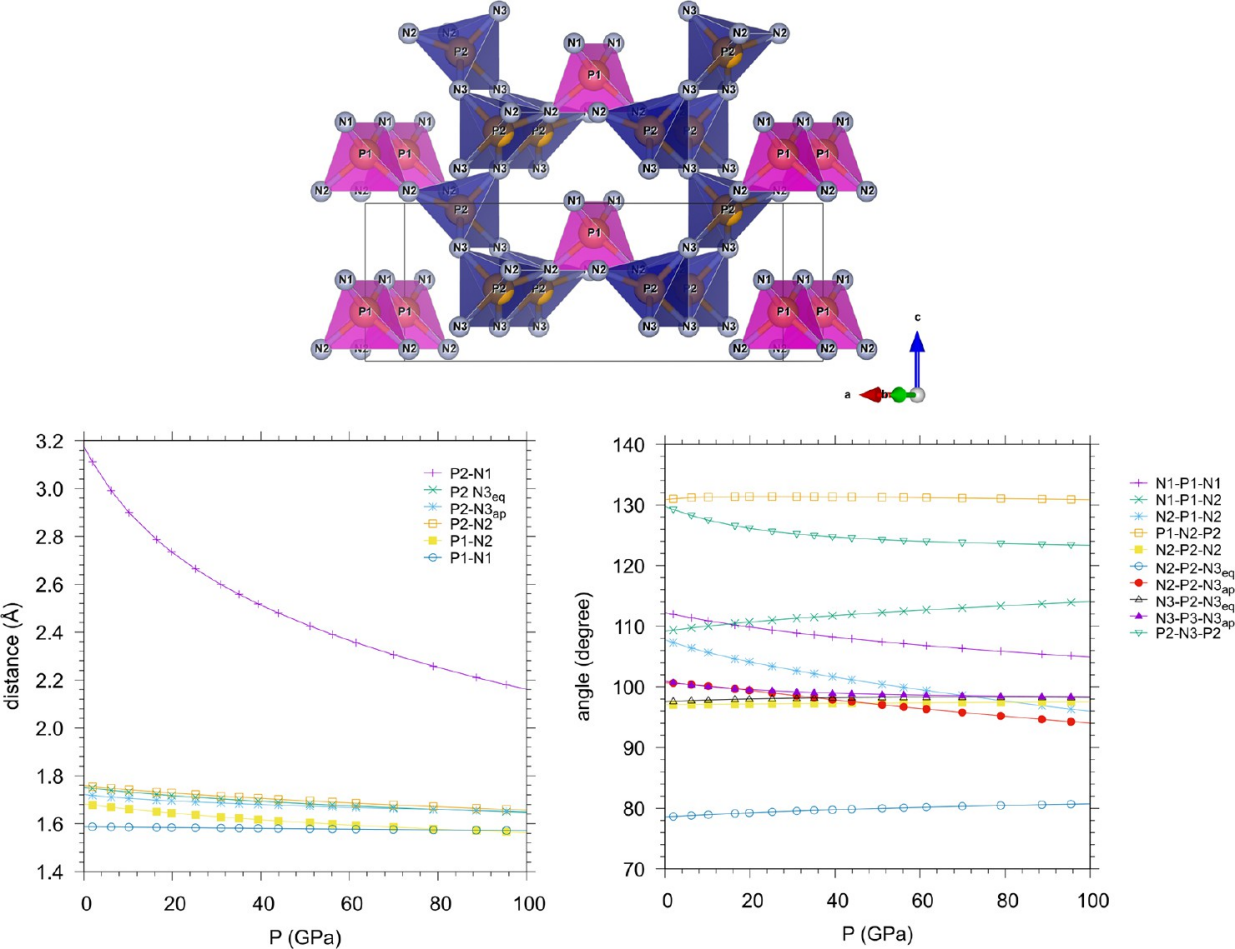
Calculated evolution with pressure of distances and angles in γ-P_3_N_5_. As shown in the upper image, P1 corresponds to the PN_4_ tetrahedra, whereas P2 corresponds to square-pyramidal PN_5_. Subscripts ap and eq in the atom labels of the legend indicate apical and equatorial postions, respectively, of the N3 atoms in the square-pyramid polyhedra.

Whereas most of the Raman bands exhibit similar behavior with increasing frequency on compression and decreasing frequency on decompression, three of them show a characteristic evolution. In particular, the frequency of the ν_9_(B_2_) mode (Figure 12b and Figure SI-9), initially increasing with pressure, reaches a maximum at 16.7 GPa and then starts to decrease up to the highest investigated pressure of 45.5 GPa. Like the other B_2_ modes, ν_9_ involves the rotation of the polyhedra in the *ac* plane, but this particular mode has the largest amplitude of motion for the N atoms belonging to the PN_4_ units. The nonmonotonic evolution with pressure of the ν_9_(B_2_) frequency indicates a softening of the mode above 16.7 GPa, likely related to the deformation of the cavity in the *ac* plane. This occurrence is consistent with the evolution with pressure of the calculated value of the P1–N2–P2 angle, showing a maximum (131.4°) at 25 GPa, indicating that this vibrational mode becomes stiffer for larger angle values (Figure 14).

The calculated frequency of the ν_24_(B_1_) mode (Figure 12c and Figure SI-9), not observed experimentally, increases much less than all of the other frequencies, with only a 25 cm^–1^ high-frequency shift (from 1150 to 1175 cm^–1^) in the 0–45 GPa pressure range. ν_24_ mostly involves the displacement of the P and N atoms along the *b* direction, with the largest amplitude of motion for the N atoms forming the PN_4_ tetrahedra in the center of the cavity, which are likely less affected by the compression.

Finally, the ν_4_(A_1_) frequency exhibits the highest increase with pressure among all of the other modes, crossing the pressure evolution of the vibrational frequency corresponding to ν_6_(B_2_) at ∼10.5 GPa (Figure 12a and Figure SI-9). ν_4_ involves out-of-phase motion of the PN_4_ tetrahedra and PN_5_ square pyramids, leading to the displacement of the PN_4_ units toward the center of the cavity, which is likely expected to become harder with pressure as a result of the shrinkage of the cavity in the *ac* plane. The frequency of ν_4_ is indeed observed to cross those of ν_6_(B_2_) and ν_7_(A_2_) at 10.5 GPa. The behavior of the ν_4_ frequency with pressure is mirrored by the evolution of the N2–P1–N2 angle, which exhibits the largest variation with pressure among the other angles, decreasing by 11.8° from ambient pressure to 100 GPa (Figure 14). The compression of γ-P_3_N_5_ appears to occur mainly through the rotation of the PN_5_ square pyramids (P2–N3–P2 angle) and the deformation of the PN_4_ tetrahedra (N2–P1–N2 angle) (Figure 14).

These observations are in agreement with the anisotropic compressibility of the γ-P_3_N_5_ unit cell emerging from the pressure evolution of the lattice parameters (Figures 8), consistently with the observation that along the *b* direction the PN_5_ square pyramids interact through two shared edges with two strong directional covalent bonds on opposite sides, whereas in the *ac* plane the PN_4_ tetrahedra and the PN_5_ square pyramids are connected only through vertices. The atomic displacements occurring during compression can also be appreciated by comparing the experimental structures derived from XRD at ambient pressure^47^ and at 9.1 GPa (Figure 5).

#### Denser, Higher P Coordination Polymorphs

Phosphorus nitride polymorphs containing PN_6_ octahedral units, hence featuring higher octahedral P coordination by the N atoms with respect to α-P_3_N_5_ and γ-P_3_N_5_, have been predicted by calculations to exist on further compression. Among them, the kyanite type δ-P_3_N_5_^21^ and the V_3_O_5_-like P_3_N_5_^22^ structures maintain the P_3_N_5_ stoichiometry of the two experimentally synthesized lower-pressure polymorphs. PN_6_ octahedra have been predicted by calculations to exist at high pressure also for other compositional spaces of the P/N system,^23^ but they have been experimentally observed recently only in ternary β-BP_3_N_6_^24^ and BeP_2_N_4_,^57^ where B and Be, respectively, are also present.

No phase transition from γ-P_3_N_5_ to another structure was observed up to the highest investigated pressure in our experiments (XRD 32.6 GPa, Raman 45.5 GPa), with no discontinuities in the pressure evolution of the unit cell and lattice parameters obtained from XRD or in the pressure evolution of the Raman frequencies. Consistently, no band splitting was observed in the Raman spectra, and the appearance of a new Raman band during compression is interpreted with an intensity gain of expected bands rather than with the activation of new components. A possible structural path toward the formation of PN_6_ octahedra in the γ-P_3_N_5_ structure, involving the reciprocal approach of one of the N atoms (N1) at the vertex of a PN_4_ tetrahedron to the P atom (P2) at the base of a PN_5_ square pyramid and likely proceeding through the interaction of the N1 electron lone pair with the P2 atom, can be excluded in the investigated pressure range. Our calculations indicate that the distance between the P1 and N2 atoms remains significantly higher than the typical bonding distances between P and N atoms even at 100 GPa (Figure 14). This is particularly true in the pressure range where the γ-P_3_N_5_ to δ-P_3_N_5_ and the γ-P_3_N_5_ to V_3_O_5_-like P_3_N_5_ phase transitions have been predicted to occur by Kroll et al. (43 GPa using GGA and 28 GPa using LDA approximations)^21^ and by Dong et al. (35.5 GPa), respectively.^22^ Our findings are in agreement with the observation by Niwa et al.^27^ in the corresponding pressure range.

## Conclusions

Using LH in DAC, we successfully induced direct chemical reactivity between P and N under high-pressure and high-temperature conditions. The results presented in this study provide relevant insights about the synthesis of α-P_3_N_5_ and γ-P_3_N_5_ from the elements and their characterization at high pressure.

First, the identification of defined threshold values of pressure (9.1 GPa) and temperature (2000–2500 K), for the reaction to occur, indicates that chemical reactivity between the two lowest-Z pnictogens likely takes place in the liquid phase. This occurrence highlights the stability of the A7 layered structure of P at high temperature and suggests the P reactivity to benefit from the lower kinetic barrier as a result of the higher mobility of the atoms in the liquid phase of P.

Second, the reaction proceeds with the complete consumption of P, essentially leading to the formation of polycrystalline γ-P_3_N_5_ in the center of the laser-heated area and of metastable α-P_3_N_5_ in the outer portion of the laser-heated area. To the best of our knowledge, the formation of α-P_3_N_5_ from the elements has never been reported before because α-P_3_N_5_ is reported in the literature to be obtained only when starting from suitable precursors.^47,48^

Third, the detection of α-P_3_N_5_, in the outer regions of the laser-heated area, where the temperature during laser heating is expected to be lower, suggests the formation of α-P_3_N_5_ to be a preliminary step toward the synthesis of γ-P_3_N_5_, supporting the finding of Landskron et al. in agreement with the pressure-coordination rule.^30^ The *b* and *c* lattice parameters of α-P_3_N_5_ could be observed on compression by XRD diffraction up to 32.6 GPa and ∼20 GPa, respectively.

Fourth, γ-P_3_N_5_ was further characterized by synchrotron XRD diffraction, Raman spectroscopy, and DFT calculations, precisely determining the EOS and correlating the pressure effect on the structural parameters to that on the vibrational lattice dynamics. The experimental XRD and Raman data, supported by the calculated atomic distances and bond angles, indicate that the compression of γ-P_3_N_5_ essentially proceeds through the rotation of the PN_5_ square pyramids and the distortion of the PN_4_ tetrahedra.

Finally, no phase transition of γ-P_3_N_5_ to a structure with higher P coordination by the N atoms was observed in our experiments (up to 45.5 GPa), excluding the pressure-induced approach of N1 to P2 as a potential driving structural path toward the obtainment of PN_6_ octahedra.

## Supporting Information Available

Experimental details, additional data, and comments (PDF)
Calculated vibrational modes of γ-P_3_N_5_ (ZIP)
