# Supplementary material for: High-Pressure and High-Temperature Chemistry of Phosphorus and Nitrogen: Synthesis and Characterization of α- and γ-P3N5
Source: Inorg Chem. 2022 Jul 26;61(31):12165–80. doi: 10.1021/acs.inorgchem.2c01190 (PMC9374155; doi:10.1021/acs.inorgchem.2c01190)
Supplement: Supplementary file 1 — ic2c01190_si_001.pdf [file ic2c01190_si_001.pdf]

Supporting Information: High pressure and high temperature chemistry of phosphorus and nitrogen: synthesis and characterization of α- and γ-P_3N_5

Matteo Ceppatelli,[*,†,‡] Demetrio Scelta,[†,‡] Manuel Serrano-Ruiz,[‡] Kamil Dziubek,[†] Fernando Izquierdo-Ruiz,[¶,§] J. Manuel Recio,[¶] Gaston Garbarino,[‖] Volodymyr Svitlyk,[‖] Mohamed Mezouar,[‖] Maurizio Peruzzini,[‡] and Roberto Bini[†,⊥,‡]

†*LENS, European Laboratory for Non-linear Spectroscopy, Via N. Carrara 1, I-50019 Sesto Fiorentino, Firenze, Italy*

‡*ICCOM-CNR, Institute of Chemistry of OrganoMetallic Compounds, National Research Council of Italy, Via Madonna del Piano 10, I-50019 Sesto Fiorentino, Firenze, Italy*

¶*Malta-Consolider Team and Departamento de Química Física y Analítica, Universidad de Oviedo, Avda. Julián Clavería, 8. 33006 - Oviedo, España*

§*Department of Chemistry and Chemical Engineering, Chalmers University of Technology,Gothenburg 412 96 Sweden*

‖*ESRF, European Synchrotron Radiation Facility, 71 Avenue des Martyrs, CS40220, 38043 Grenoble Cedex 9, France*

⊥*Dipartimento di Chimica "Ugo Schiff" dell'Università degli Studi di Firenze, Via della Lastruccia 3, I-50019 Sesto Fiorentino, Firenze, Italy*

E-mail: ceppa@lens.unifi.it,matteo.ceppatelli@iccom.cnr.it

SI-1: XRD

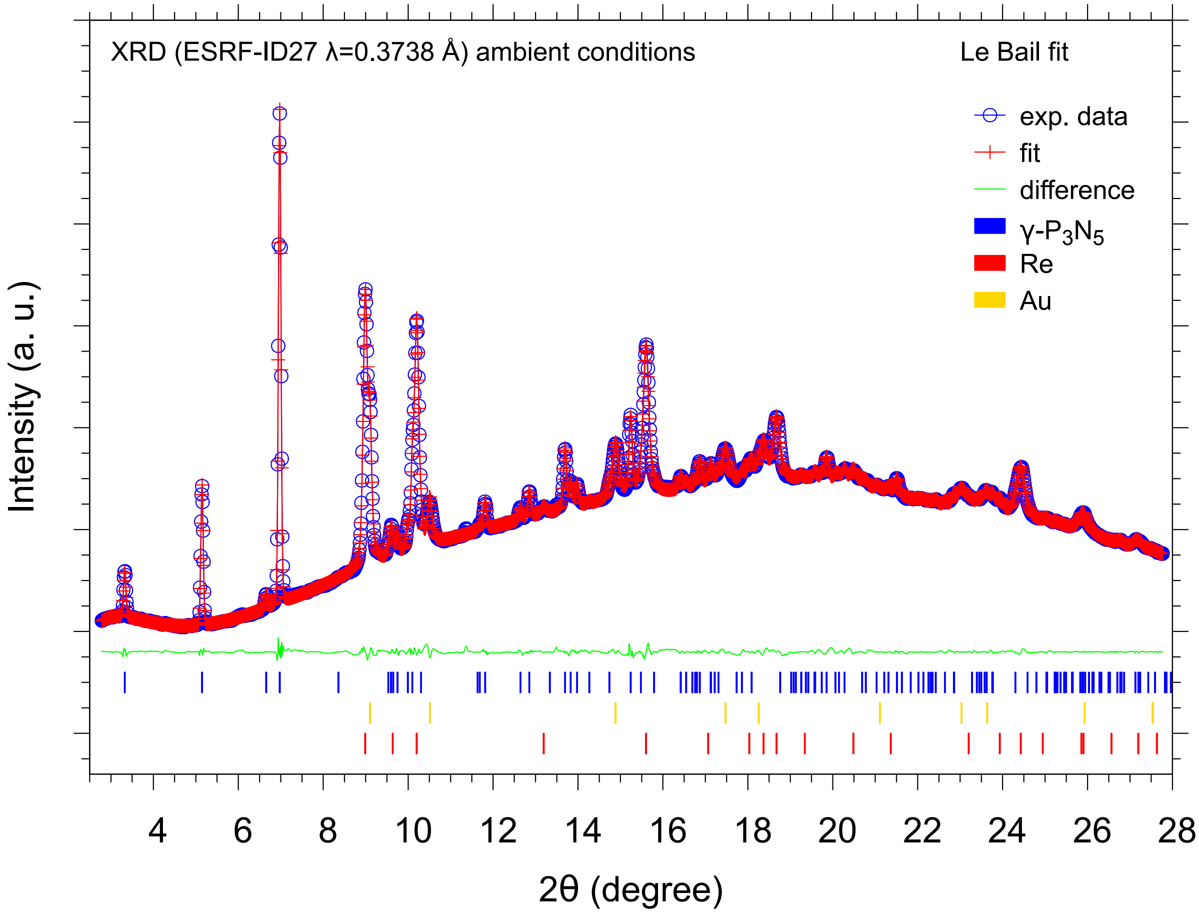

Figure SI-1: Multiphase Le Bail fit of the XRD pattern of the recovered sample at ambient conditions. The blue trace (empty blue circles and line), corresponding to the experimental data, has been obtained by adding several patterns acquired in different sample positions. The red trace represents the Le Bail fit of the experimental data, whereas the green trace represent the difference between the experimental and calculated intensity. The fit peak positions of the γ-P_3N_5 (blue), Au (gold) and Re (red) structures are also displayed as vertical bars. The determined lattice parameters and unit cell volume of γ-P_3N_5 are a=12.8757(5) Å, b=2.61745(15) Å, c=4.39920(15) Å, V_0= 148.259(8) Å^3. The Le Bail fit was performed using the JANA2006 software.[S1]

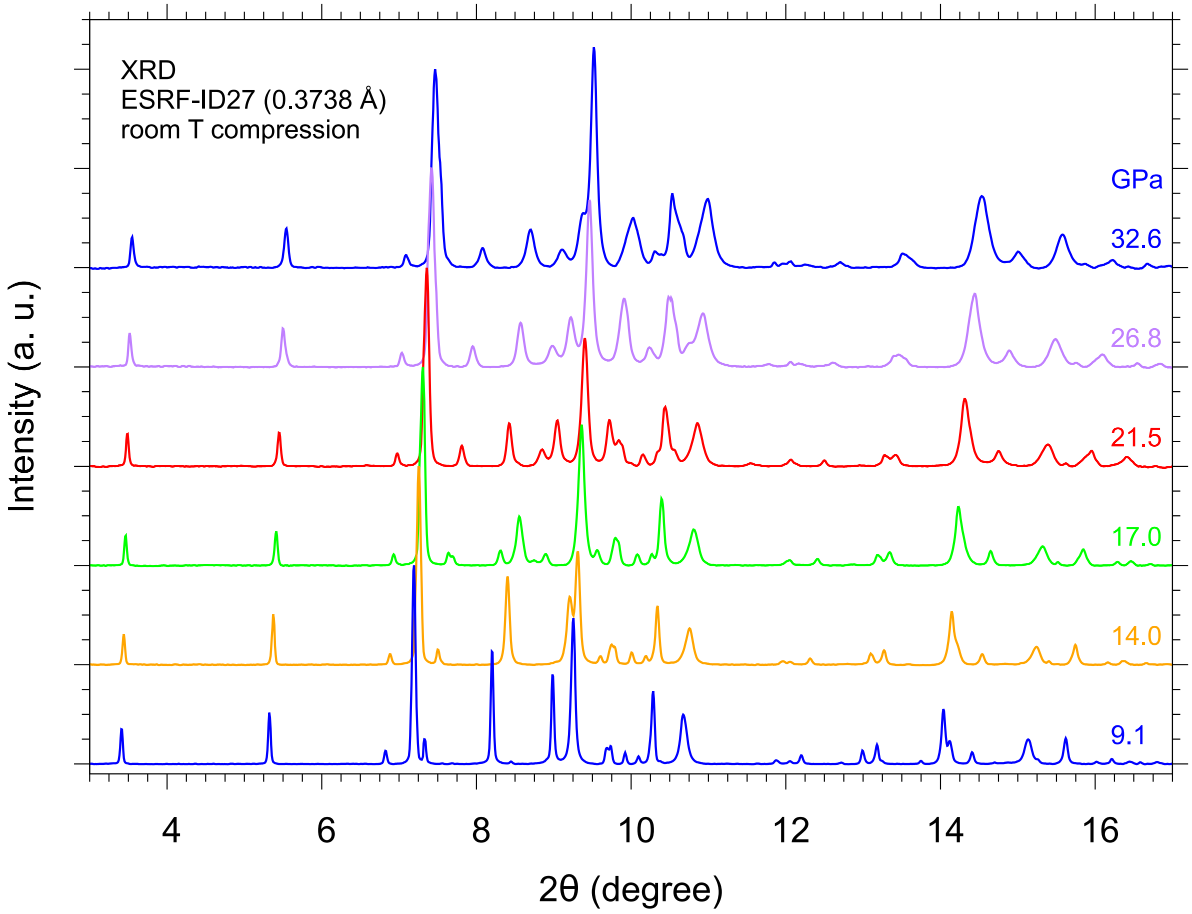

Figure SI-2: Integrated XRD patterns acquired during room T compression on a sample spot where γ-P_3N_5 was detected. Diffraction peaks from $\delta{-}N_2$, $\epsilon{-}N_2$ and Au (pressure sensor) are also present.

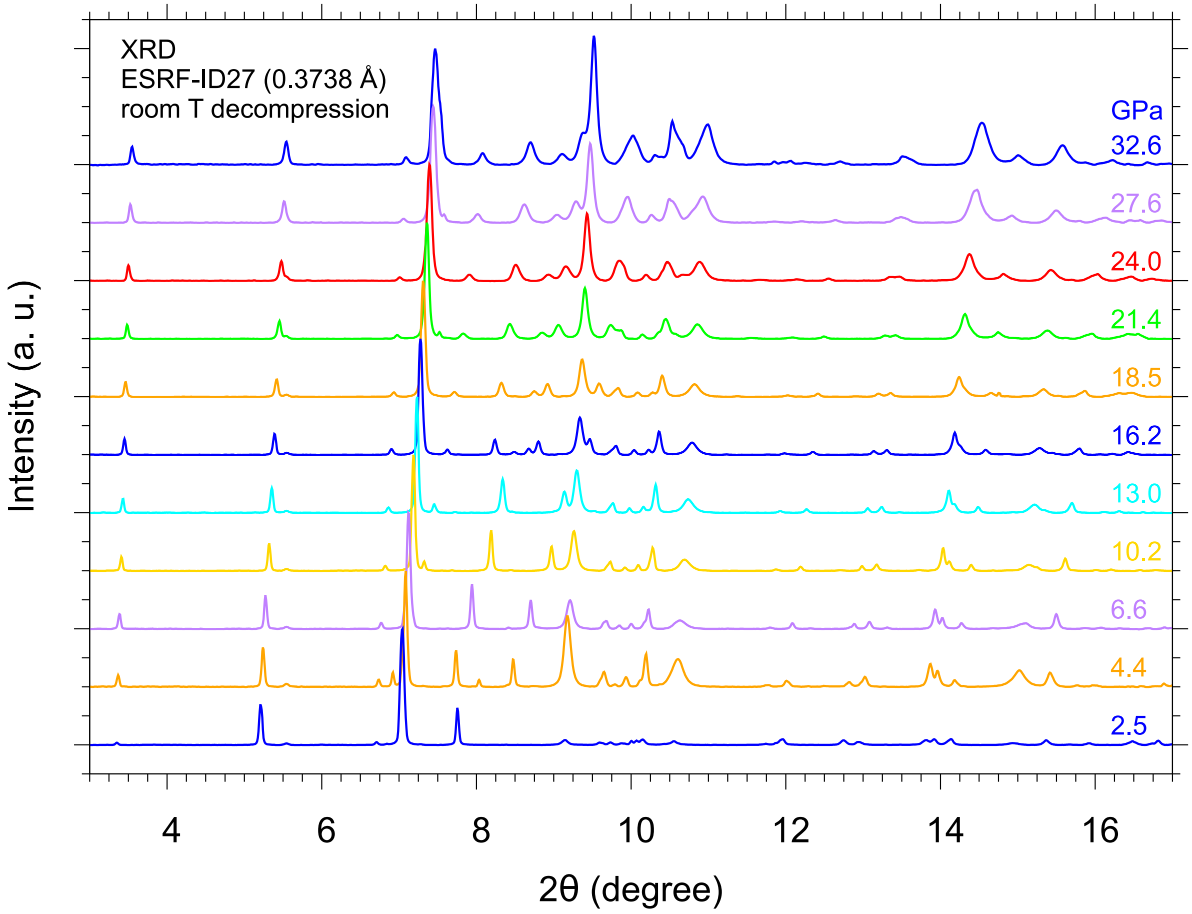

Figure SI-3: Integrated XRD patterns during acquired room T decompression on a sample spot where γ-P_3N_5 was detected. Diffraction peaks from $\delta{-}N_2$, $\epsilon{-}N_2$ and Au (pressure sensor) are also present.

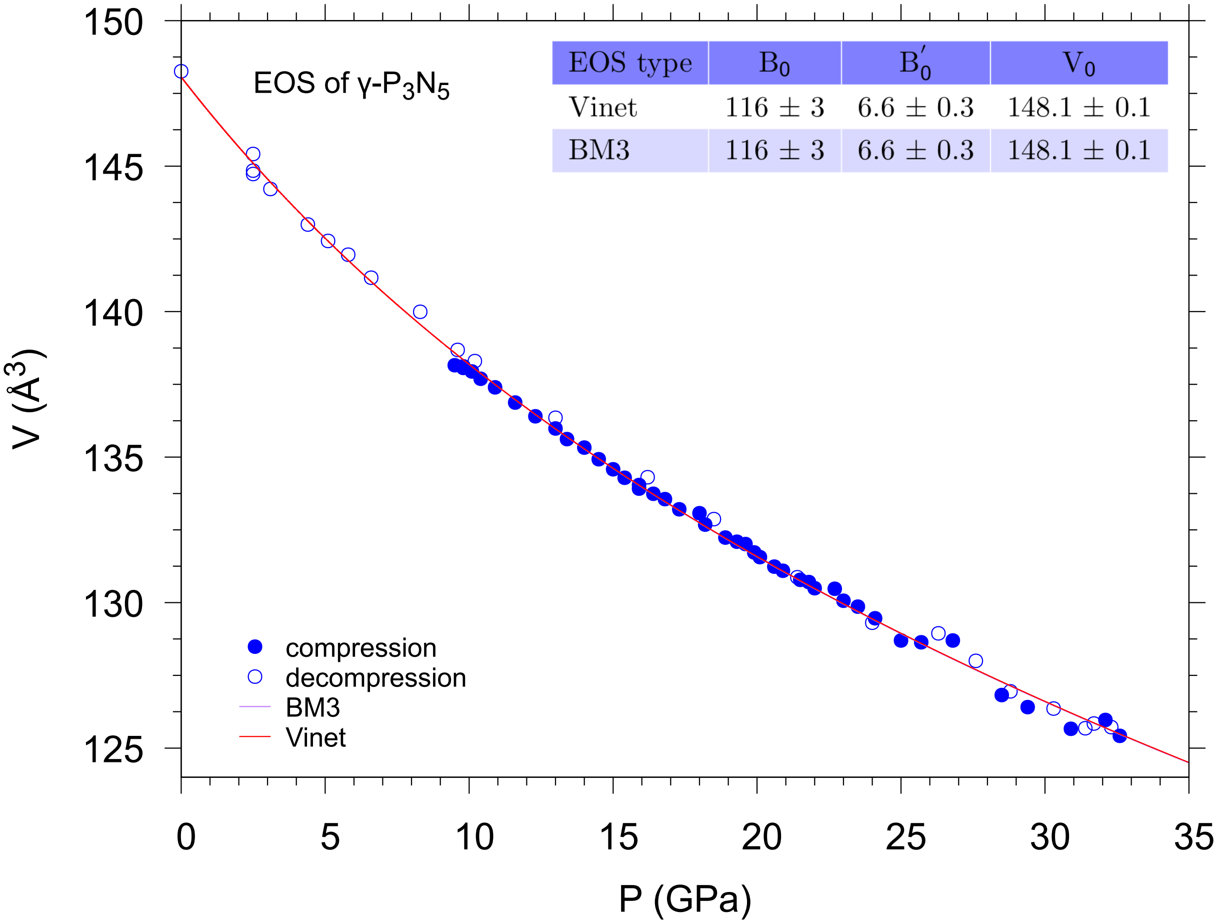

Figure SI-4: Pressure evolution of the unit cell volume of γ-P_3N_5 ($Imm2$) at room T. The full circles refer to data acquired during the compression and the empty circles to data acquired during the decompression. The data have been fitted according to Vinet and 3rd order Birch-Murnaghan equation of state considering the ambient pressure volume as a free fit parameter. The two fitting equations are in excellent agreement and can not be distinguished in the figure due to their overlap.

Whereas for γ-P_3N_5 the a, b and c lattice parameters could be determined as a function of pressure and the equation of state derived, for α-P_3N_5 only the pressure evolution of the b parameters could be determined up to the highest investigated pressure. Its linear modulus ($M_{b,0}$=49(6) GPa) and first derivative ($M_b^{'}$=226(31)) can be determined by fitting the evolution of the b values as a function pressure according to the following EOS,[S2–S4] knowing the value of the b parameter at atmospheric pressure (b_0) from literature:[S5]

$$P = \frac{3}{2}\left(\frac{M_{b,0}}{3}\right)\left[\left(\left(\frac{b}{b_0}\right)^3\right)^{-\frac{7}{3}} - \left(\left(\frac{b}{b_0}\right)^3\right)^{-\frac{5}{3}}\right]\left\{1 + \frac{3}{4}\left(\frac{M_b{}'}{3} - 4\right)\left[\left(\left(\frac{b}{b_0}\right)^3\right)^{-\frac{2}{3}} - 1\right]\right\} \quad (1)$$

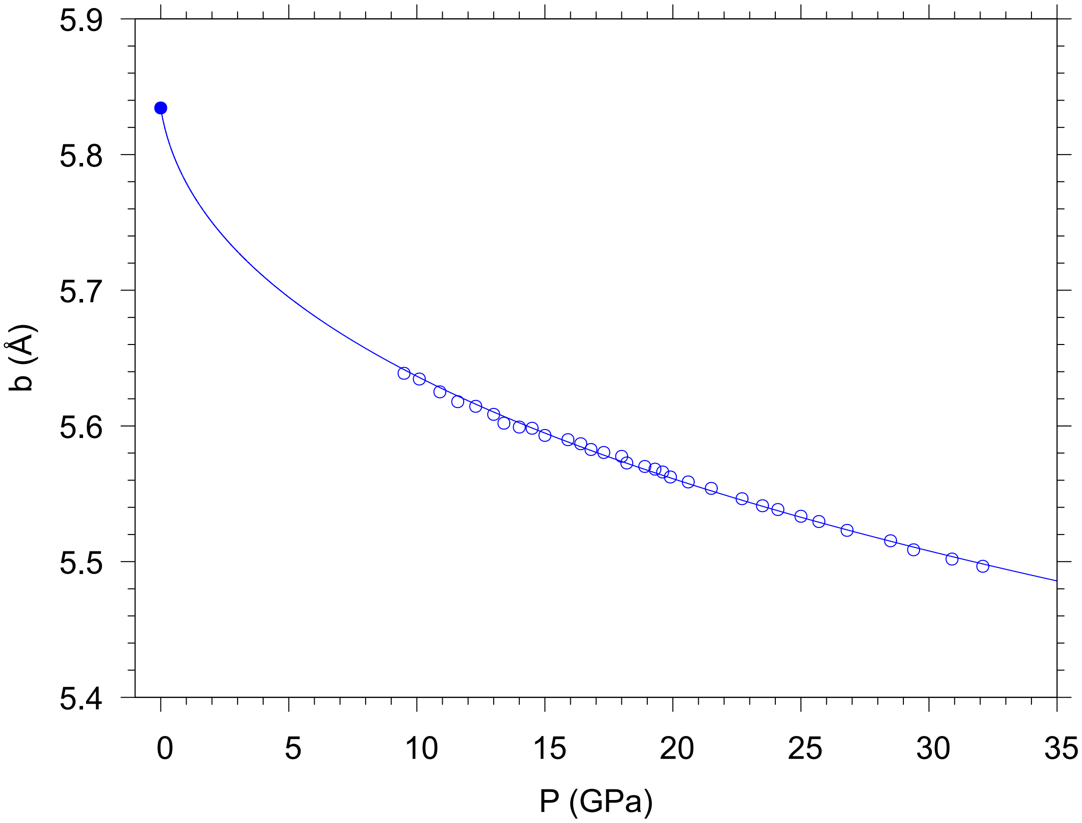

Figure SI-5: Room T pressure evolution of the experimental b (blue empty circles) lattice parameters of α-P_3N_5. The filled symbol at ambient pressure is from ref.[S6] The blue curve is obtained by fitting the data using the equation 1.

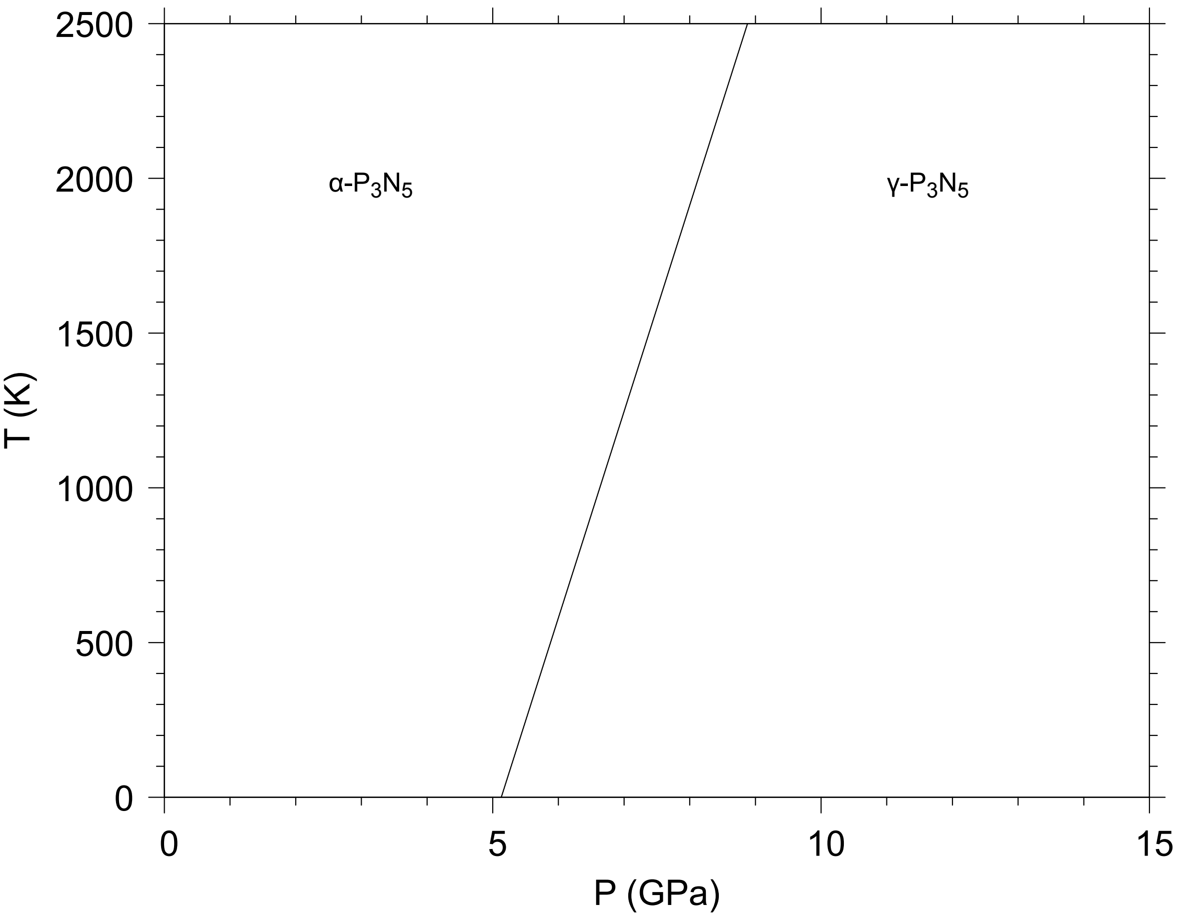

Figure SI-6: Calculated equilibrium pressure as a function of temperature (P_t = 5.1 + 0.00162 T) for the α-P_3N_5 to γ-P_3N_5 phase transition.

SI-2: Raman spectra

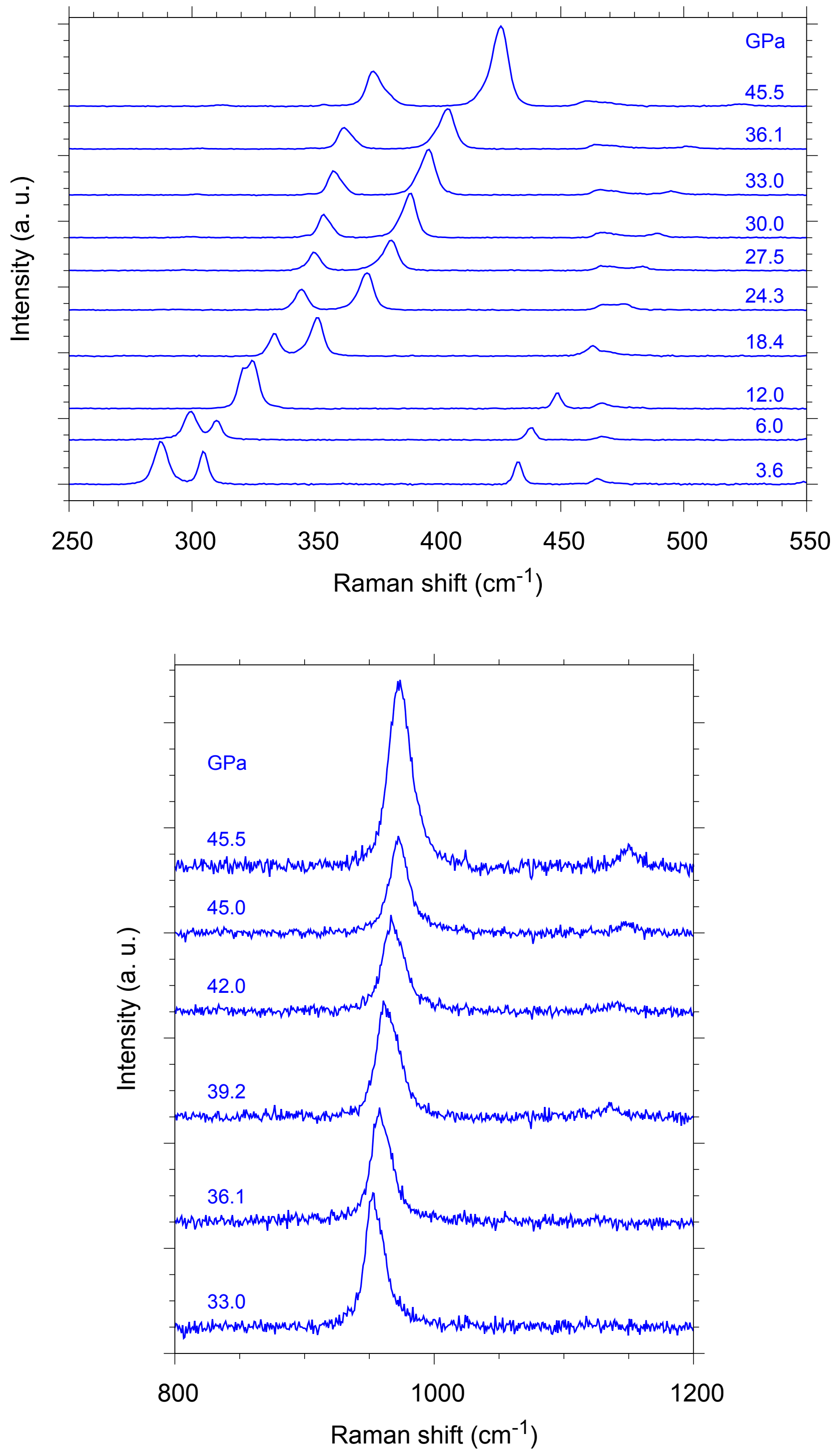

Figure SI-7: Raman spectra of γ-P_3N_5 as a function of pressure acquired during compression in the 250-550 cm^{-1} (upper panel) and 800-1200 cm^{-1} (lower panel) frequency regions.

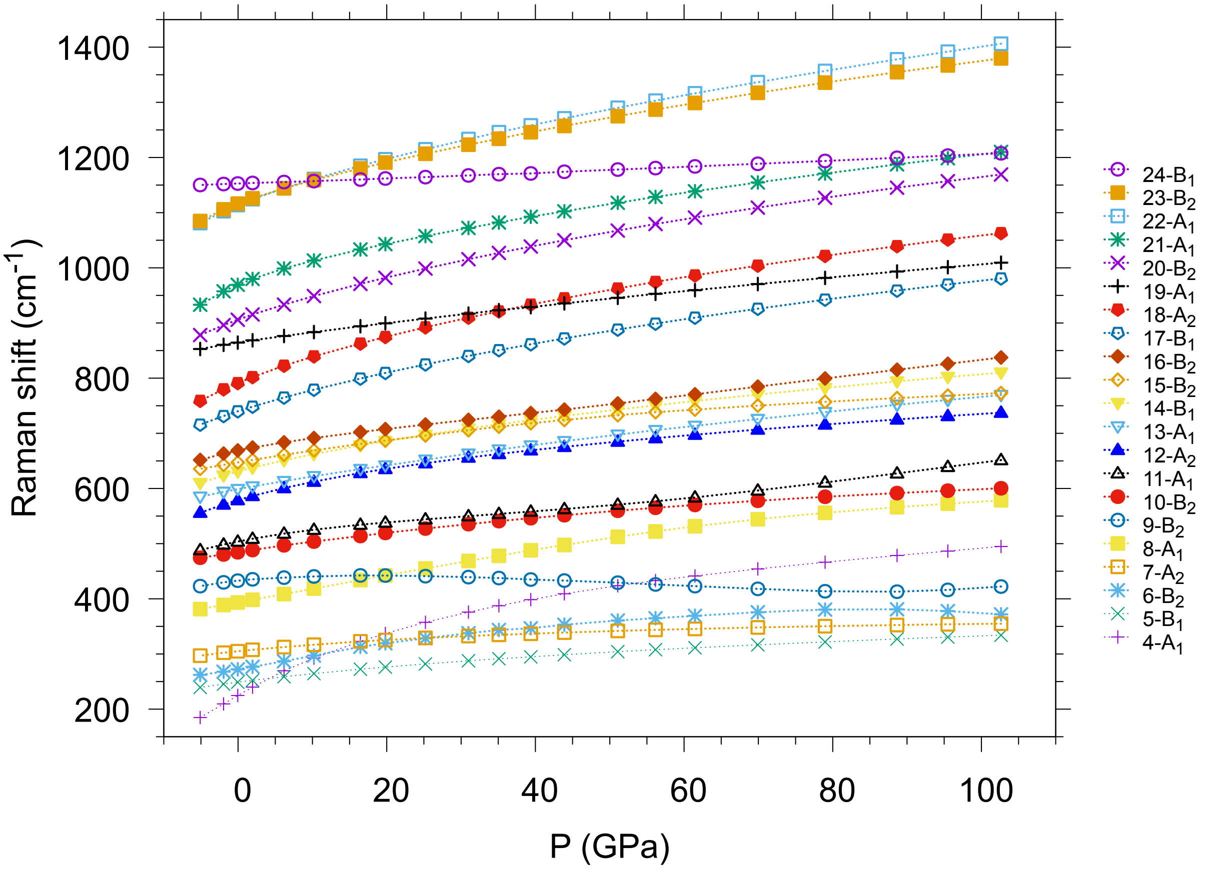

Figure SI-8: Calculated frequency evolution with pressure of the vibrational modes of γ-P_3N_5. The label code indicates number-symmetry of the corresponding vibrational mode. All the vibrational modes of γ-P_3N_5 are Raman active.[S5]

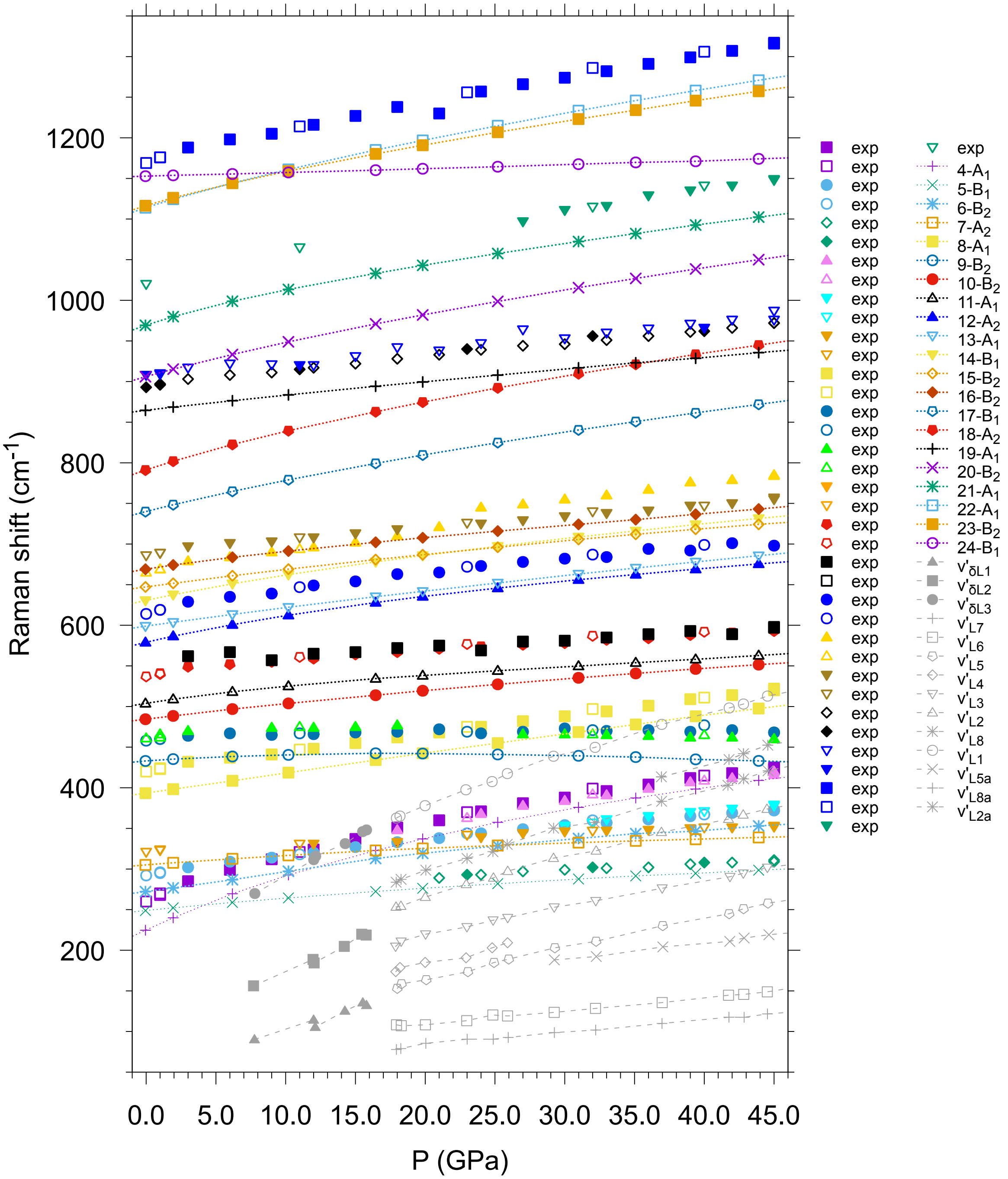

Figure SI-9: Frequency evolution with pressure of the vibrational modes of γ-P_3N_5. The experimental data are plotted as points (exp label in the legend), whereas the calculated frequencies are plotted as dotted lines points (labeled as number of mode-symmetry in the legend). Among the experimental points, the full symbols were acquired on compression and the empty symbols on decompression. The lattice modes of the softer N_2 molecular crystalline structures, taken from and labeled according to ref.,[S7] are also plotted, to evidence their markedly different behavior with pressure comparing to those of stiffer γ-P_3N_5 (grey dashed lines connecting solid squares, circles and up triangles correspond to δ- N_2, whereas dahsed lines connecting empty symbols above 16 GPa correspond to ϵ-N_2).

Acknowledgements

Thanks are expressed to EC through the European Research Council (ERC) for funding the project PHOSFUN "Phosphorene functionalization: a new platform for advanced multifunctional materials" (Grant Agreement No. 670173) through an ERC Advanced Grant. This study was supported by the Deep Carbon Observatory (DCO) initiative under the project *Physics and Chemistry of Carbon at Extreme Conditions*, by the project "GreenPhos - alta pressione", by the Italian Ministero dell'Università e della Ricerca (MUR) under the project PRIN 2017 KFY7XF FERMAT "FastElectRon dynamics in novel hybrid-2D MATerials", and by Fondazione Cassa di Risparmio di Firenze under the project HP-PHOTOCHEM. The authors acknowledge the European Synchrotron Radiation Facility (ESRF) for provision of synchrotron radiation facilities and thank V. Svitlyk, G. Garbarino, M. Mezouar for assistance in using beamlines ID27. JMR acknowledges financial support from the Spanish National Research Agency (AEI) under projects PGC18-094814-B-C22 and RED2018-102612-T, and Principado de Asturias (FICYT) and FEDER under project AYUD/2021/51036. F.I.R. thanks the Government of Principado de Asturias for its FICYT grant number AYUD/2021/58773.

Supporting References

(S1) Petříček, V.; Dušek, M.; Palatinus, L. Crystallographic Computing System JANA2006: General features. *Z. Krist.-Cryst. Mater.* **2014**, *229*, 345–352, DOI: 10.1515/zkri-2014-1737.

(S2) Angel, R. J. Equations of State. *Rev. Mineral. Geochem.* **2000**, *41*, 35, DOI: 10.2138/rmg.2000.41.2.

(S3) Angel, R. J.; Alvaro, M.; Gonzalez-Platas, J. EosFit7c and a Fortran module (library) for equation of state calculations. *Z. Krist.-Cryst. Mater.* **2014**, *229*, 405–419, DOI: doi:10.1515/zkri-2013-1711.

(S4) Angel, R.; Mazzucchelli, M.; Gonzalez-Platas, J.; Alvaro, M. A self-consistent approach to describe unit-cell-parameter and volume variations with pressure and temperature. *J. Appl. Cryst.* **2021**, *54*, 1621–1630, DOI: 10.1107/S1600576721009092.

(S5) Kroll, P.; Schnick, W. A Density Functional Study of Phosphorus Nitride P_3N_5: Refined Geometries, Properties, and Relative Stability of α-P_3N_5 and γ-P_3N_5 and a Further Possible High-Pressure Phase δ-P_3N_5 with Kyanite-Type Structure. *Chem. Eur. J.* **2002**, *8*, 3530–3537, DOI: 10.1002/1521-3765(20020802)8:15<3530::AID-CHEM3530>3.0.CO;2-6.

(S6) Horstmann, S.; Irran, E.; Schnick, W. Synthesis and Crystal Structure of Phosphorus(V) Nitride α-P_3N_5. *Angew. Chem. Int. Ed.* **1997**, *36*, 1873–1875, DOI: 10.1002/anie.199718731.

(S7) Schneider, H.; Häfner, W.; Wokaun, A.; Olijnyk, H. Room temperature Raman scattering studies of external and internal modes of solid nitrogen at pressures $8 \leq P \leq 54$ GPa. *J. Chem. Phys.* **1992**, *96*, 8046–8053, DOI: 10.1063/1.462356.
