# Supplementary figures and images for: High-Pressure and High-Temperature Chemistry of Phosphorus and Nitrogen: Synthesis and Characterization of α- and γ-P3N5
Source: Inorg Chem. 2022 Jul 26;61(31):12165–80. doi: 10.1021/acs.inorgchem.2c01190 (PMC9374155; doi:10.1021/acs.inorgchem.2c01190)

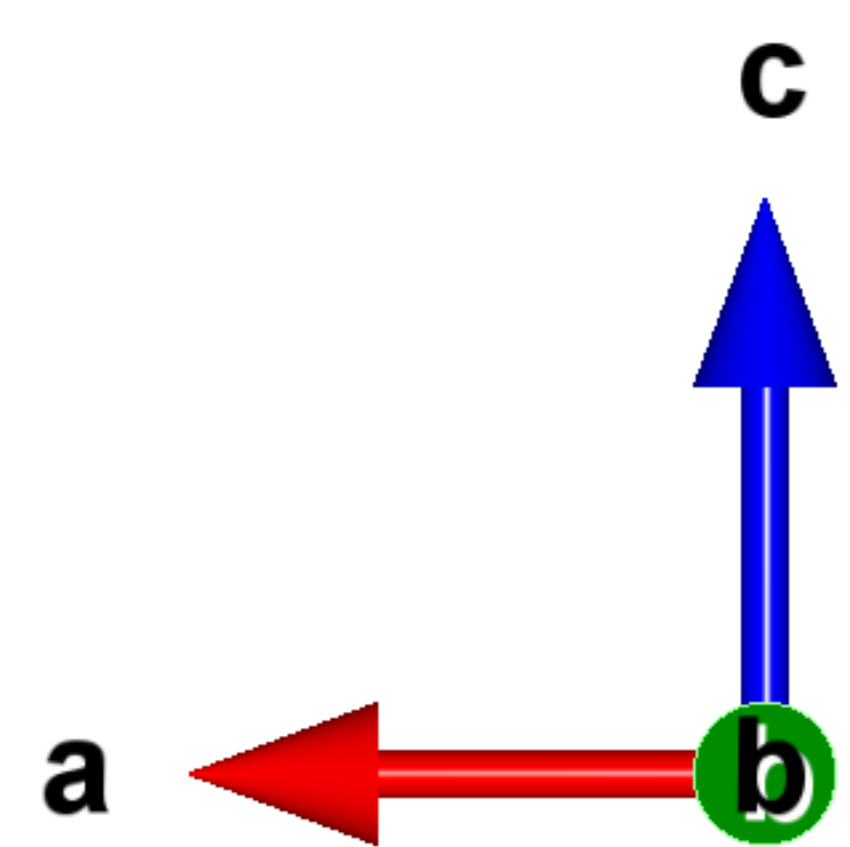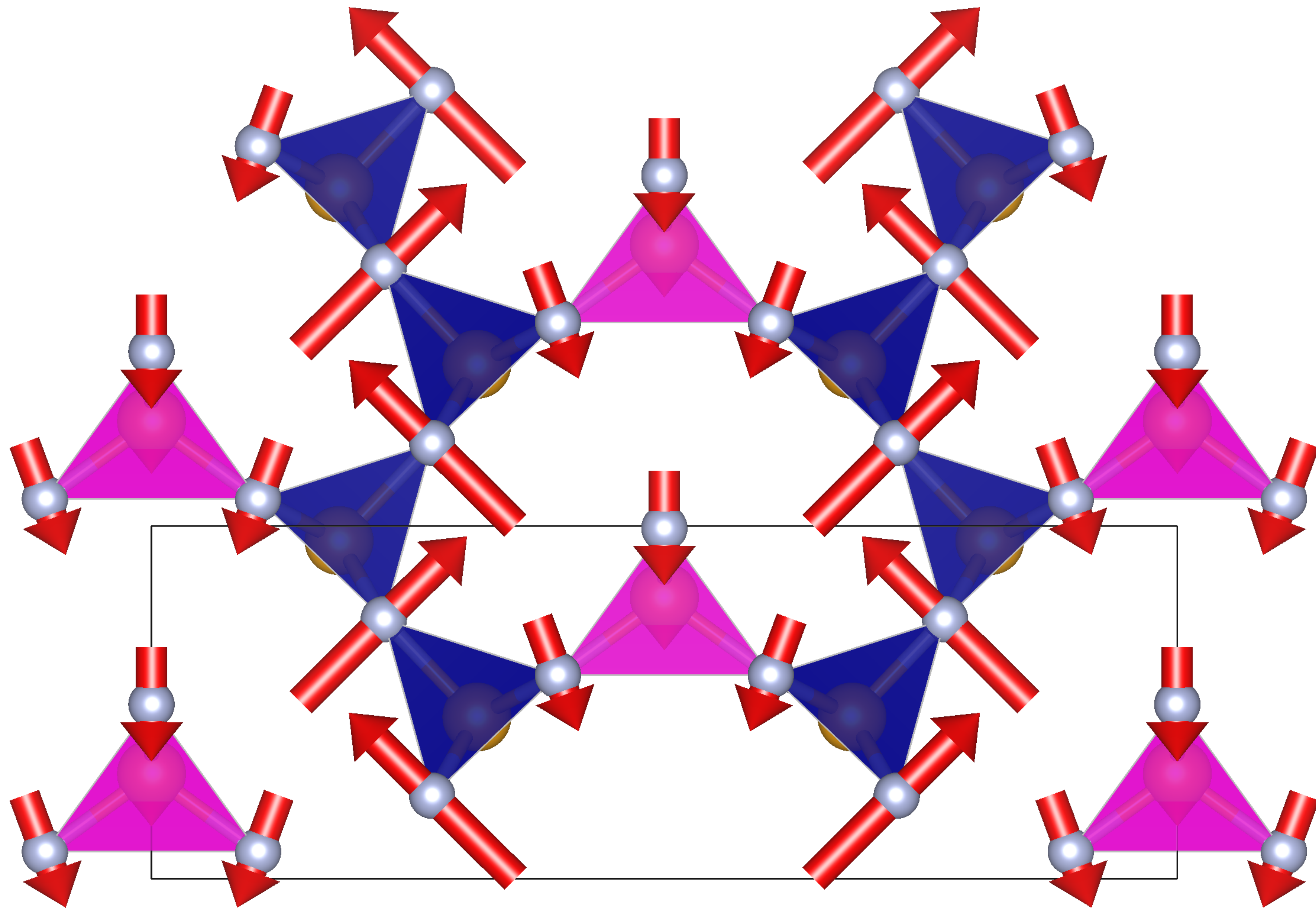

Supplement: Supplementary file 2 — ic2c01190_si_002.zip [file ic2c01190_si_002.zip › 4-A1.pdf]

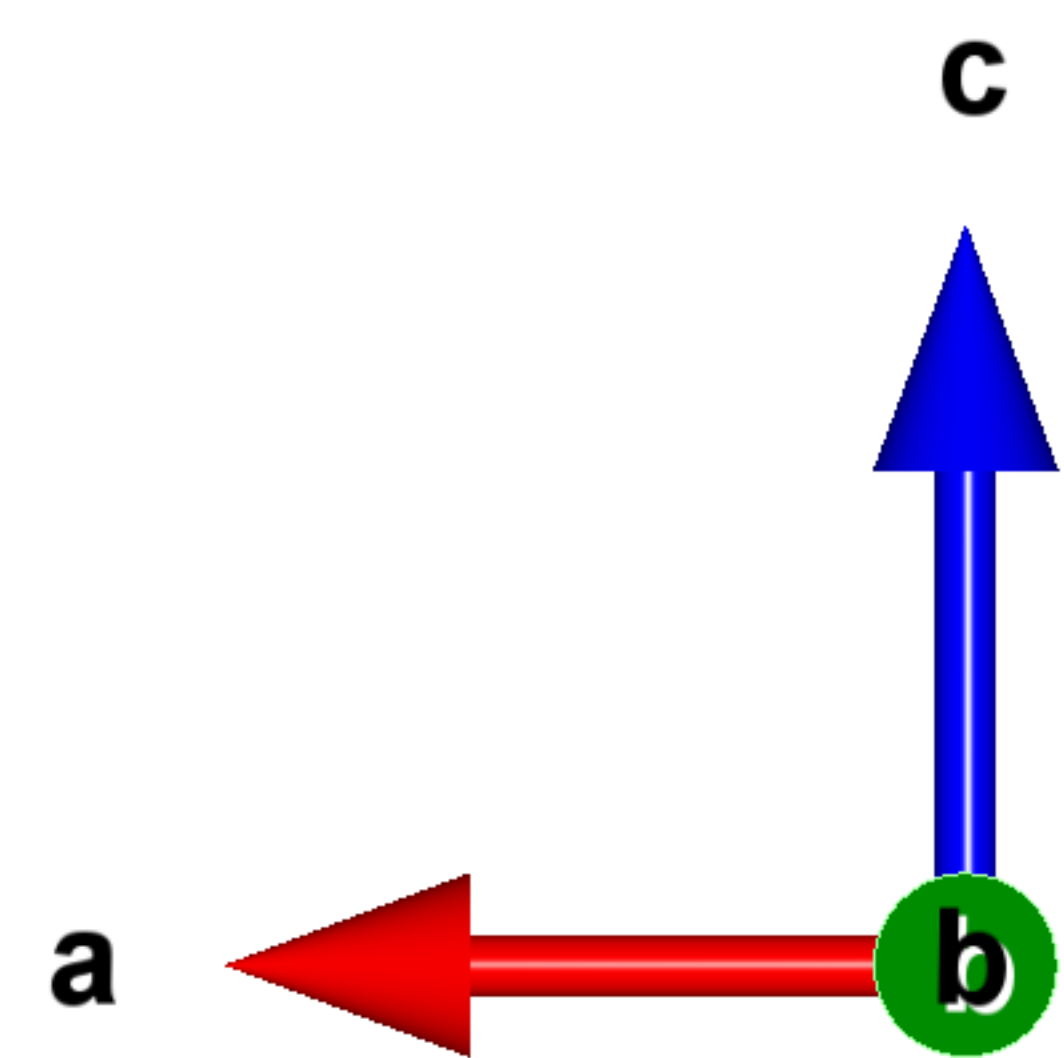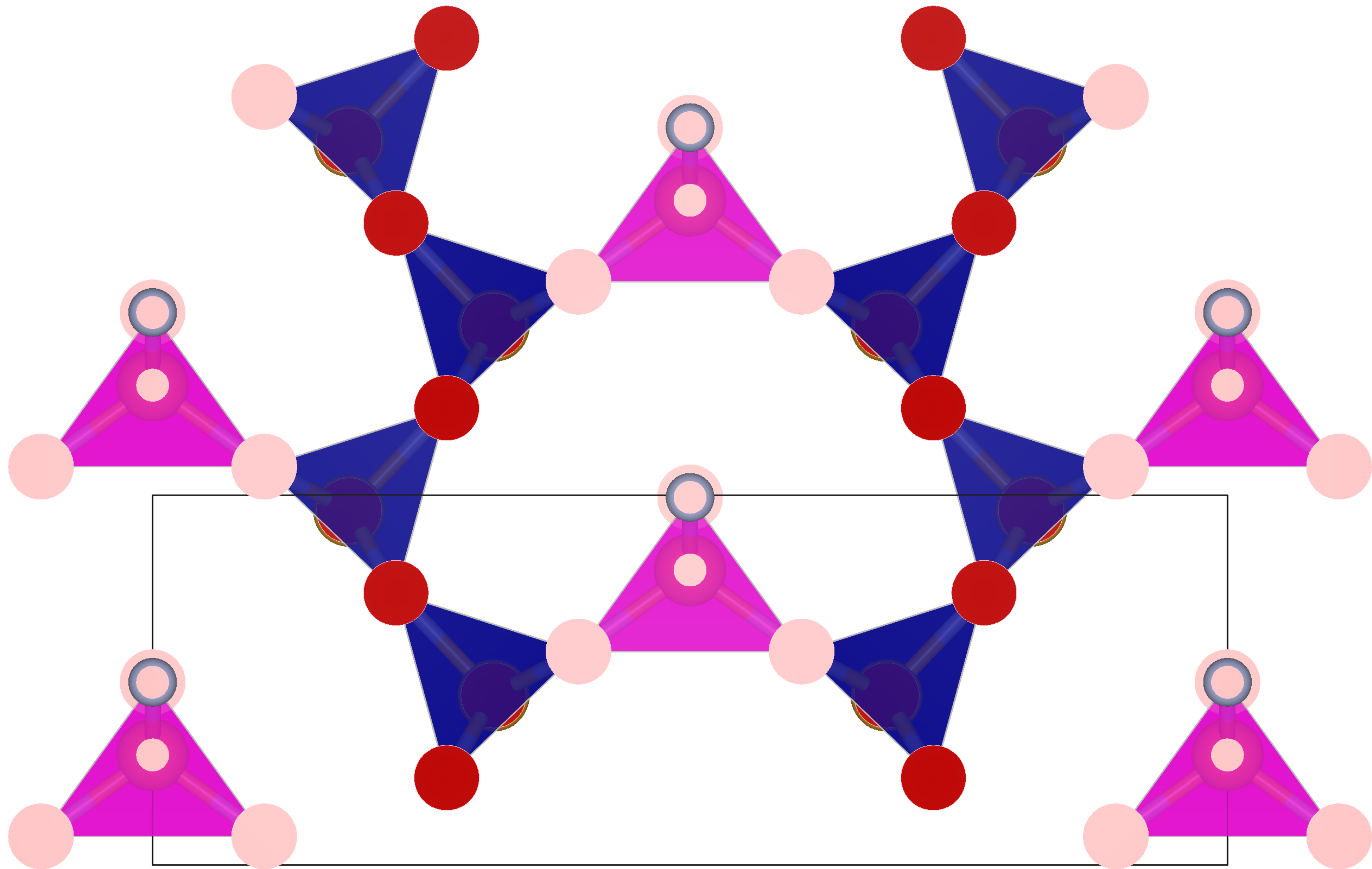

Supplement: Supplementary file 2 — ic2c01190_si_002.zip [file ic2c01190_si_002.zip › 5-B1.pdf]

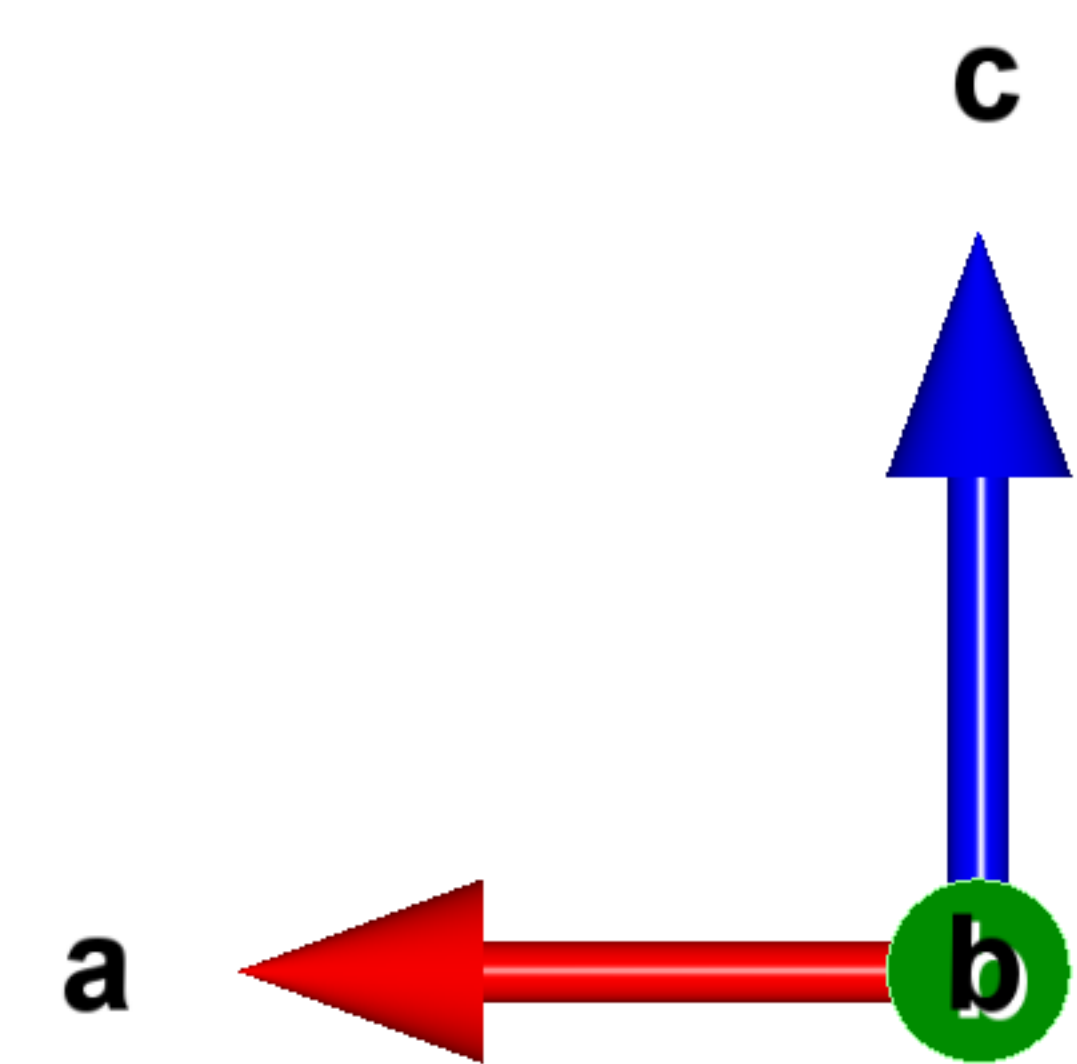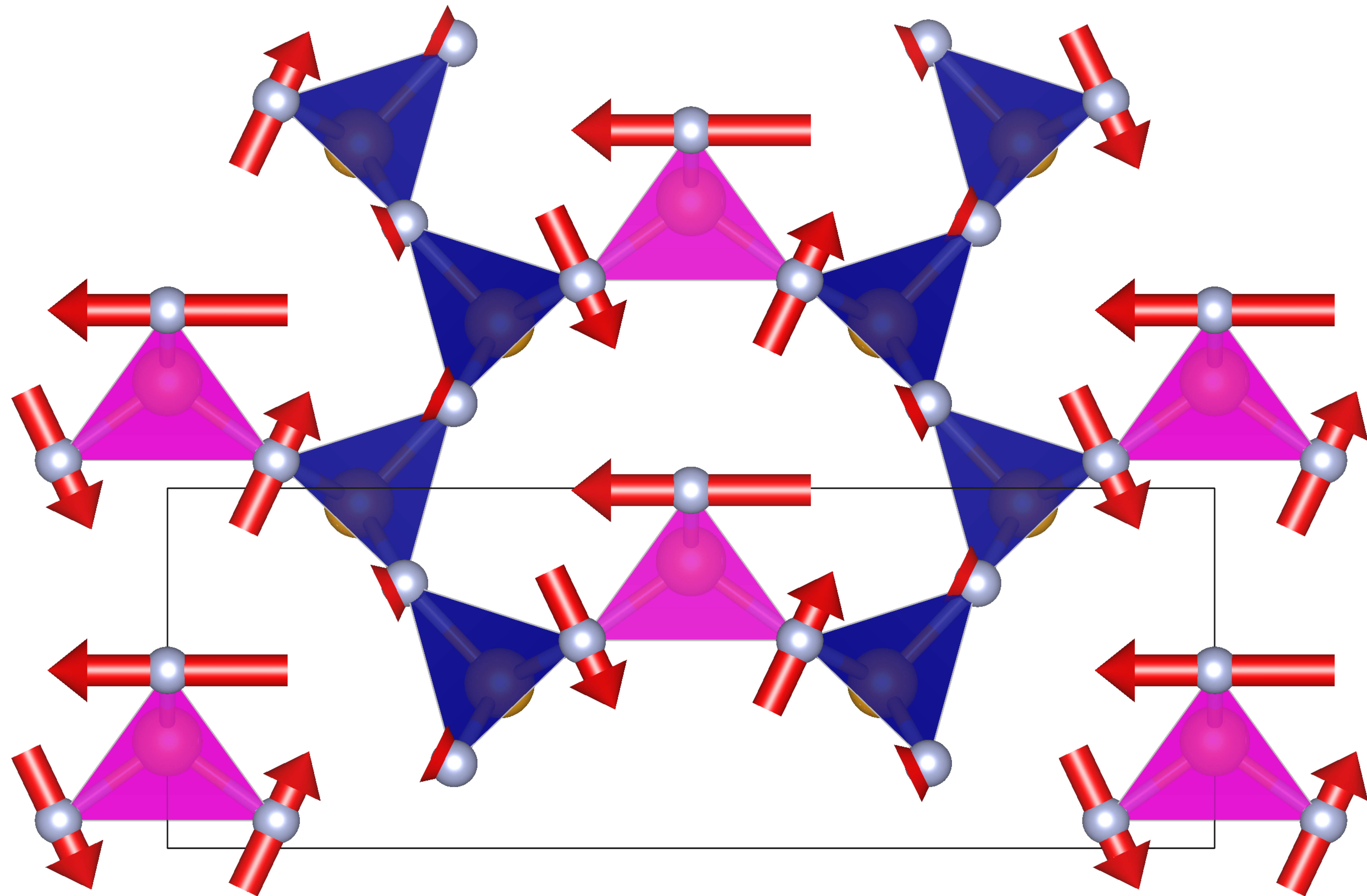

Supplement: Supplementary file 2 — ic2c01190_si_002.zip [file ic2c01190_si_002.zip › 6-B2.pdf]

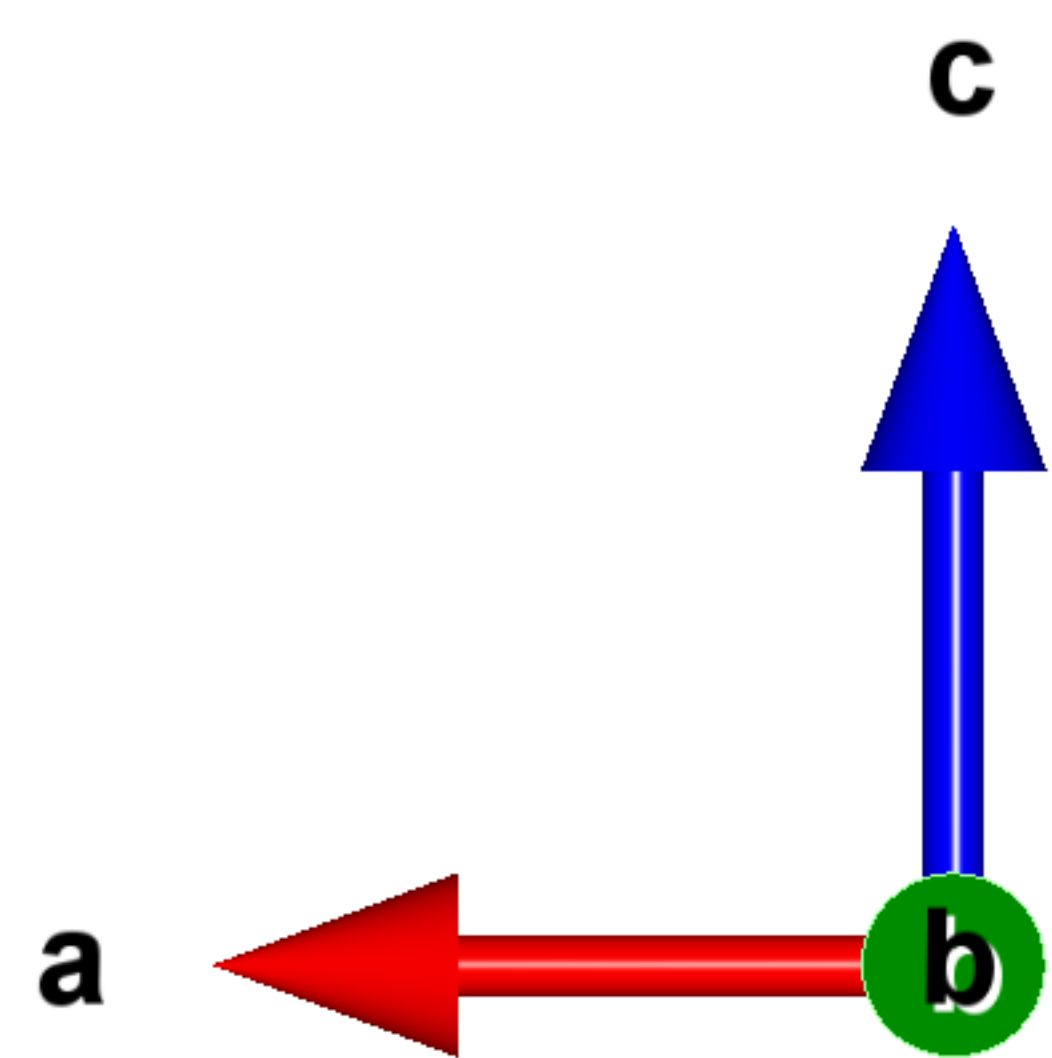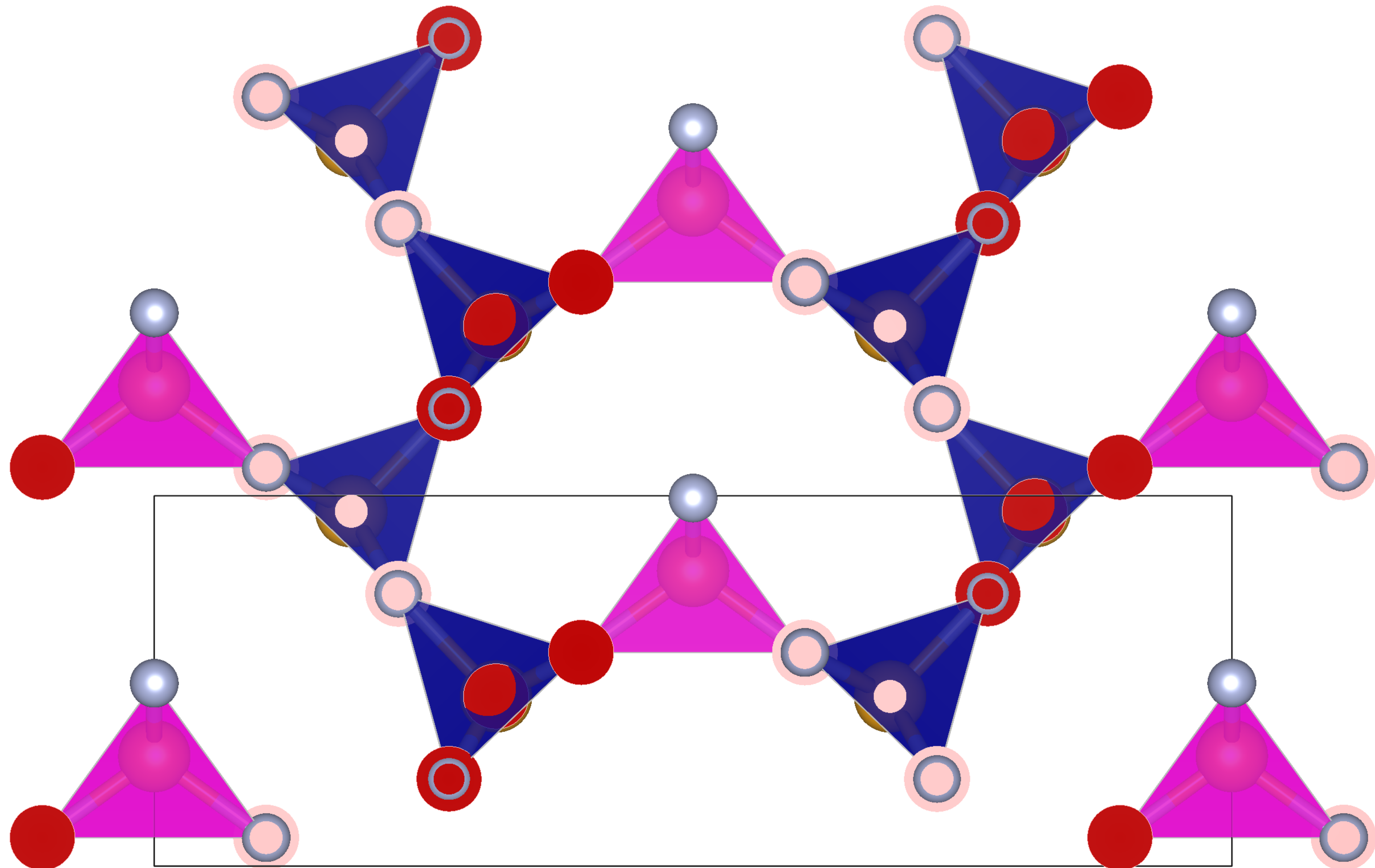

Supplement: Supplementary file 2 — ic2c01190_si_002.zip [file ic2c01190_si_002.zip › 7-A2.pdf]

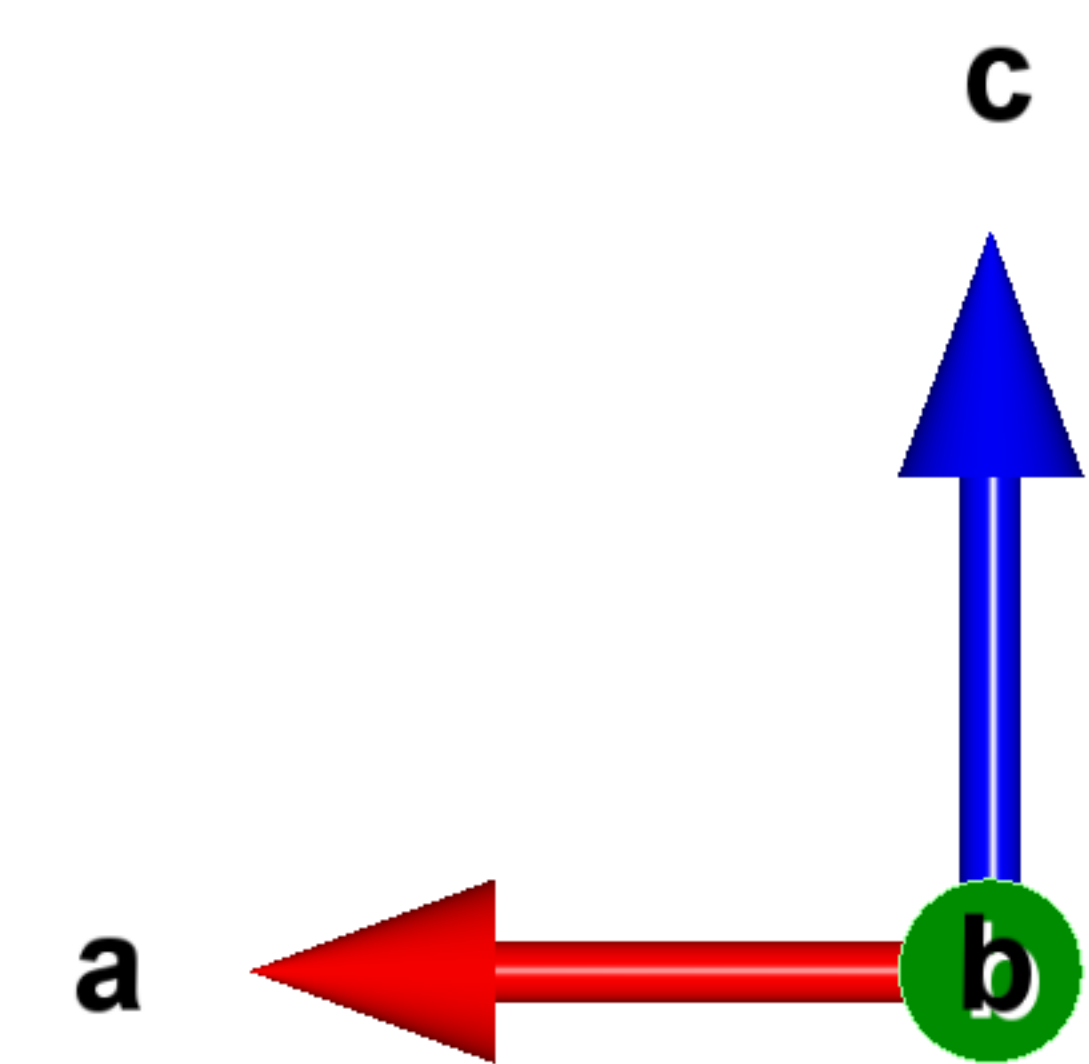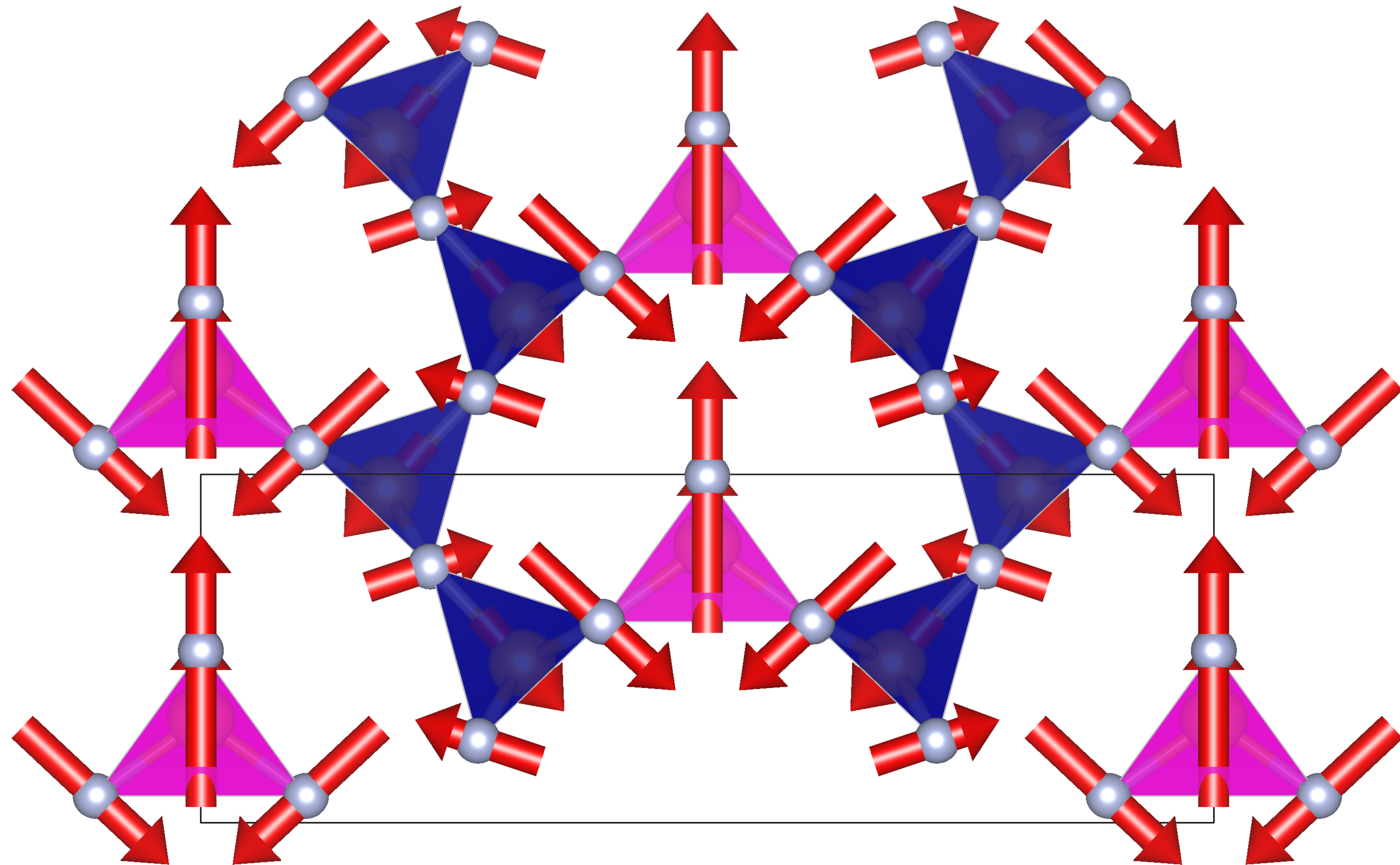

Supplement: Supplementary file 2 — ic2c01190_si_002.zip [file ic2c01190_si_002.zip › 8-A1.pdf]

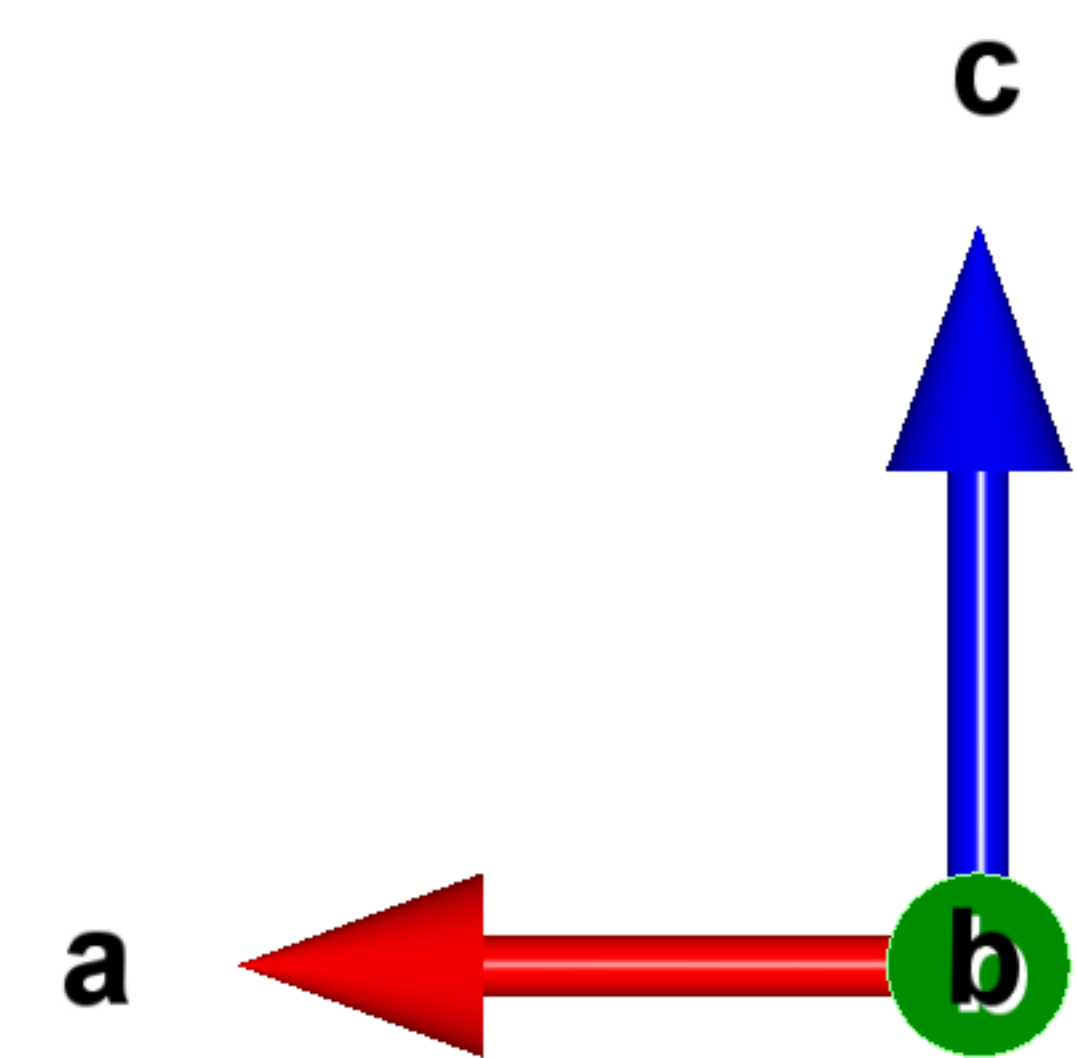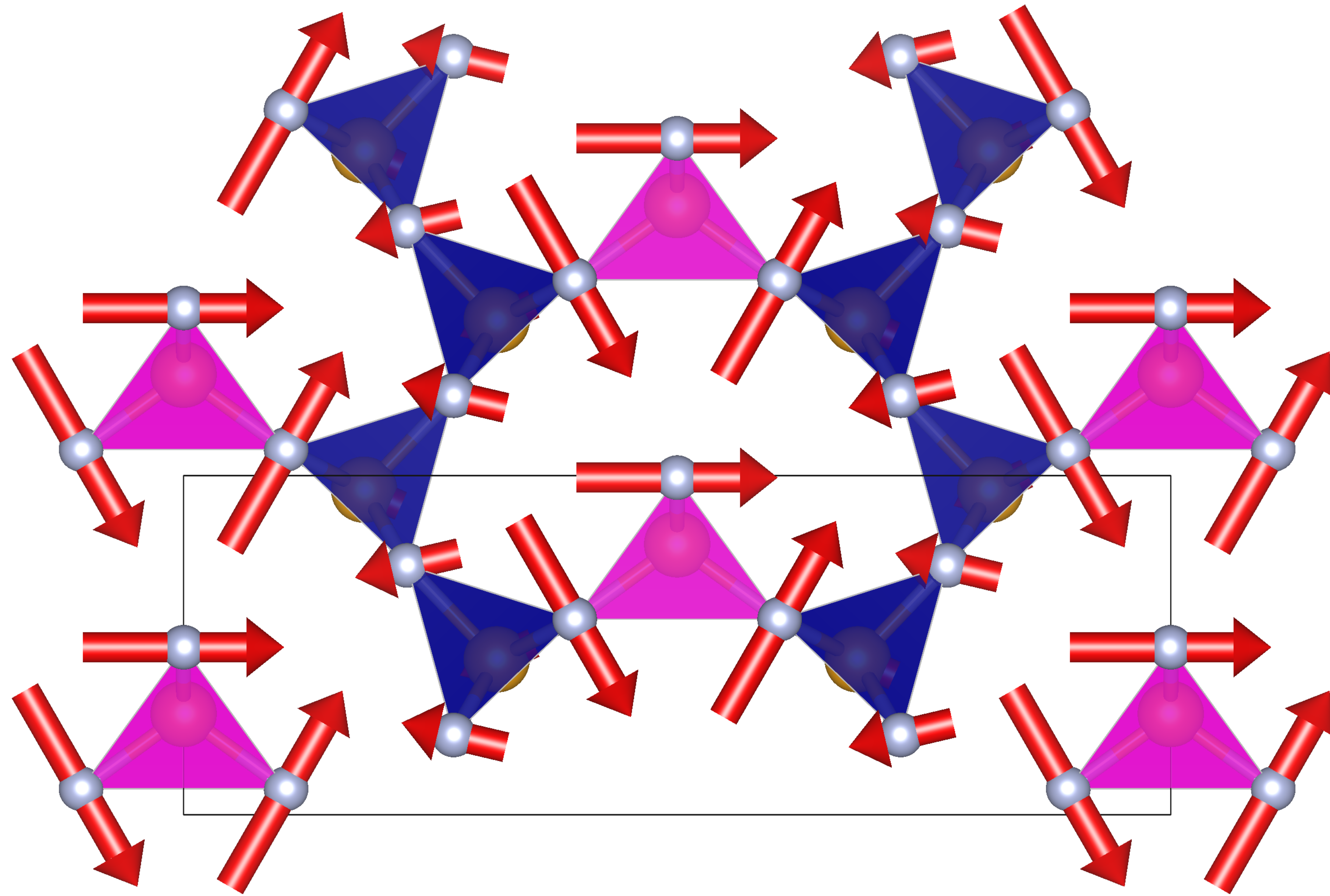

Supplement: Supplementary file 2 — ic2c01190_si_002.zip [file ic2c01190_si_002.zip › 9-B2.pdf]

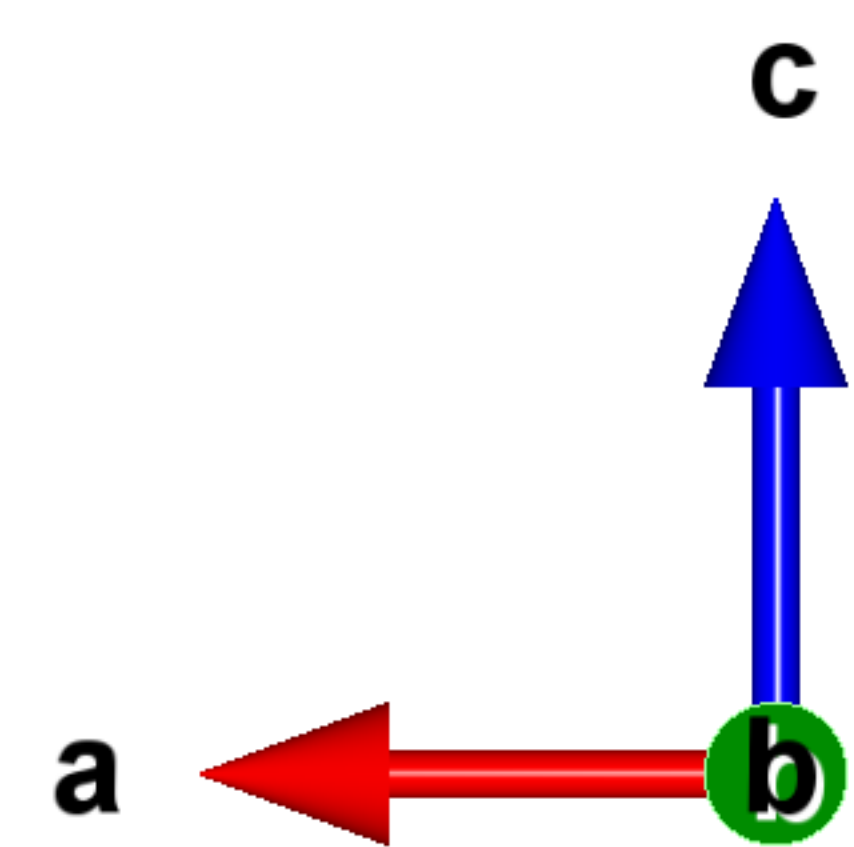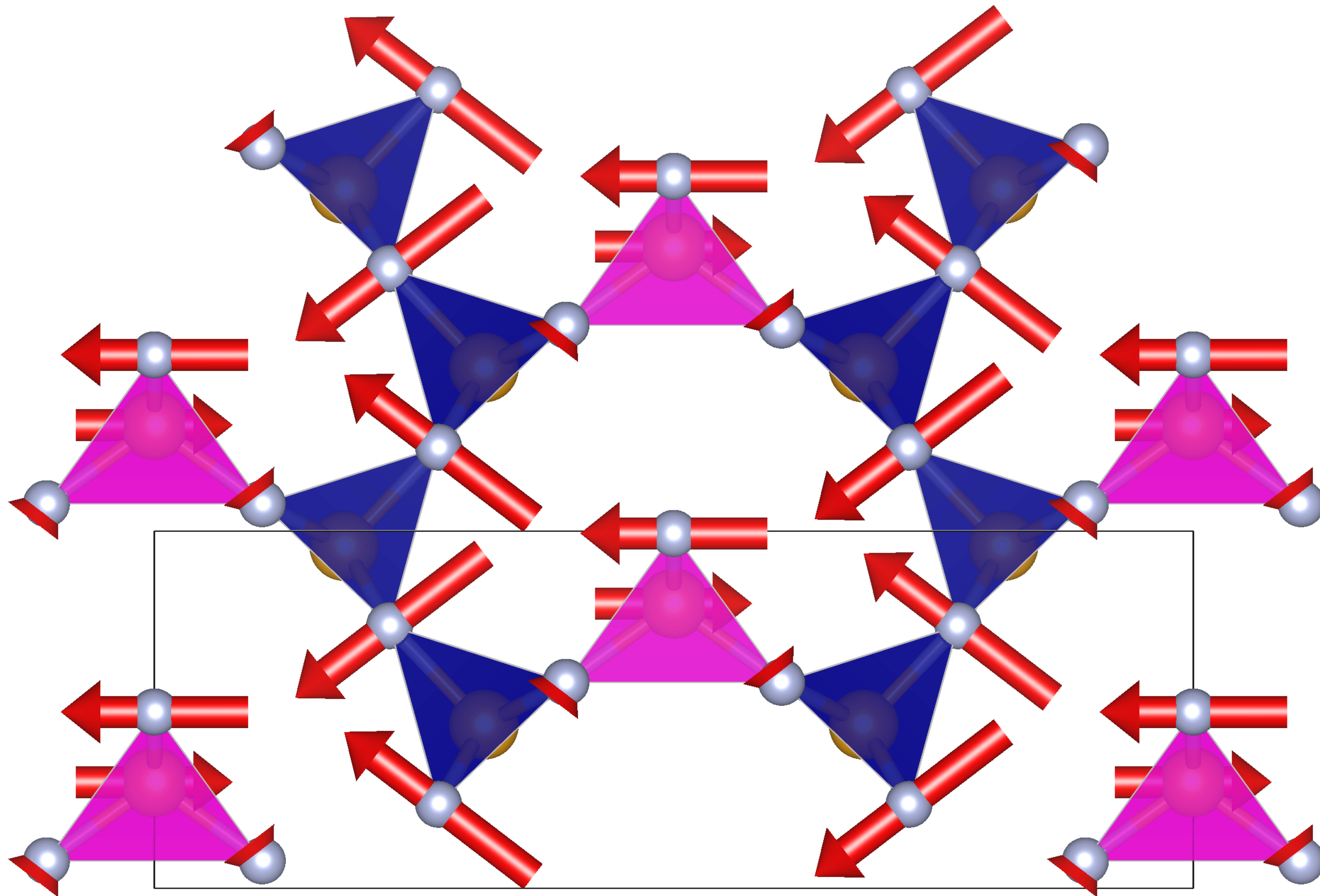

Supplement: Supplementary file 2 — ic2c01190_si_002.zip [file ic2c01190_si_002.zip › 10-B2.pdf]

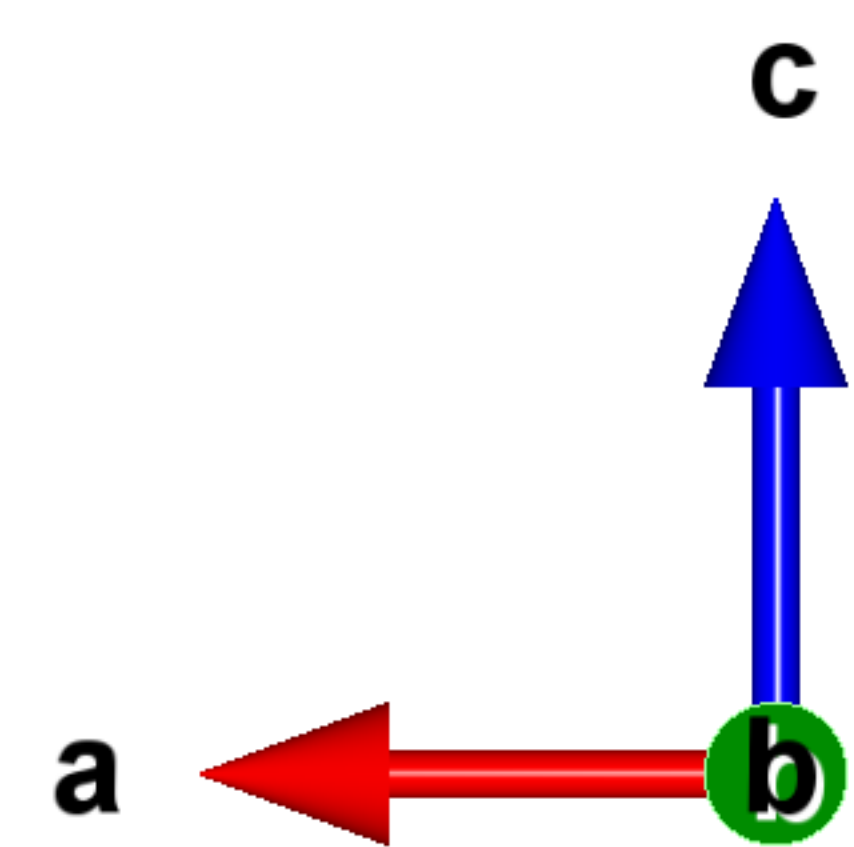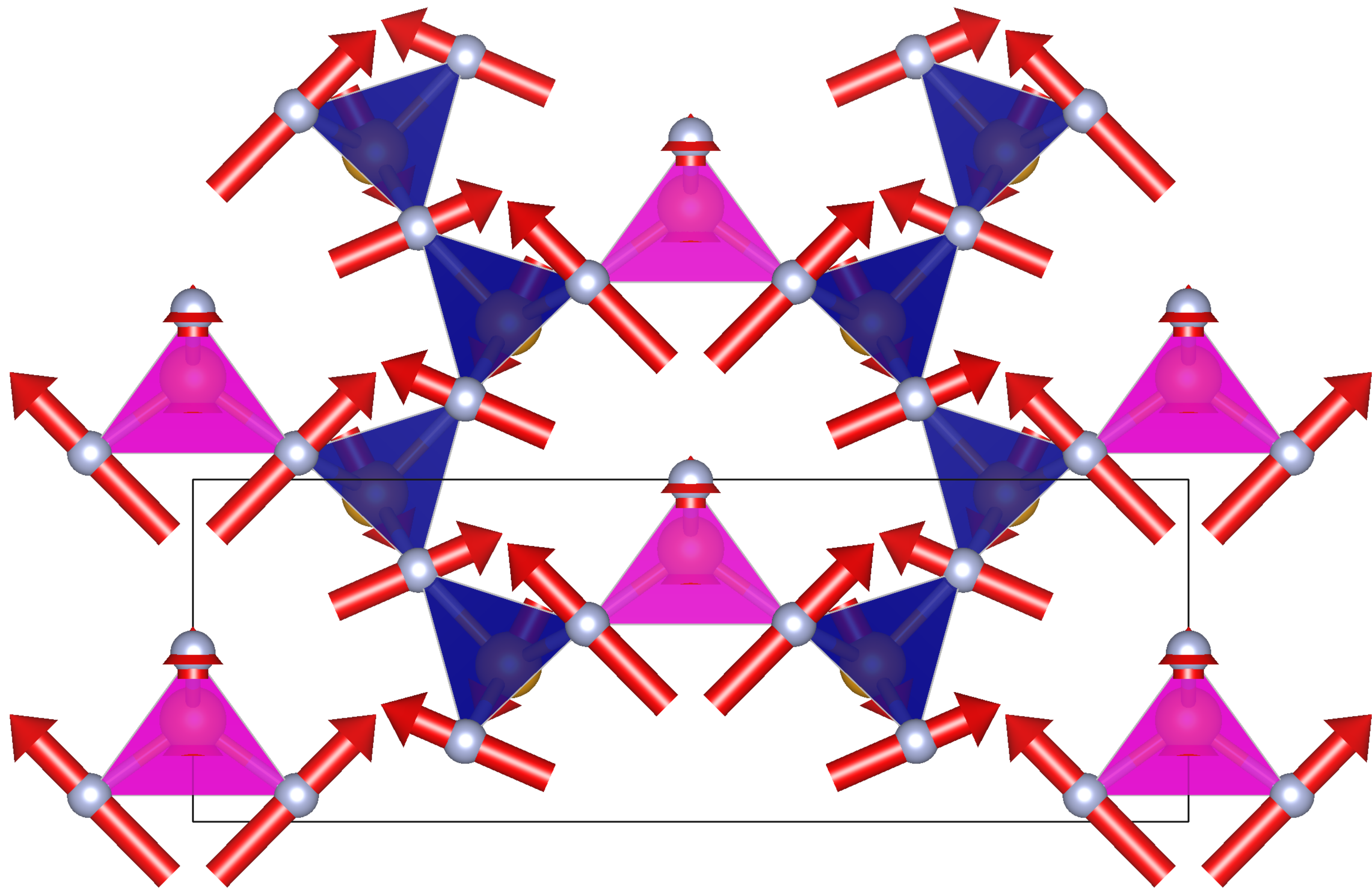

Supplement: Supplementary file 2 — ic2c01190_si_002.zip [file ic2c01190_si_002.zip › 11-A1.pdf]

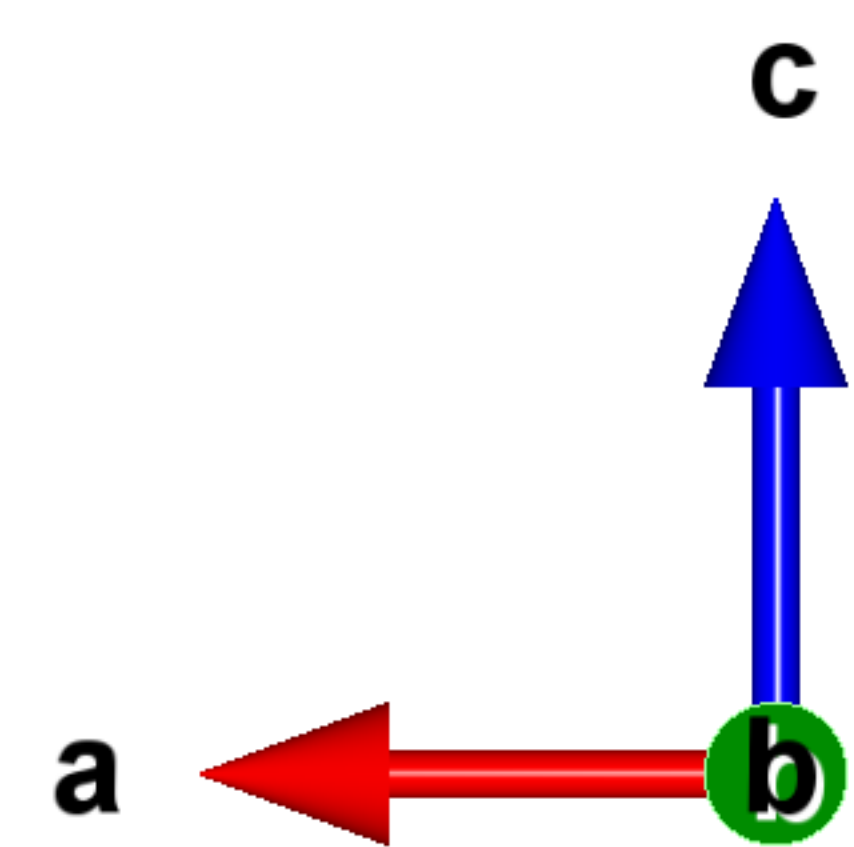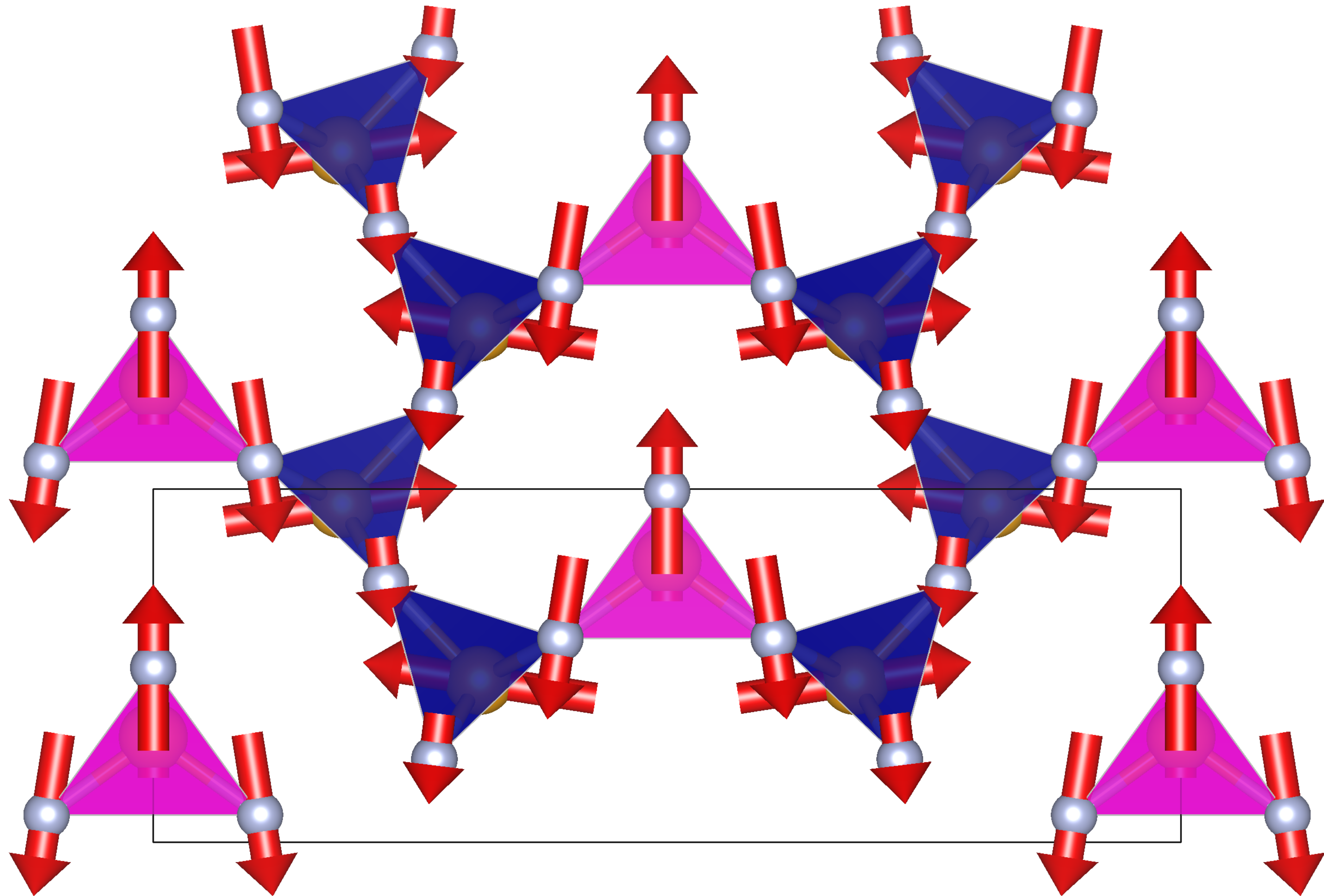

Supplement: Supplementary file 2 — ic2c01190_si_002.zip [file ic2c01190_si_002.zip › 12-A2.pdf]

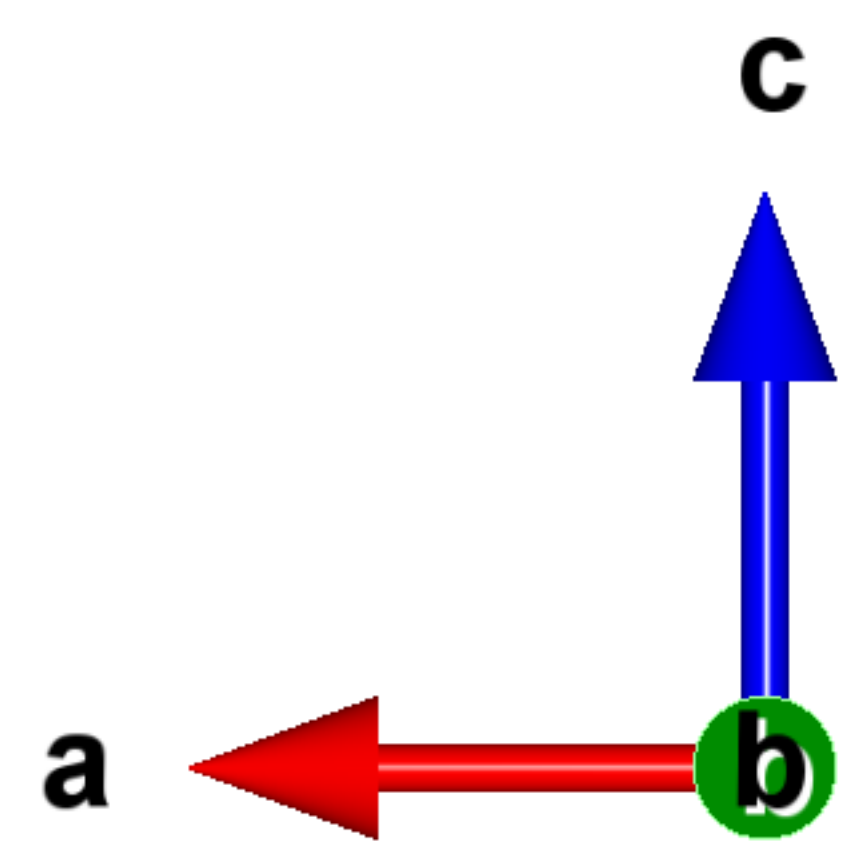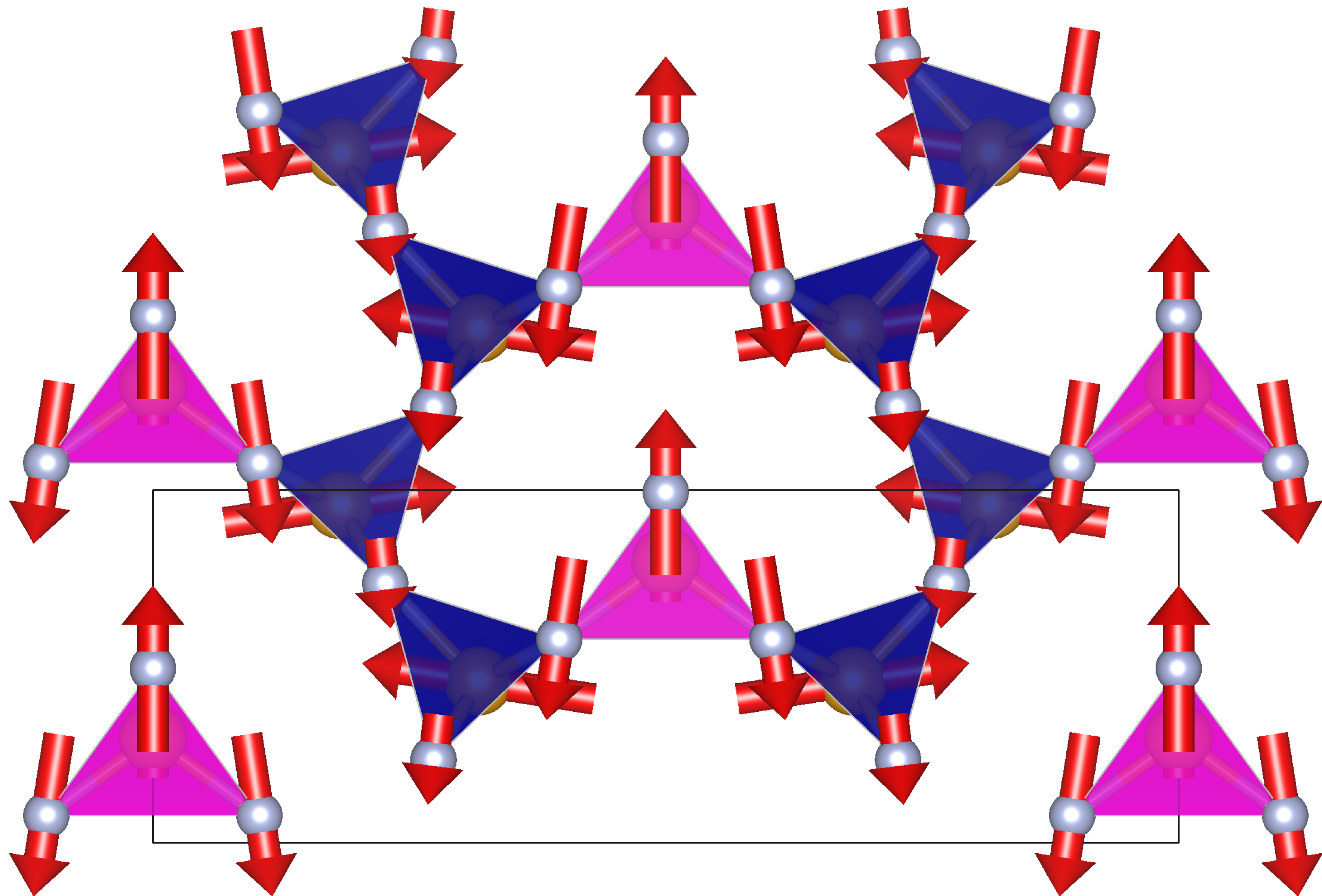

Supplement: Supplementary file 2 — ic2c01190_si_002.zip [file ic2c01190_si_002.zip › 13-A1.pdf]

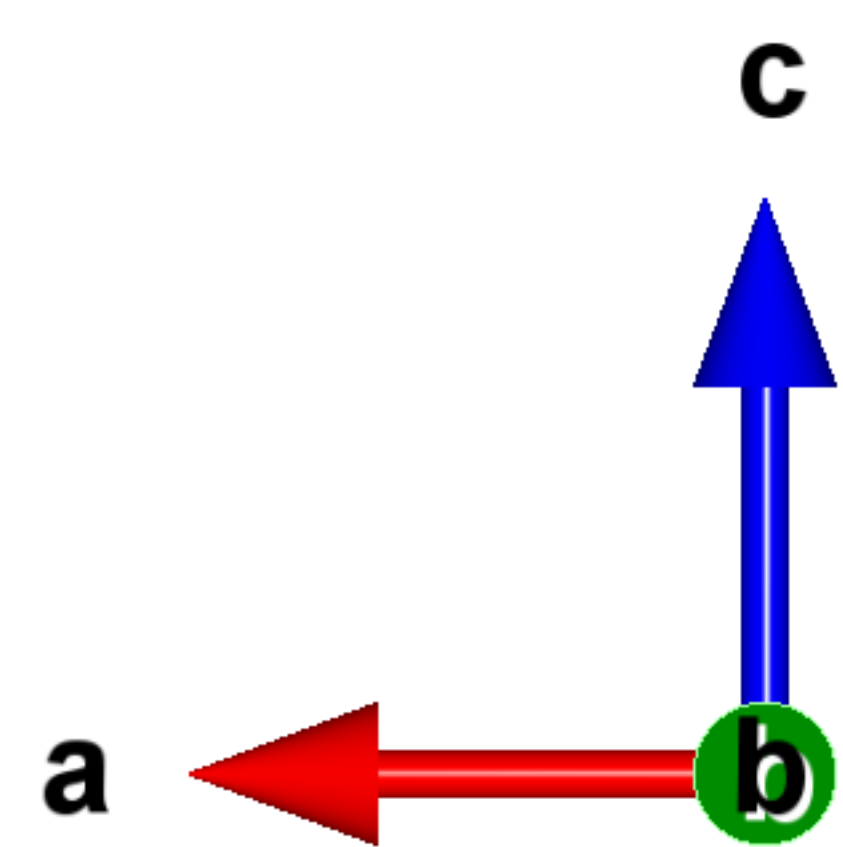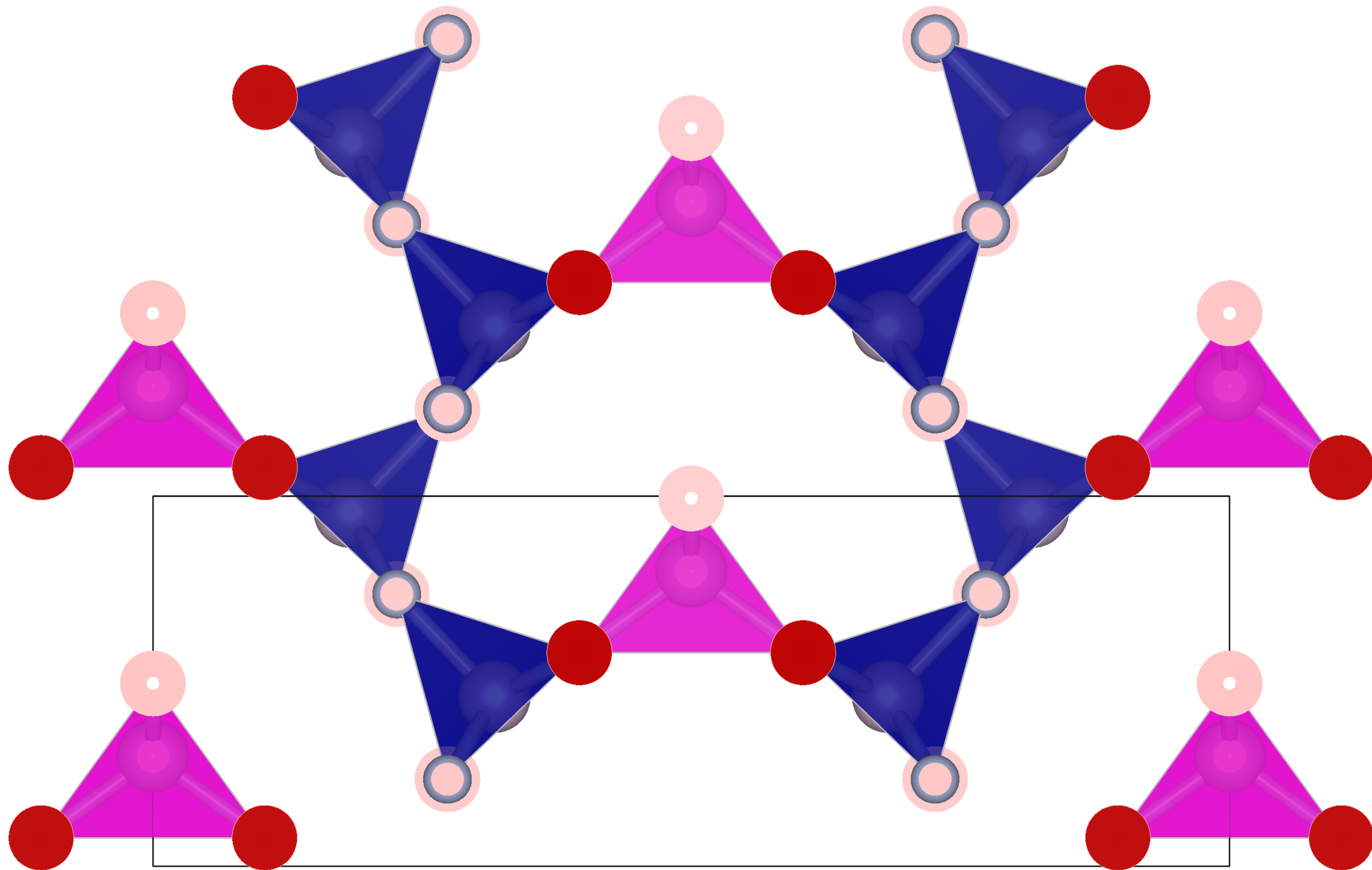

Supplement: Supplementary file 2 — ic2c01190_si_002.zip [file ic2c01190_si_002.zip › 14-B1.pdf]

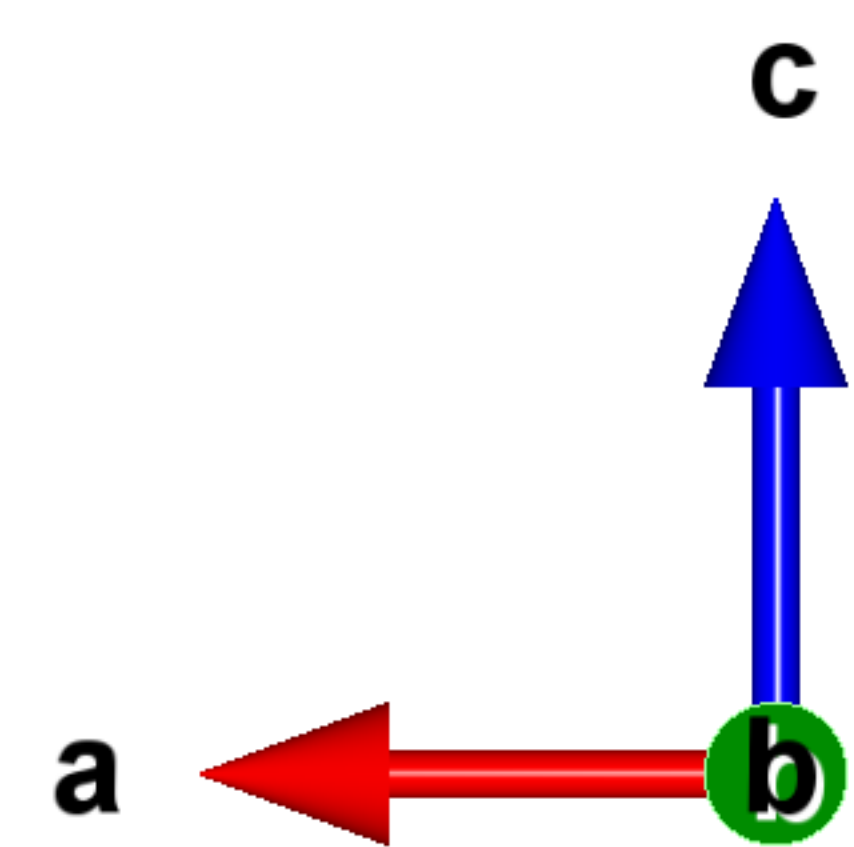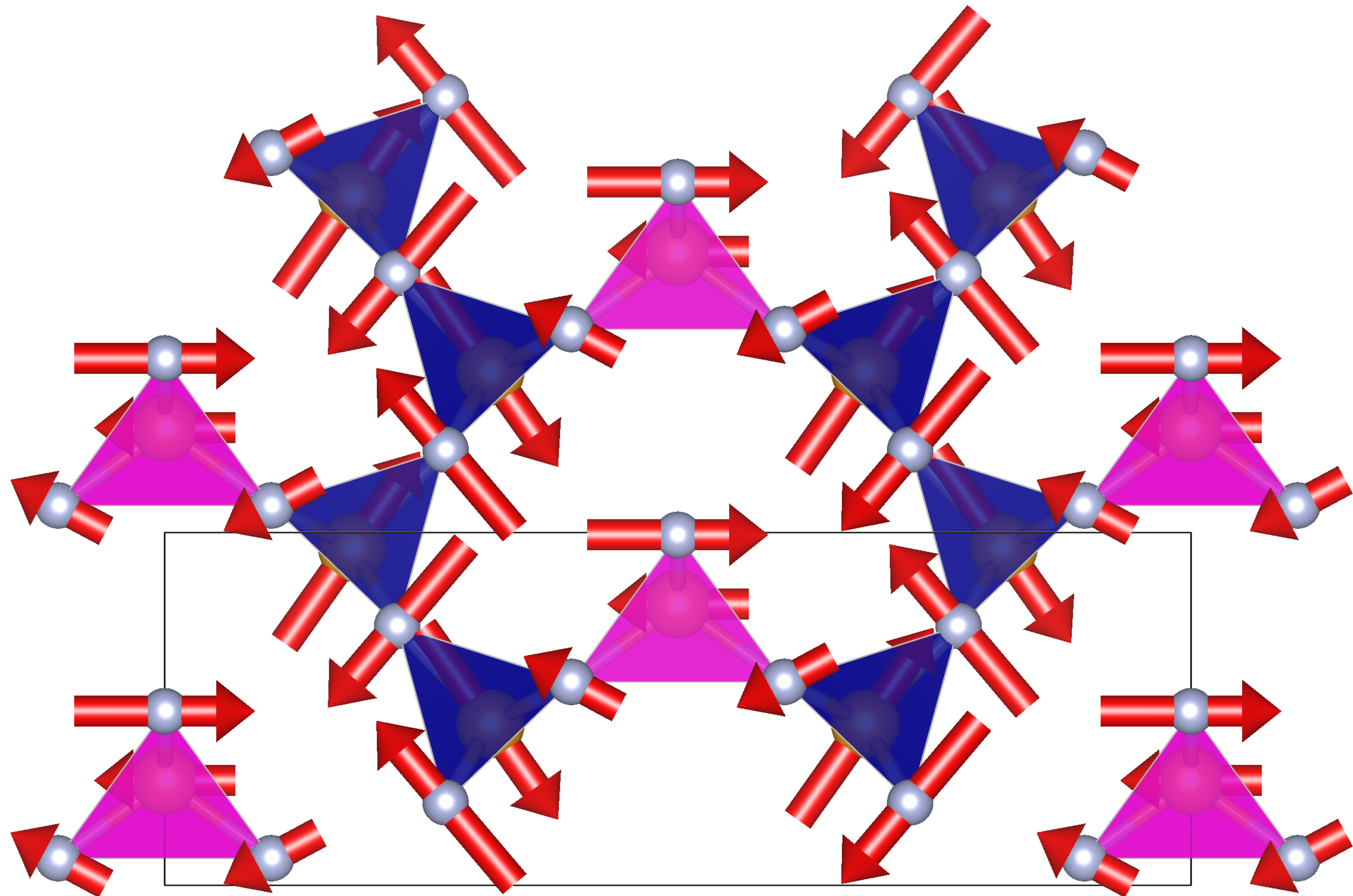

Supplement: Supplementary file 2 — ic2c01190_si_002.zip [file ic2c01190_si_002.zip › 15-B2.pdf]

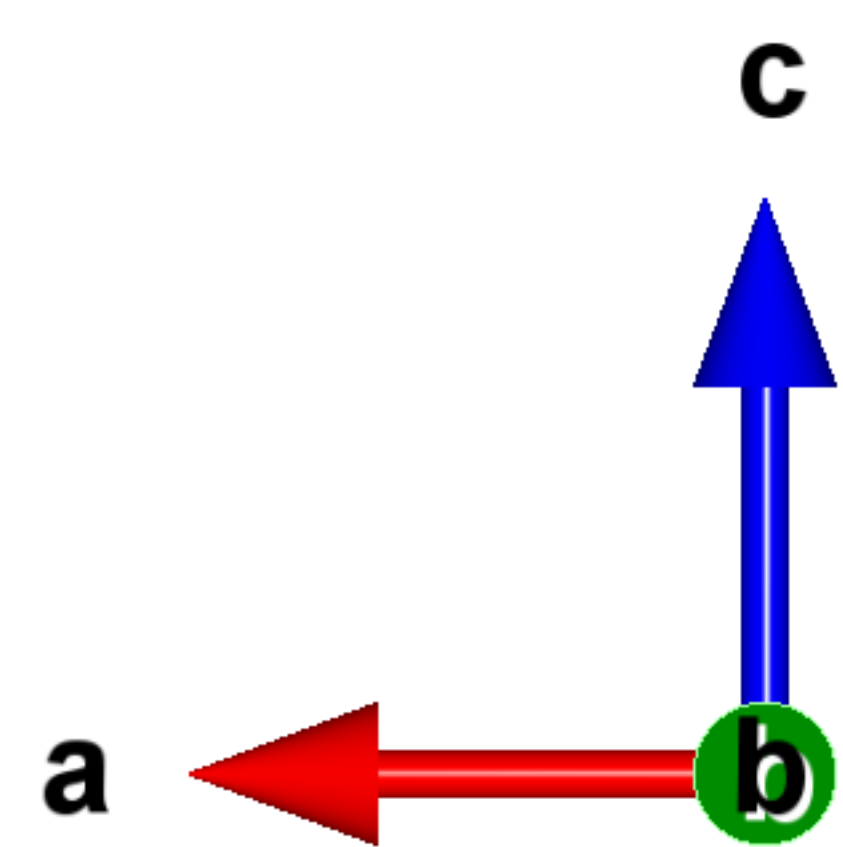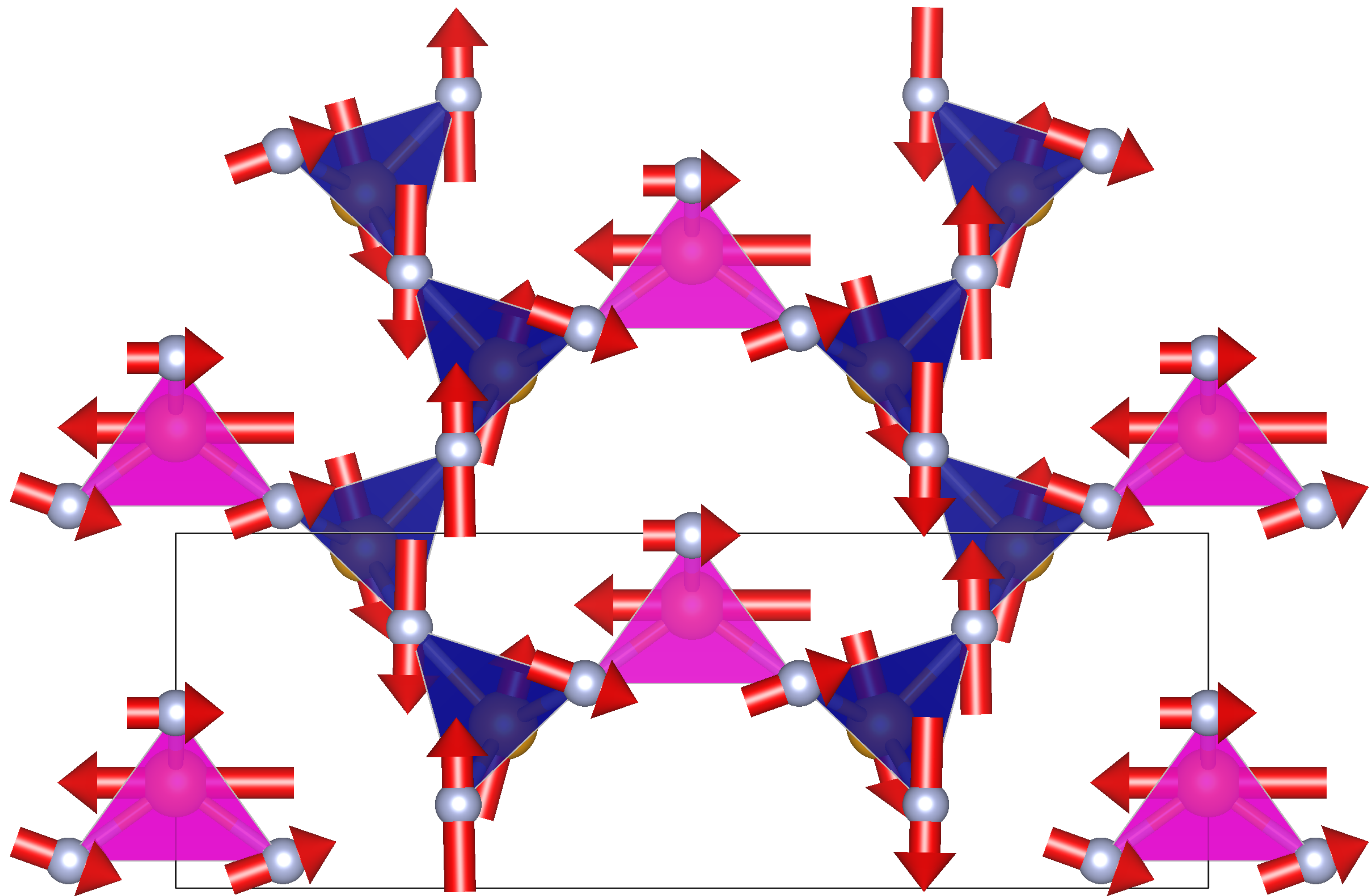

Supplement: Supplementary file 2 — ic2c01190_si_002.zip [file ic2c01190_si_002.zip › 16-B2.pdf]

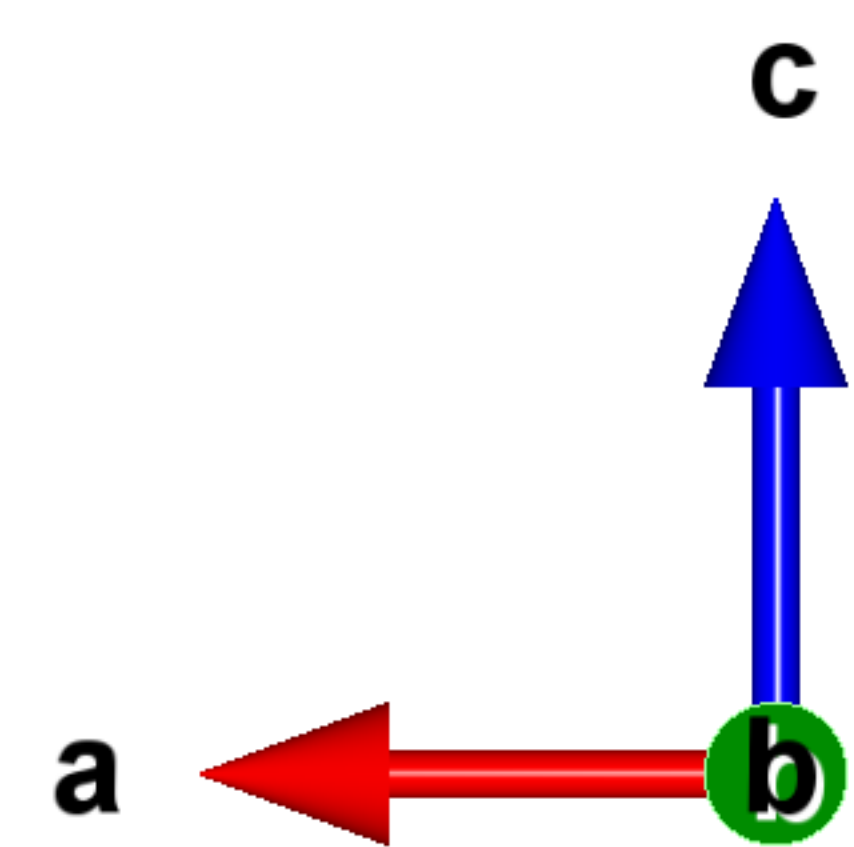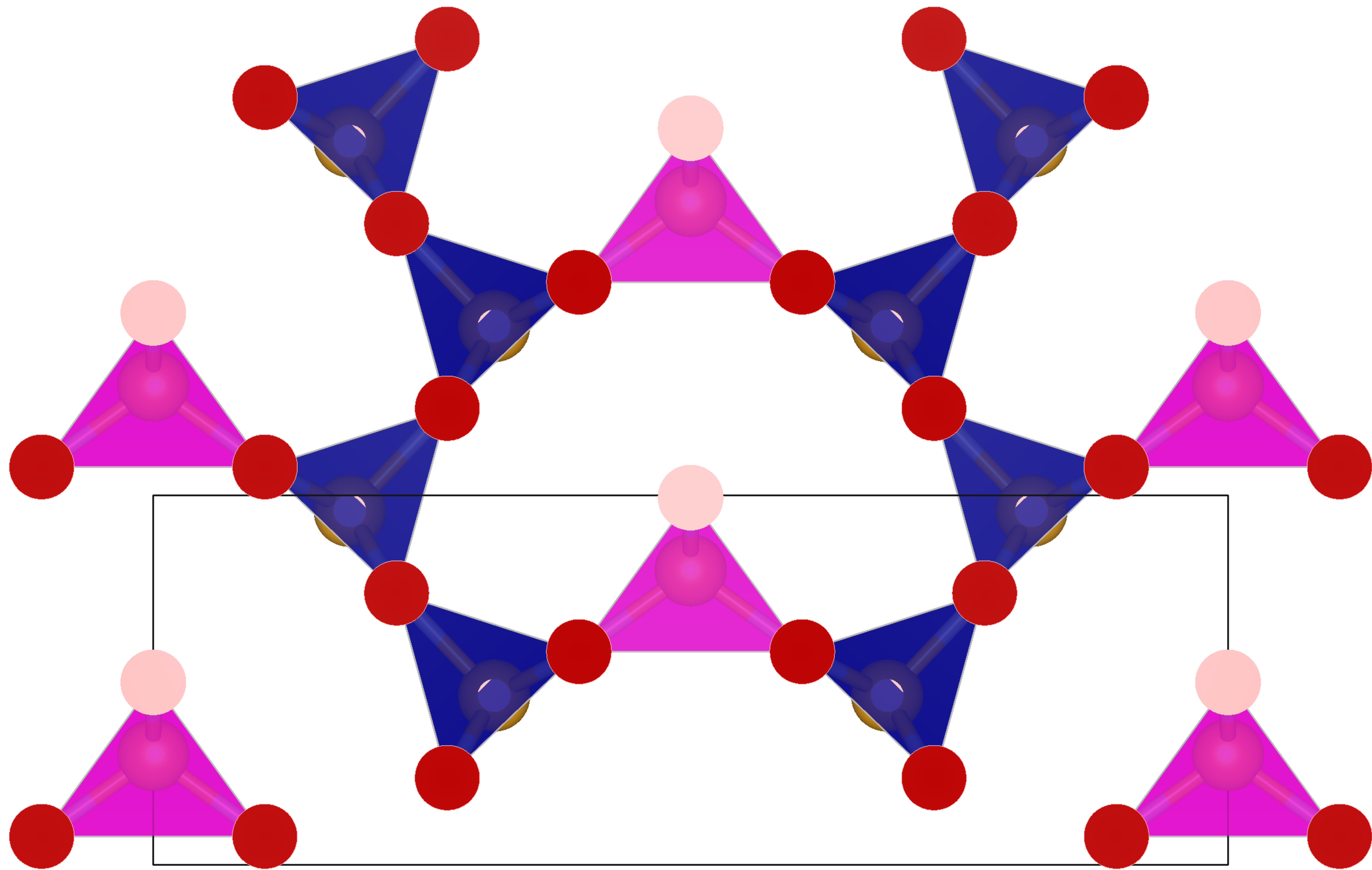

Supplement: Supplementary file 2 — ic2c01190_si_002.zip [file ic2c01190_si_002.zip › 17-B1.pdf]

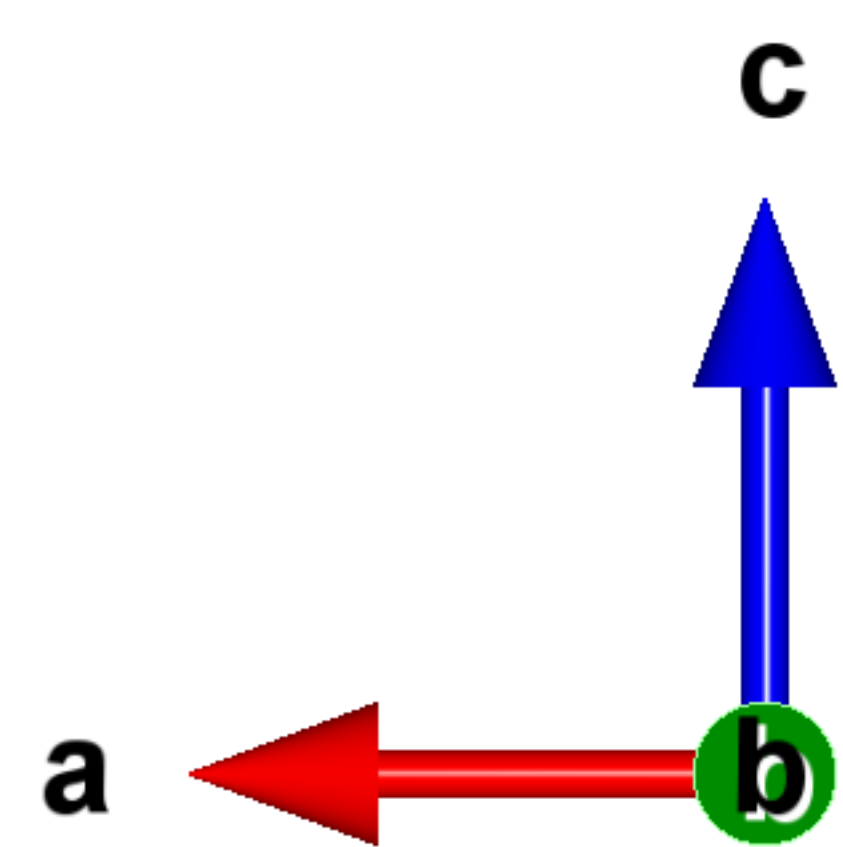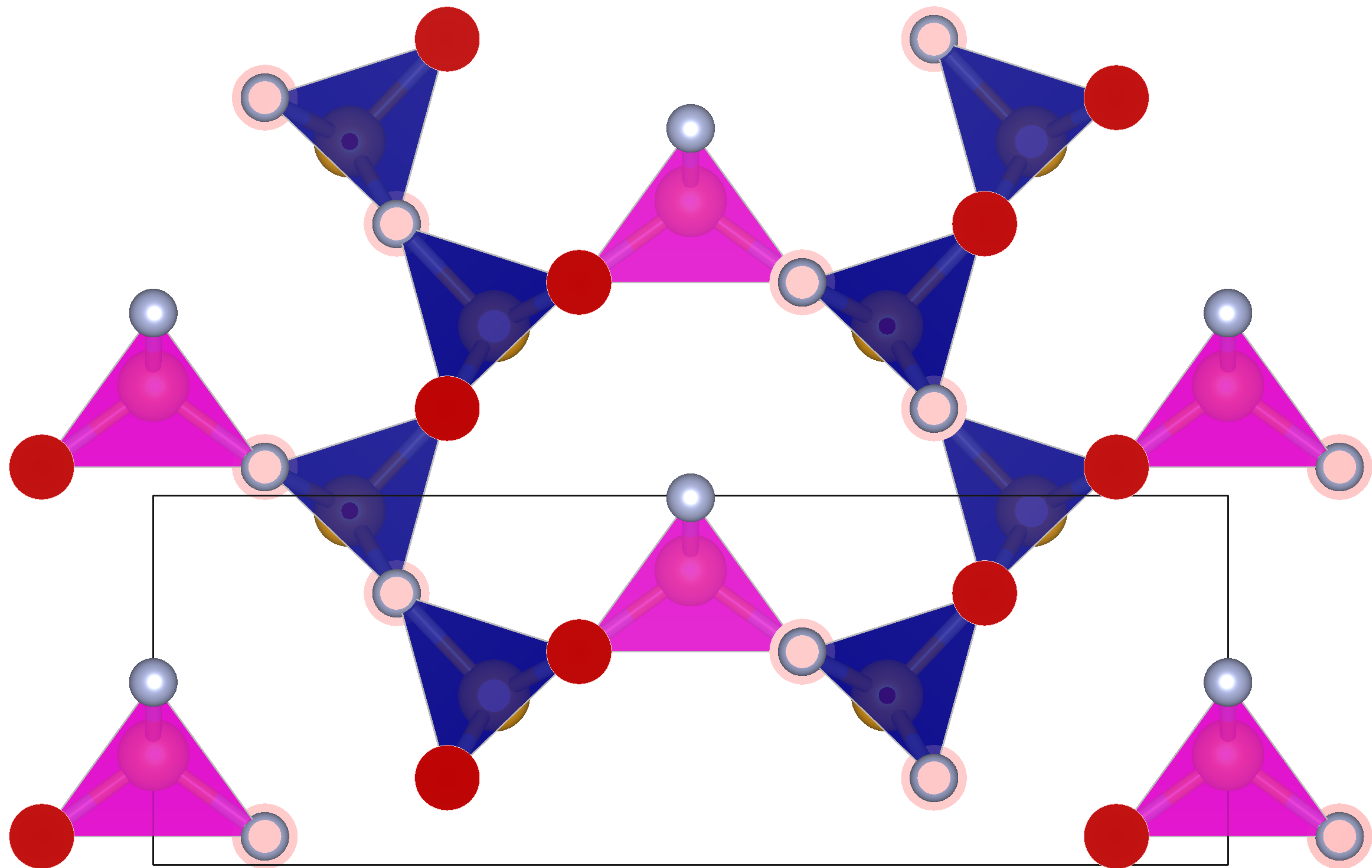

Supplement: Supplementary file 2 — ic2c01190_si_002.zip [file ic2c01190_si_002.zip › 18-A2.pdf]

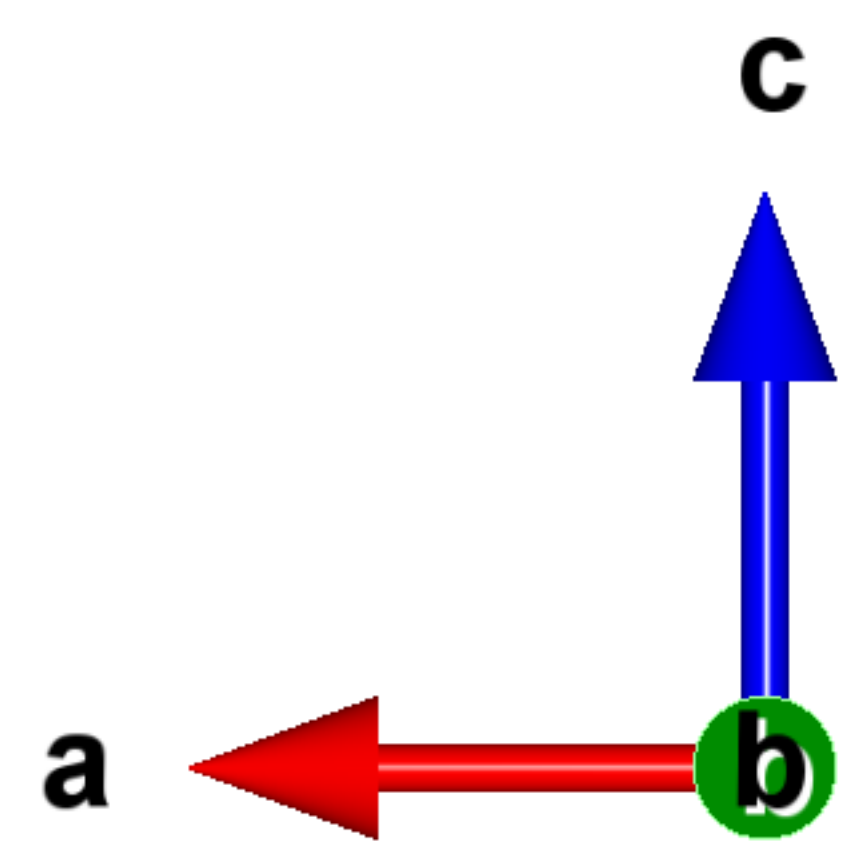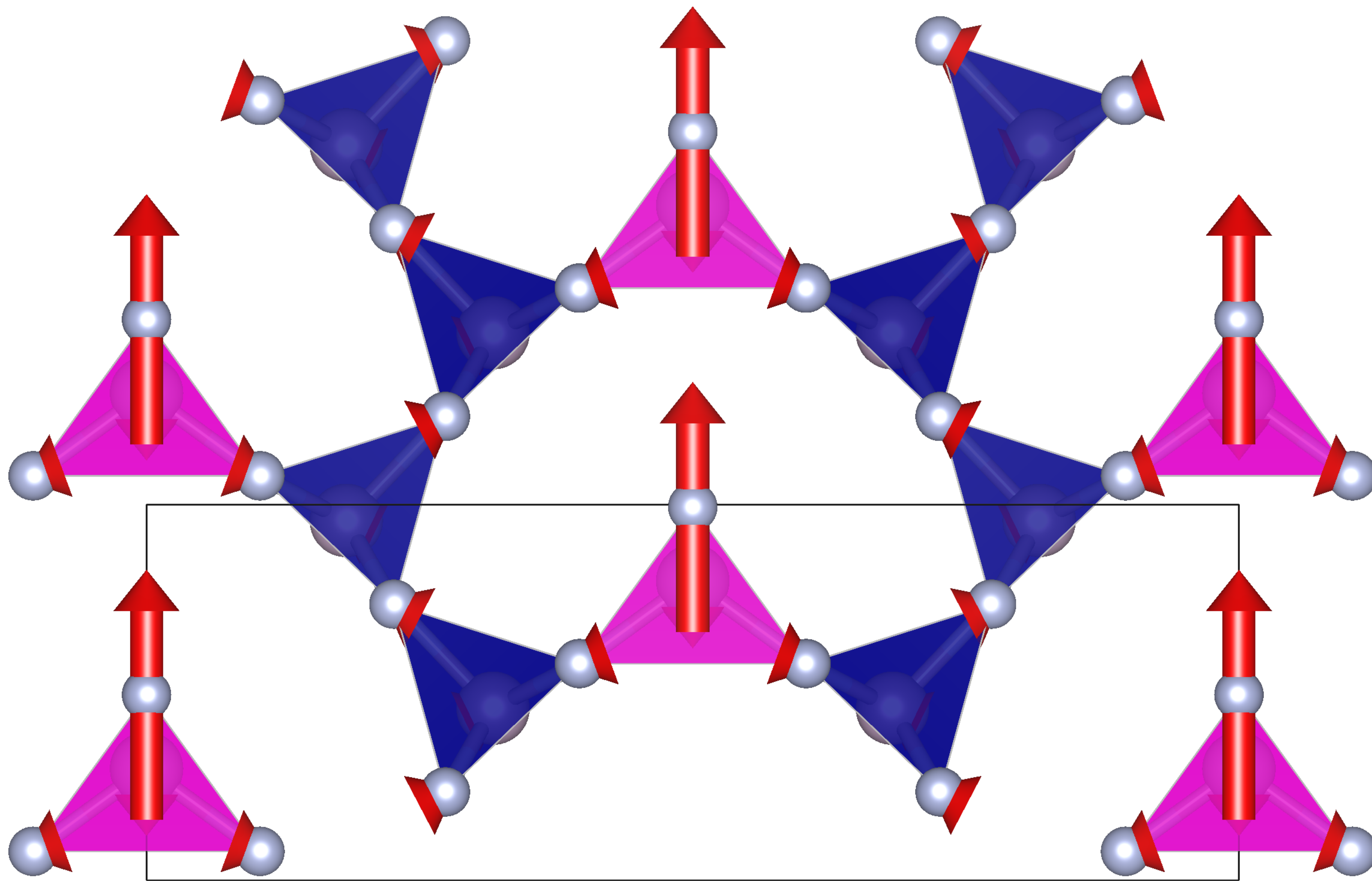

Supplement: Supplementary file 2 — ic2c01190_si_002.zip [file ic2c01190_si_002.zip › 19-A1.pdf]

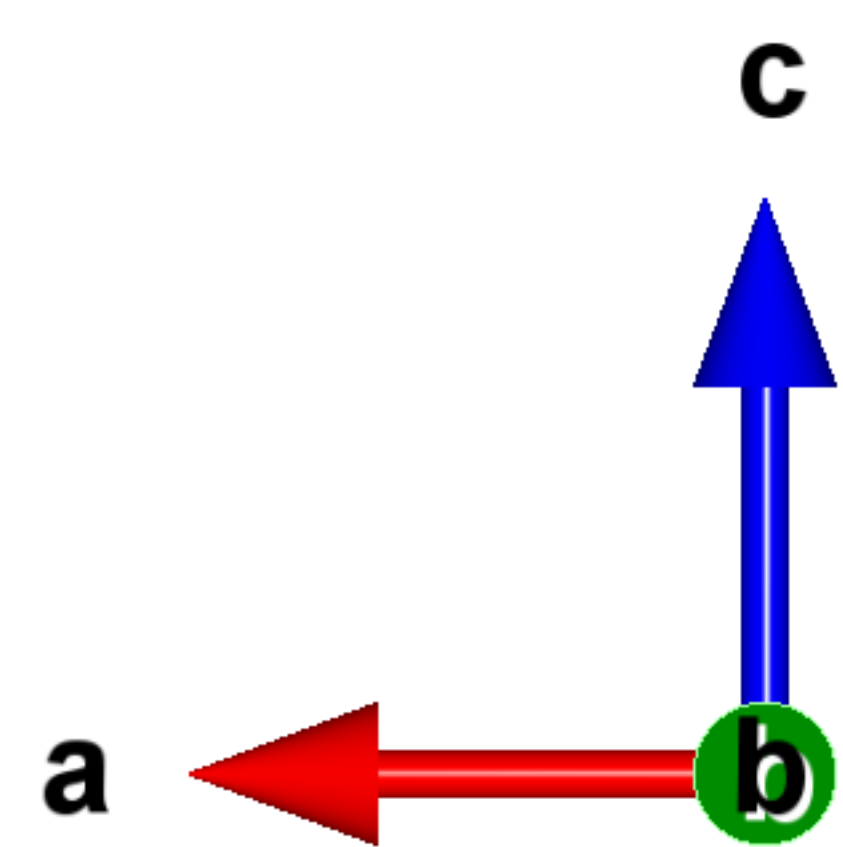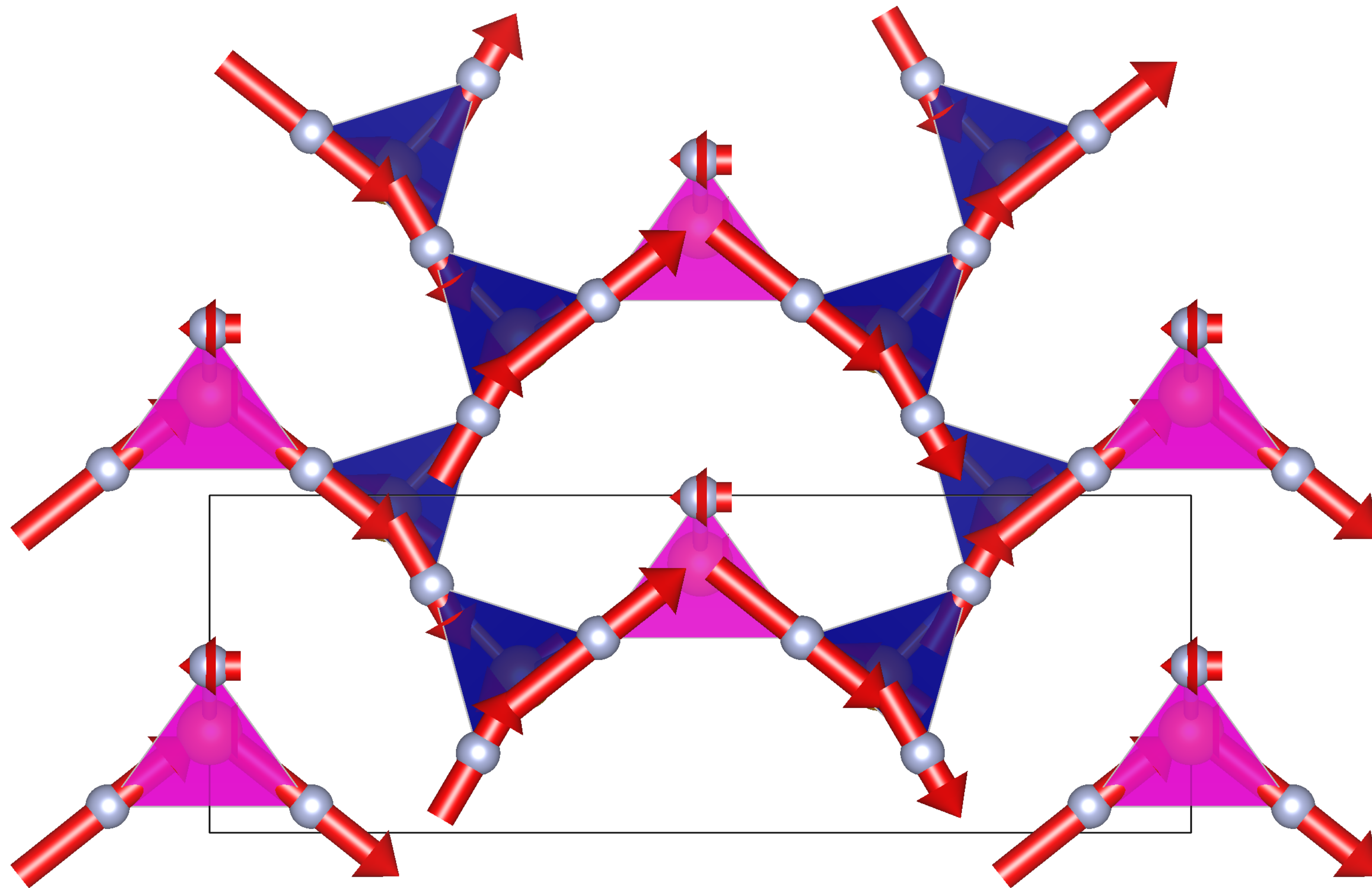

Supplement: Supplementary file 2 — ic2c01190_si_002.zip [file ic2c01190_si_002.zip › 20-B2.pdf]

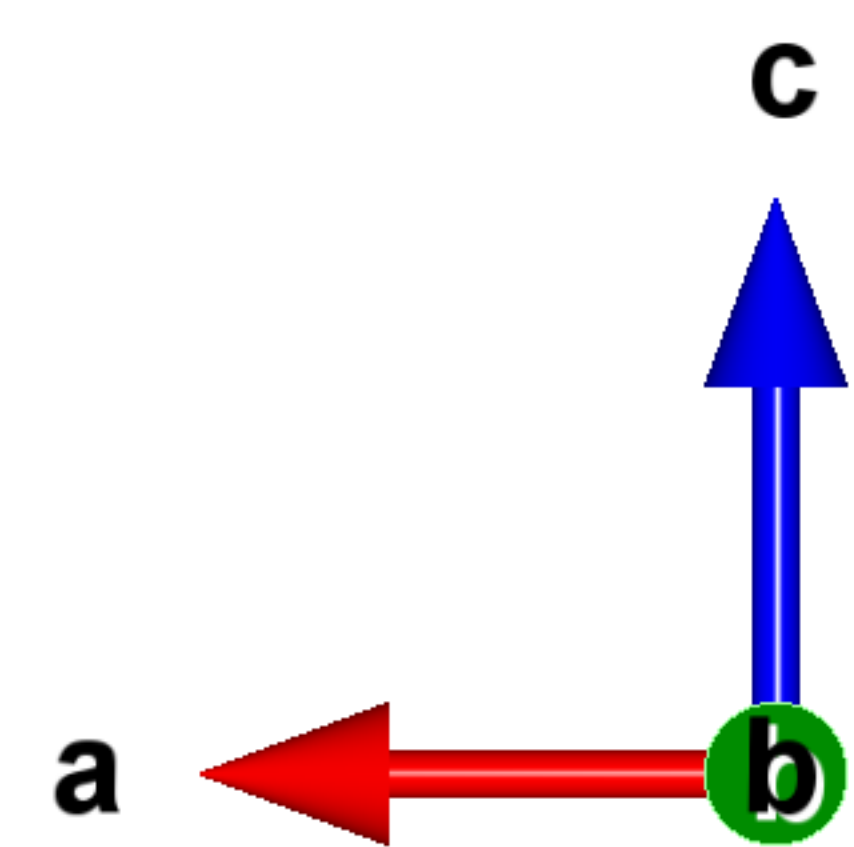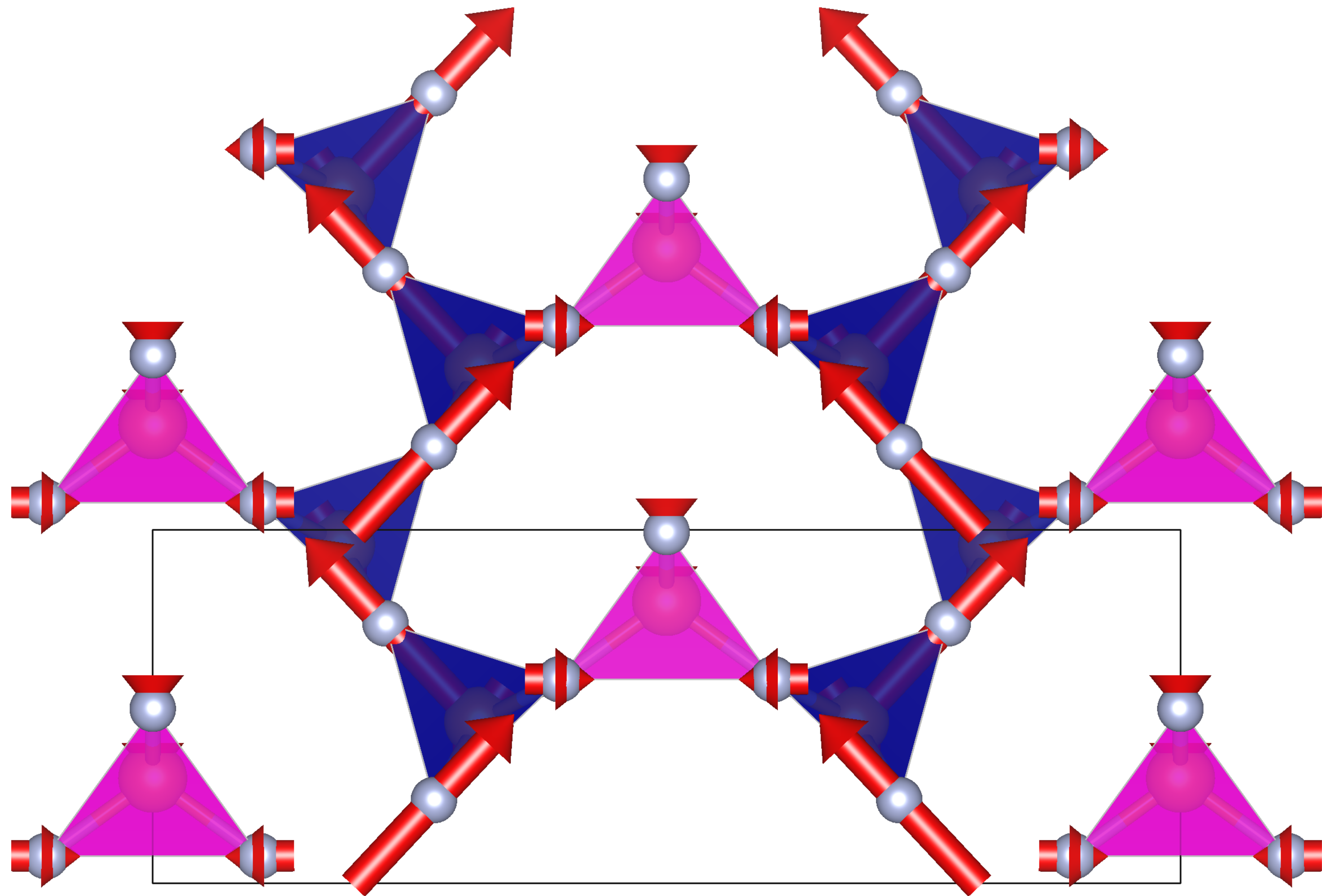

Supplement: Supplementary file 2 — ic2c01190_si_002.zip [file ic2c01190_si_002.zip › 21-A1.pdf]

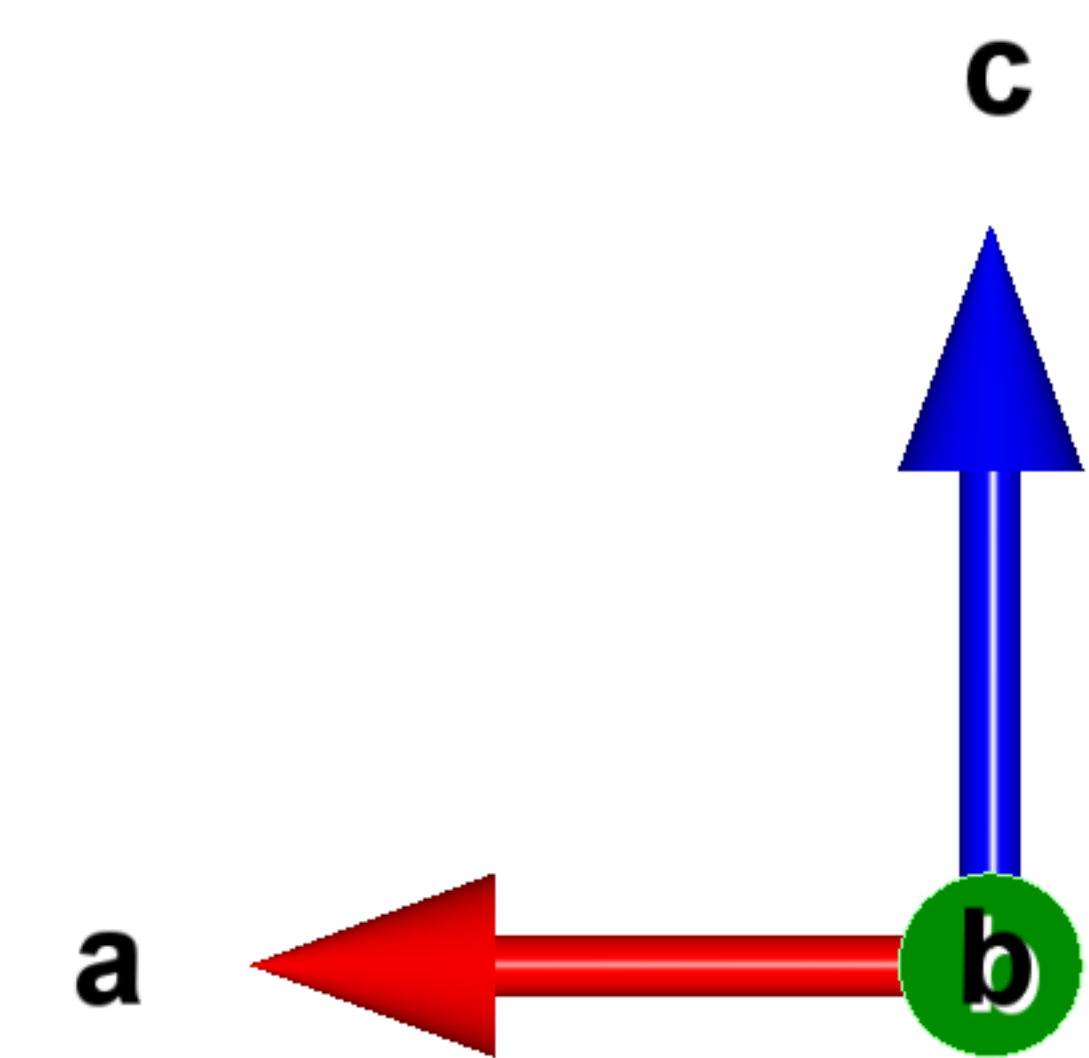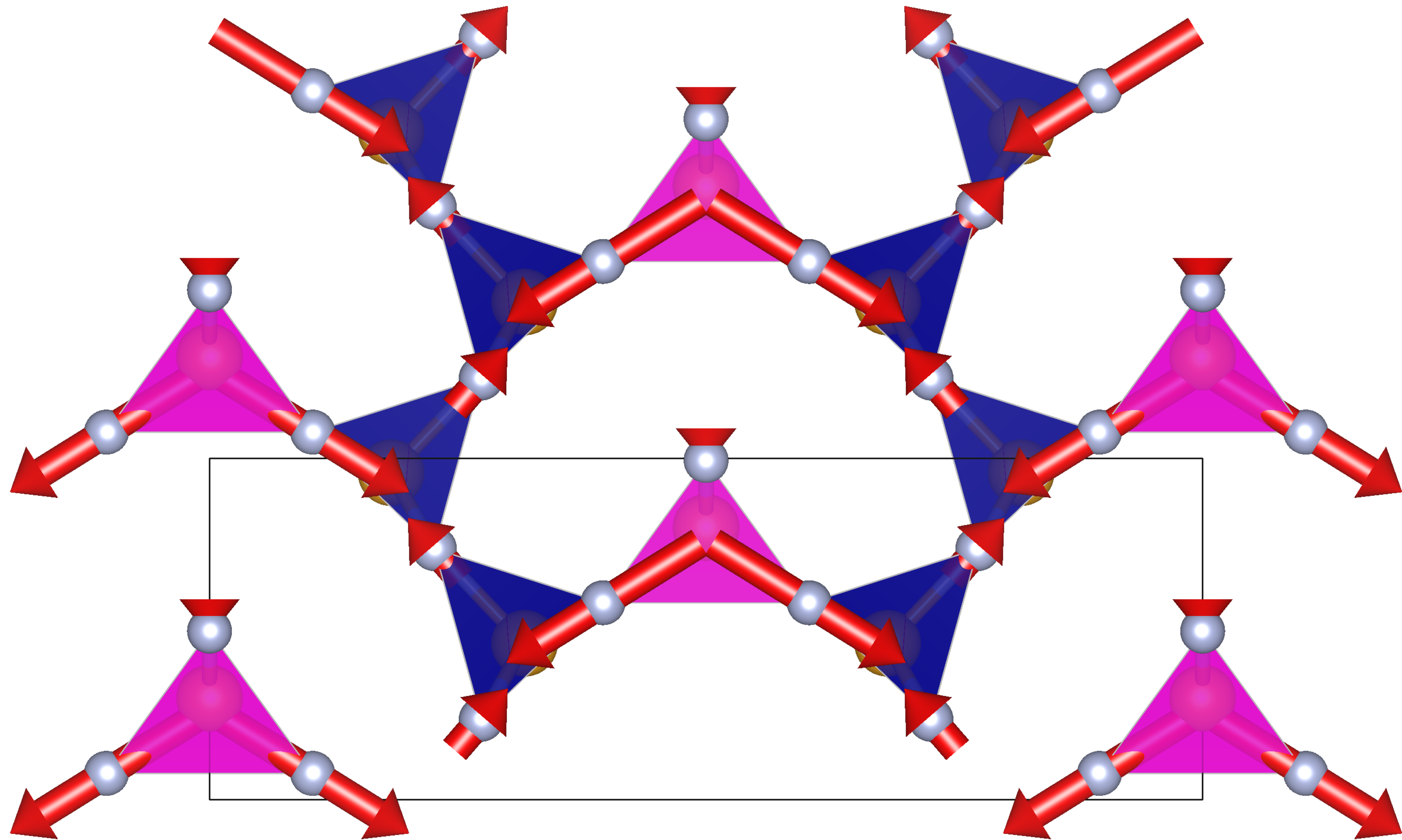

Supplement: Supplementary file 2 — ic2c01190_si_002.zip [file ic2c01190_si_002.zip › 22-A1.pdf]

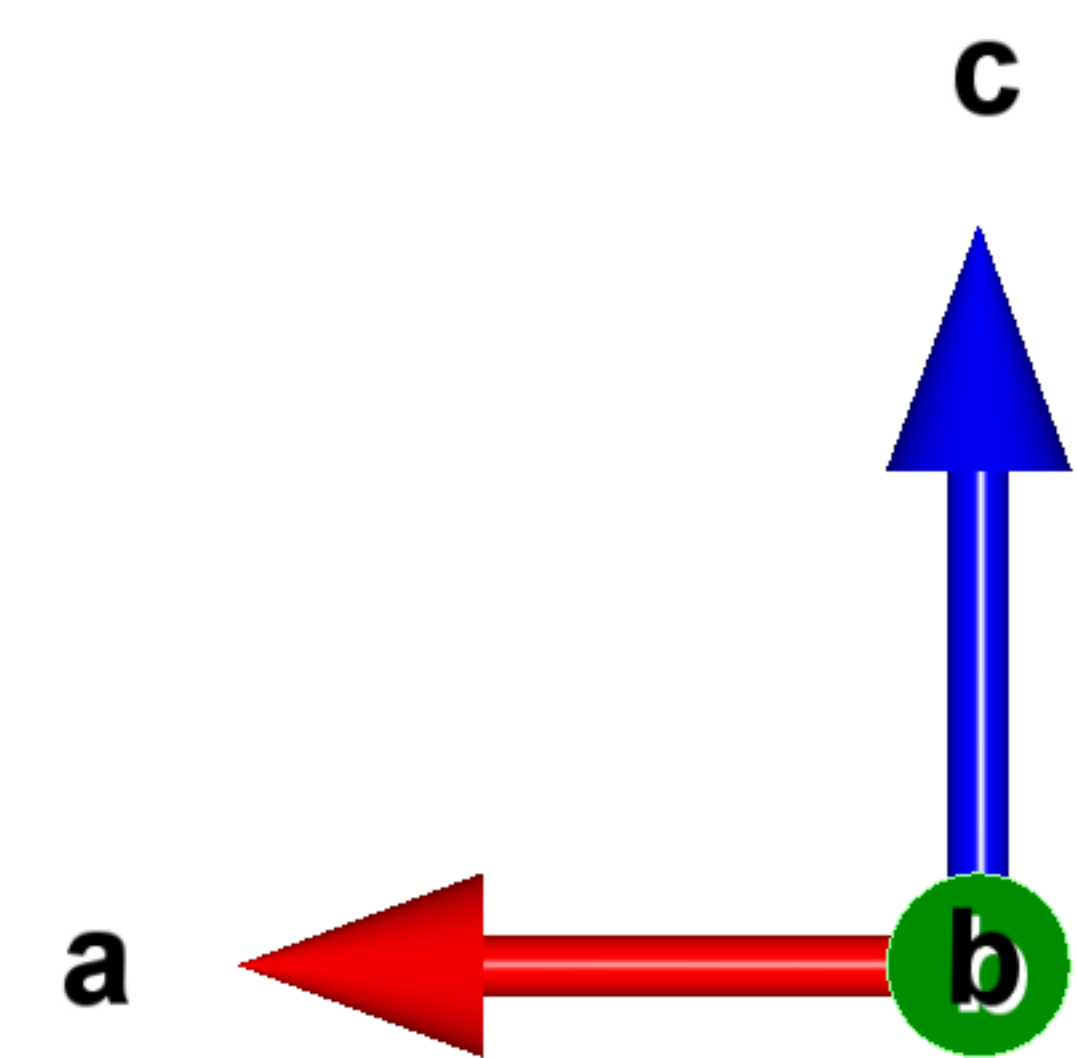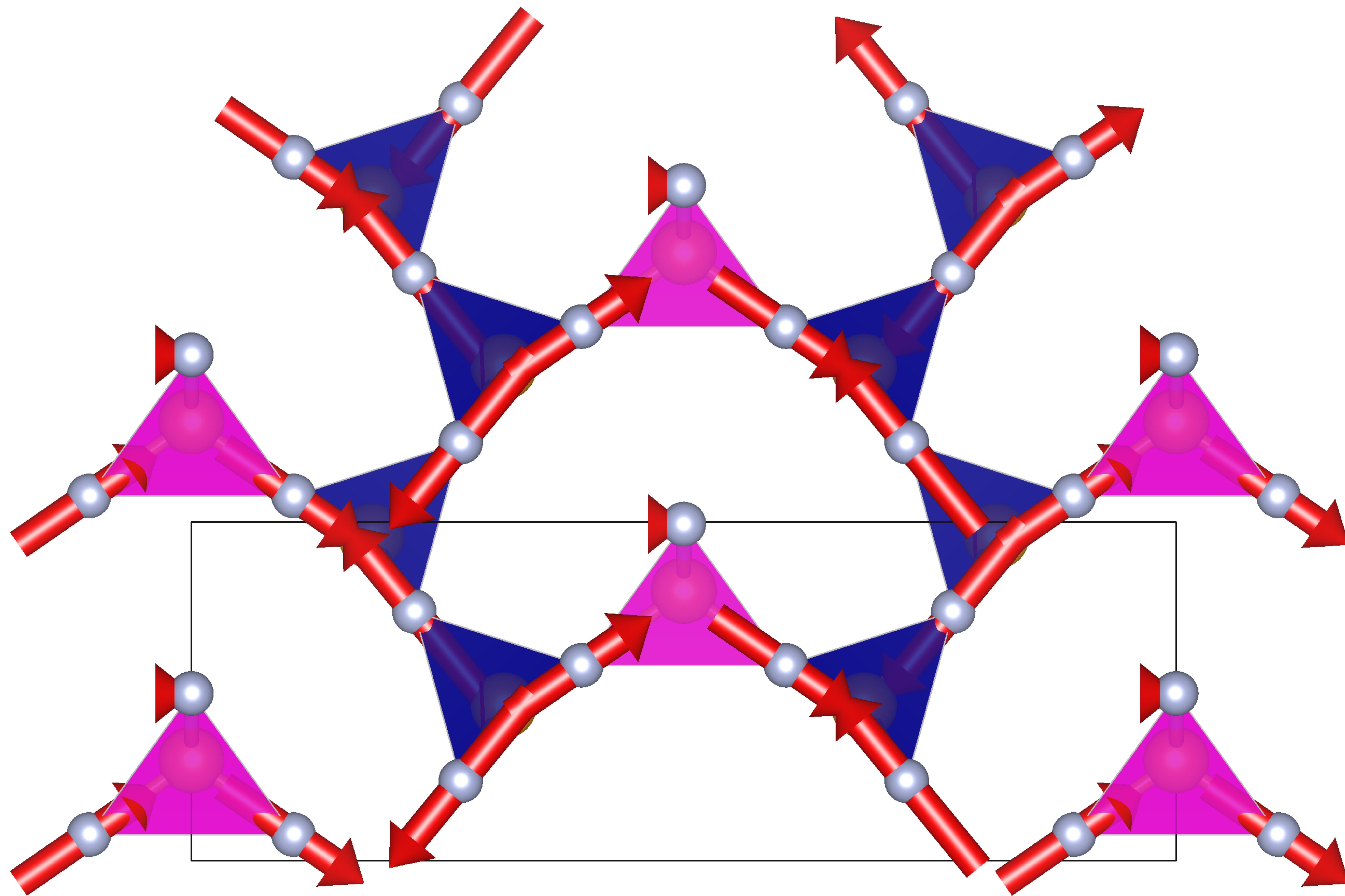

Supplement: Supplementary file 2 — ic2c01190_si_002.zip [file ic2c01190_si_002.zip › 23-B2.pdf]

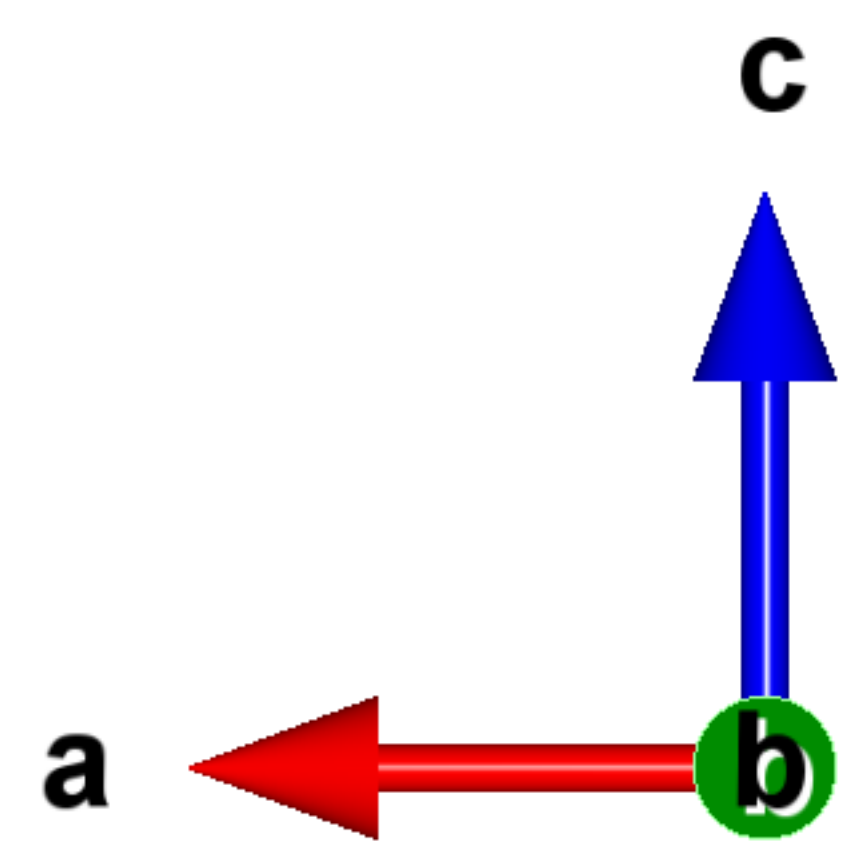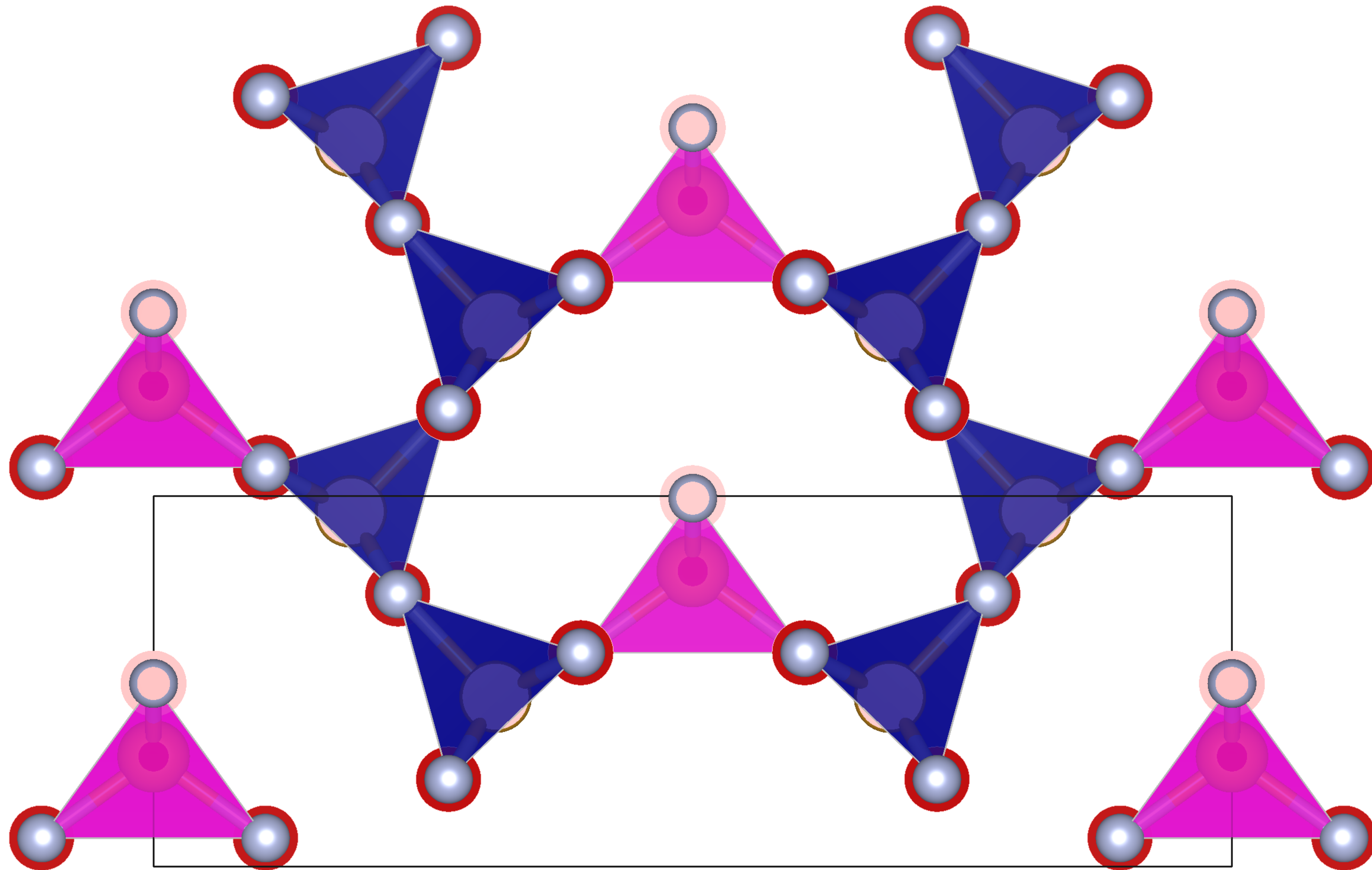

Supplement: Supplementary file 2 — ic2c01190_si_002.zip [file ic2c01190_si_002.zip › 24-B1.pdf]
